# Supplementary material for: Population differences in vaccine responses (POPVAC): scientific rationale and cross-cutting analyses for three linked, randomised controlled trials assessing the role, reversibility and mediators of immunomodulation by chronic infections in the tropics
Source: BMJ Open. 2021 Feb 16;11(2):e040425. doi: 10.1136/bmjopen-2020-040425 (PMC7893603; doi:10.1136/bmjopen-2020-040425)
Supplement: Supplementary data [file bmjopen-2020-040425supp003.pdf]

1

Table S1. Uganda National Expanded Programme on Immunisation (EPI) schedule

**Vaccine/ antigen**	**Dosage and doses required**	**Minimum Interval Between Doses**	**Minimum Age to Start**	**Mode and site of Administration**	**Storage Temperatures**
**Infant vaccines**					
BCG	Infants (0-11m) 0.05ml. ≥11 months and 0.1ml, 1 dose	Not applicable	At birth (or first contact)	*Intradermal*, right upper arm	$+2^0$C to $+8^0$C
DPT - HepB - Hib	0.5 ml, 3 doses	One month (4 weeks)	At 6 weeks or first contact after this age	*Intramuscular,* outer upper aspect of left thigh	$+2^0$C to $+8^0$C DO NOT FREEZE
PCV	0.5 mls, 3 doses	One month (4 weeks)	At 6 weeks or first contact after this age	*Intramuscular,* outer upper aspect of right thigh	$+2^0$C to $+8^0$C DO NOT FREEZE
Polio	2 drops, 3 doses	One month (4 weeks)	At birth or within the first 2 weeks (Polio 0) and at 6 weeks or first contact after 6 weeks (Polio 1)	*Orally*	$+2^0$C to $+8^0$C
IPV	0.5ml, 1 dose	Nil	At 14 weeks	*Intramuscular,* left upper thigh	$+2^0$C to $+8^0$C DO NOT FREEZE
Rotavirus	drops, 2 doses		6 weeks or 1st contact after this age	*Orally*	
Measles	0.5 ml, 1 doses	Nil	At 9 months (or first contact after that age).	*Subcutaneous,* left upper arm	$+2^0$C to $+8^0$C
Primary school/adolescent/adult vaccines					
Tetanus/Diphtheria	0.5 ml, 5 doses	Td1: First contact with a WCBA<br>Td2: One month after TT1<br>Td3: Six months after TT2<br>Td4: One year after TT3<br>Td5: One year after TT4	At first contact with a pregnant woman or women of childbearing age (15-49 years)	*Intramuscular,* upper arm	$+2^0$C to $+8^0$C, DO NOT FREEZE
HPV	0.5 ml, 2 doses	HPV1: First contact with a girl in primary 4, or aged 10 years and out of school<br>HPV2: Given at 6 months after HPV1[a]	Girls in primary 4 or 10-yearold girls who are out of school	*Intramuscular,* upper arm	$+2^0$C to $+8^0$C, DO NOT FREEZE

BCG: Bacillus Calmette Guerin. DPT: Diphtheria, Pertussis, Tetanus. Hep B: Hepatitis B. Hib: Haemophilus influenzae type B. PCV: pneumococcal conjugate vaccine. IPV: inactivated polio vaccine. HPV: Human Papilloma Virus. WCBA: woman of child-bearing age. [a]An additional dose of HPV, four weeks after the first dose, is recommended for girls aged 14 years or above receiving HPV immunisation for the first time.

22

Nkurunungi G, *et al. BMJ Open* 2021; 11:e040425. doi: 10.1136/bmjopen-2020-040425

23
