# Supplementary material for: Population differences in vaccine responses (POPVAC): scientific rationale and cross-cutting analyses for three linked, randomised controlled trials assessing the role, reversibility and mediators of immunomodulation by chronic infections in the tropics
Source: BMJ Open. 2021 Feb 16;11(2):e040425. doi: 10.1136/bmjopen-2020-040425 (PMC7893603; doi:10.1136/bmjopen-2020-040425)
Supplement: Supplementary data [file bmjopen-2020-040425supp004.pdf]

Table S2: Public Health England recommendations for giving more than one live attenuated vaccine in current use in the UK[26]

Vaccine combinations	Recommendations
Yellow Fever and Measles, Mumps, Rubella (MMR)	A four-week minimum interval period should be observed between the administration of these two vaccines. Yellow Fever and MMR should not be administered on the same day.
Varicella (and zoster) vaccine and MMR	If these vaccines are not administered on the same day, then a four-week minimum interval should be observed between vaccines.
Tuberculin skin testing (Mantoux) and MMR	If a tuberculin skin test has already been initiated, then MMR should be delayed until the skin test has been read unless protection against measles is required urgently. If a child has had a recent MMR, and requires a tuberculin test, then a fourweek interval should be observed.
All currently used live vaccines (BCG, rotavirus, live attenuated influenza vaccine (LAIV), oral typhoid vaccine, yellow fever, varicella, zoster and MMR) and tuberculin (Mantoux) skin testing.	Apart from those combinations listed above, these live vaccines can be administered at any time before or after each other. This includes tuberculin (Mantoux) skin testing.
