# Supplementary material for: Population differences in vaccine responses (POPVAC): scientific rationale and cross-cutting analyses for three linked, randomised controlled trials assessing the role, reversibility and mediators of immunomodulation by chronic infections in the tropics
Source: BMJ Open. 2021 Feb 16;11(2):e040425. doi: 10.1136/bmjopen-2020-040425 (PMC7893603; doi:10.1136/bmjopen-2020-040425)

Figure S1. Causal diagram for assessment of whether exposure to parasites mediates differences in vaccine response between urban and rural settings

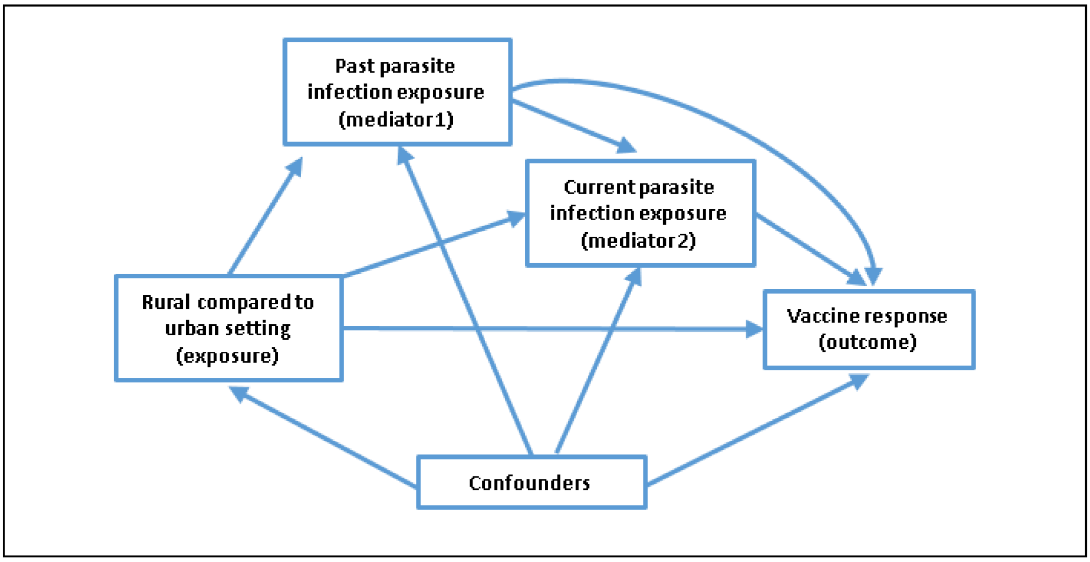

Supplement: Supplementary data [file bmjopen-2020-040425supp002.pdf]
