# Supplementary material for: Population differences in vaccine responses (POPVAC): scientific rationale and cross-cutting analyses for three linked, randomised controlled trials assessing the role, reversibility and mediators of immunomodulation by chronic infections in the tropics
Source: BMJ Open. 2021 Feb 16;11(2):e040425. doi: 10.1136/bmjopen-2020-040425 (PMC7893603; doi:10.1136/bmjopen-2020-040425)
Supplement: Author's manuscript [file bmjopen-2020-040425.draft_revisions.pdf]

BMJ Open

BMJ Open is committed to open peer review. As part of this commitment we make the peer review history of every article we publish publicly available.

When an article is published we post the peer reviewers' comments and the authors' responses online. We also post the versions of the paper that were used during peer review. These are the versions that the peer review comments apply to.

The versions of the paper that follow are the versions that were submitted during the peer review process. They are not the versions of record or the final published versions. They should not be cited or distributed as the published version of this manuscript.

BMJ Open is an open access journal and the full, final, typeset and author-corrected version of record of the manuscript is available on our site with no access controls, subscription charges or pay-per-view fees (http://bmjopen.bmj.com).

If you have any questions on BMJ Open's open peer review process please email info.bmjopen@bmj.com

BMJ Open

Population differences in vaccine responses (POPVAC): scientific rationale and cross-cutting analyses for three linked, randomised controlled trials assessing the role, reversibility and mediators of immunomodulation by chronic infections in the tropics

Journal:	*BMJ Open*
Manuscript ID	bmjopen-2020-040425
Article Type:	Protocol
Date Submitted by the Author:	13-May-2020
Complete List of Authors:	Nkurunungi, Gyaviira; Medical Research Council/Uganda Virus Research Institute and London School of Hygiene and Tropical Medicine (MRC/UVRI and LSHTM) Uganda Research Unit, Immunomodulation and Vaccines Programme<br>Zirimenya , Ludoviko ; Medical Research Council/Uganda Virus Research Institute and London School of Hygiene and Tropical Medicine (MRC/UVRI and LSHTM) Uganda Research Unit, Immunomodulation and Vaccines Programme<br>Natukunda , Agnes ; Medical Research Council/Uganda Virus Research Institute and London School of Hygiene and Tropical Medicine (MRC/UVRI and LSHTM) Uganda Research Unit, Immunomodulation and Vaccines Programme<br>Nassuuna , Jacent; Medical Research Council/Uganda Virus Research Institute and London School of Hygiene and Tropical Medicine (MRC/UVRI and LSHTM) Uganda Research Unit, Immunomodulation and Vaccines Programme<br>Oduru, Gloria; Medical Research Council/Uganda Virus Research Institute and London School of Hygiene and Tropical Medicine (MRC/UVRI and LSHTM) Uganda Research Unit, Immunomodulation and Vaccines Programme<br>Ninsiima , Caroline ; Medical Research Council/Uganda Virus Research Institute and London School of Hygiene and Tropical Medicine (MRC/UVRI and LSHTM) Uganda Research Unit, Immunomodulation and Vaccines Programme<br>Zziwa , Christopher ; Medical Research Council/Uganda Virus Research Institute and London School of Hygiene and Tropical Medicine (MRC/UVRI and LSHTM) Uganda Research Unit, Immunomodulation and Vaccines Programme<br>Akello , Florence ; Medical Research Council/Uganda Virus Research Institute and London School of Hygiene and Tropical Medicine (MRC/UVRI and LSHTM) Uganda Research Unit, Immunomodulation and Vaccines Programme<br>Kizindo , Robert ; Medical Research Council/Uganda Virus Research Institute and London School of Hygiene and Tropical Medicine (MRC/UVRI and LSHTM) Uganda Research Unit, Immunomodulation and Vaccines Programme<br>Akello, Mirriam; Medical Research Council/Uganda Virus Research

Institute and London School of Hygiene and Tropical Medicine (MRC/UVRI and LSHTM) Uganda Research Unit, Immunomodulation and Vaccines Programme
Kaleebu, Pontiano; Medical Research Council/Uganda Virus Research Institute and London School of Hygiene and Tropical Medicine (MRC/UVRI and LSHTM) Uganda Research Unit
Wajja, Anne; Medical Research Council/Uganda Virus Research Institute and London School of Hygiene and Tropical Medicine (MRC/UVRI and LSHTM) Uganda Research Unit, Immunomodulation and Vaccines Programme
Luzze, Henry; Uganda National Expanded Program on Immunisation, Ministry of Health
Cose , Stephen ; Medical Research Council/Uganda Virus Research Institute and London School of Hygiene and Tropical Medicine (MRC/UVRI and LSHTM) Uganda Research Unit, Immunomodulation and Vaccines Programme; London School of Hygiene and Tropical Medicine Department of Clinical Research
Webb, Emily; London School of Hygiene and Tropical Medicine, MRC Tropical Epidemiology Group, Department of Infectious Disease Epidemiology,
Elliott, Alison ; Medical Research Council/Uganda Virus Research Institute and London School of Hygiene and Tropical Medicine (MRC/UVRI and LSHTM) Uganda Research Unit, Immunomodulation and Vaccines Programme; London School of Hygiene and Tropical Medicine Department of Clinical Research

Keywords: Infection control < INFECTIOUS DISEASES, PARASITOLOGY, PUBLIC HEALTH, Immunology < TROPICAL MEDICINE, Epidemiology < TROPICAL MEDICINE, Paediatric infectious disease & immunisation < PAEDIATRICS

SCHOLARONE™
Manuscripts

I, the Submitting Author has the right to grant and does grant on behalf of all authors of the Work (as defined in the below author licence), an exclusive licence and/or a non-exclusive licence for contributions from authors who are: i) UK Crown employees; ii) where BMJ has agreed a CC-BY licence shall apply, and/or iii) in accordance with the terms applicable for US Federal Government officers or employees acting as part of their official duties; on a worldwide, perpetual, irrevocable, royalty-free basis to BMJ Publishing Group Ltd ("BMJ") its licensees and where the relevant Journal is co-owned by BMJ to the co-owners of the Journal, to publish the Work in this journal and any other BMJ products and to exploit all rights, as set out in our licence.

The Submitting Author accepts and understands that any supply made under these terms is made by BMJ to the Submitting Author unless you are acting as an employee on behalf of your employer or a postgraduate student of an affiliated institution which is paying any applicable article publishing charge ("APC") for Open Access articles. Where the Submitting Author wishes to make the Work available on an Open Access basis (and intends to pay the relevant APC), the terms of reuse of such Open Access shall be governed by a Creative Commons licence – details of these licences and which Creative Commons licence will apply to this Work are set out in our licence referred to above.

Other than as permitted in any relevant BMJ Author's Self Archiving Policies, I confirm this Work has not been accepted for publication elsewhere, is not being considered for publication elsewhere and does not duplicate material already published. I confirm all authors consent to publication of this Work and authorise the granting of this licence.

For peer review only

Population differences in vaccine responses (POPVAC): scientific rationale and cross-cutting analyses for three linked, randomised controlled trials assessing the role, reversibility and mediators of immunomodulation by chronic infections in the tropics

Gyaviira Nkurunungi[1,¶,*], Ludoviko Zirimenya[1,¶], Agnes Natukunda[1,¶], Jacent Nassuuna[1], Gloria Oduru[1], Caroline Ninsiima[1], Christopher Zziwa[1], Florence Akello[1], Robert Kizindo[1], Mirriam Akello[1], Pontiano Kaleebu[1], Anne Wajja[1], Henry Luzze[2], Stephen Cose[1,3], Emily L Webb[4], Alison M Elliott[1,5] **for the POPVAC trial team**

[1]Immunomodulation and Vaccines Programme, Medical Research Council/Uganda Virus Research Institute and London School of Hygiene and Tropical Medicine (MRC/UVRI and LSHTM) Uganda Research Unit, Entebbe, Uganda

[2]Uganda National Expanded Program on Immunisation, Ministry of Health, Kampala, Uganda

[3]Department of Clinical Research, London School of Hygiene and Tropical Medicine, London, United Kingdom

[4]MRC Tropical Epidemiology Group, Department of Infectious Disease Epidemiology, London School of Hygiene and Tropical Medicine, London, United Kingdom

¶These authors contributed equally

***Correspondence:** Gyaviira Nkurunungi; gyaviira.nkurunungi@lshtm.ac.uk

For peer review only

Abstract

Introduction

Vaccine-specific immune responses vary between populations and are often impaired in low-income, rural settings. Drivers of these differences are not fully elucidated, hampering identification of strategies for optimising vaccine effectiveness. We hypothesise that urban-rural (and regional and international) differences in vaccine responses are mediated to an important extent by differential exposure to chronic infections, particularly parasitic infections.

Methods and analysis

Three related trials sharing core elements of study design and procedures (allowing comparison of outcomes across the trials) will test the effects of 1) individually randomised intervention against schistosomiasis (Trial A) and malaria (Trial B), and 2) BCG re-vaccination (Trial C), on a common set of vaccine responses. We will enrol adolescents from Ugandan schools in rural high-schistosomiasis (Trial A) and rural high-malaria (Trial B) settings, and from an established urban birth cohort (Trial C). All participants will receive BCG on day 'zero'; yellow fever, oral typhoid and Human Papilloma Virus (HPV) vaccines at week 4; and HPV and Tetanus/diphtheria booster vaccine at week 28. Primary outcomes are BCG-specific IFN-γ responses (eight weeks after BCG) and for other vaccines, antibody responses to key vaccine antigens at four weeks after immunisation. Secondary analyses will determine effects of interventions on correlates of protective immunity, vaccine response waning, priming versus boosting immunisations, and parasite infection status and intensity. Overarching analyses will compare outcomes between the three trial settings. Sample archives will offer opportunities for exploratory evaluation of the role of immunological and 'trans-kingdom' mediators in parasite modulation of vaccine-specific responses.

Ethics and dissemination

Ethics approval has been obtained from relevant Ugandan and UK ethics committees. Results will be shared with Uganda Ministry of Health, relevant district councils, community leaders and study participants. Further dissemination will be done through conference proceedings and publications.

Trial registration

Current Controlled Trials identifiers: Trial A, ISRCTN60517191; Trial B, ISRCTN62041885; Trial C, ISRCTN10482904.

Article summary

For peer review only

Strengths and limitations of this study

- This will be the first well-powered programme of work to investigate effects of schistosomiasis treatment, of malaria treatment, and of BCG revaccination on vaccine responses in adolescents.
- A major strength of this work is the opportunity to synthesise findings from three different study settings with differential parasite exposure using causal mediation analysis to obtain a comprehensive understanding of how parasitic infections influence vaccine responses in human populations.
- The results will provide insight into effects of parasites on infectious disease susceptibility: immunisation, notably with live vaccines, offers a surrogate for infection challenge in human subjects.
- The sample archives developed will provide a major asset for exploration of new leads arising from this hypothesis-driven work, or for an alternative, "systems biology" approach investigating (for example) transcriptome, microbiome and virome.
- One limitation is that observational analyses of parasite effects are subject to potential unmeasured confounding; this will be mitigated by cautious interpretation of results and our intervention studies will address causality rigorously.

Word count

3922

Keywords

Vaccine; parasite; BCG; malaria; schistosomiasis

For peer review only

Introduction

Population differences in vaccine responses

Effective vaccines are key weapons against infectious diseases,[1] but are still lacking for many poverty-related, neglected, emerging and re-emerging infections. Vaccine responses vary between populations and are often impaired in low-income, rural settings.[2-6] A notable example is Bacillus Calmette–Guérin (BCG): both vaccine response and efficacy against tuberculosis differ internationally[3 4] and regionally.[6 7] Among other vaccines, yellow fever vaccine induced lower neutralising antibody levels, and responses waned faster, in Uganda compared to Switzerland.[5] Oral rotavirus and polio vaccines, are also affected.[2] Within-country, influenza and tetanus responses differed between urban and rural Gabon.[8 9] Responses to candidate tuberculosis,[10] malaria[11] and Ebola[12] vaccines are lower in Africa than in Europe or America. Prior exposure to the pathogen targeted by the vaccine, or to related organisms, may contribute to this phenomenon, but recent analyses implicate broader "environmental sensitisation"[6] the drivers of which have not been determined. Prior exposure cannot explain results for vaccines against rare organisms, such as Ebola. Thus, drivers of population differences in vaccine response are not fully elucidated; improved understanding is important for effective vaccine development and implementation.

The POPulation differences in VACcine responses (POPVAC) programme comprises three trials, A, B and C, designed to address this challenge. The trials are presented separately in this issue. This "Protocol X" provides an overview of our hypotheses and objectives.

Immunomodulation by parasitic infections

Parasitic infections are important in tropical low-income countries (LICs),[13-15] and long proposed as modulators of vaccine responses.[16-19] As detailed in POPVAC A and B protocols, animal models[20-22] and observational human studies[23-25] support this hypothesis, but no well-powered trials have been conducted to evaluate causality and reversibility of parasite effects on vaccine responses in adolescents or adults.[26]

Trained immunity

Exposure to other unrelated infections or microbial antigens may also contribute to "environmental sensitisation", modulating vaccine responses through training of the innate immune system.[27] BCG immunisation is a key model for this effect, as considered in Protocol C.[28 29]

The "trans-kingdom" concept

The "trans-kingdom" concept[30] emphasises that mammals support a complex ecosystem of bacteria, fungi, protozoa, metazoa and viruses, and suggests that these interact in their effects on the mammalian immune system.[30] For example, in a mouse model, infection with the gut helminth *Heligmosomoides polygyrus*, or

For peer review only - http://bmjopen.bmj.com/site/about/guidelines.xhtml

exposure to schistosome eggs, activated latent herpesvirus infection via alternative macrophage activation and the IL-4/Stat6 pathway.[31] In a contrasting study, *H. polygyrus* caused enhanced responses to Respiratory Syncytial Virus (RSV) in the mouse lung through interaction with the gut microbiome, translocation of microbial products to the circulation and enhanced systemic type I interferon expression.[32] Interestingly, in these studies, the same helminth resulted in opposite outcomes for latent viruses (for which it induced activation), versus exogenous viruses (for which it improved control).

Little has been done to evaluate these phenomena in human populations but, regarding latent herpesviruses our studies show that parasite exposure associates with elevated Kaposi's Sarcoma Herpesvirus antibody prevalence and titre (indicating viral activation).[33-35] The impact of malaria on Epstein Barr Virus (EBV), promoting induction of Burkitt's lymphoma, is well recognised.[36] Cytomegalovirus (CMV) has major immunological effects, including impact on vaccine responses.[37]

In humans, evidence of enhanced microbial translocation (MT) has been found during *S. mansoni*,[38] hookworm[39] and *Strongyloides*[40] infection, with altered expression of parameters such as toll-like receptor expression, but without the level of immune activation associated with septic shock or with MT in HIV infection. Nevertheless, the second mouse model discussed above shows that this may have profound effects on responses to infectious agents at remote sites.[32]

Thus, herpesvirus activation and, or, MT mediate, in part, parasite-induced modulation of vaccine responses.

Differences in immunological characteristics between populations

Whatever the key exposures and mechanisms involved, immunological characteristics differ markedly between populations internationally,[5 41 42] and between urban and rural settings.[41 43 44] Characteristics that differ include gene methylation and expression (not solely attributable to population genetics);[43 45] responses to innate stimuli;[42 45] frequency and activation of innate immune cells, T and B cells, and memory cell pools.[5 41] Understanding the immunological predictors of vaccine response, and factors that drive them, will contribute to strategies for improving vaccine efficacy for rural, tropical settings.

For peer review only - http://bmjopen.bmj.com/site/about/guidelines.xhtml

Hypothesis and objectives

The overarching goal of the 'POPulation differences in VACcine responses' programme (POPVAC) is to understand population differences in vaccine responses, in order to identify strategies through which vaccine effectiveness can be optimised for low-income, tropical settings where they are especially needed. We focus on the hypothesis (**Figure 1**) that *geographical differences in vaccine responses are mediated to an important extent by differential exposure to chronic, particularly parasitic, infections; that parasites act in part via "trans-kingdom" effects; and that these exposures impact the pre-immunisation immune profile and hence vaccine response (and efficacy)"*.

We will address this hypothesis in three, linked trials (POPVAC A, B and C; detailed protocols published separately in this issue) which share core elements of study design and procedures, allowing comparison of outcomes across the trials. Each trial will test effects of a different randomised intervention on a common set of vaccine responses. **POPVAC A** will determine the effect of intensive schistosomiasis treatment on vaccine responses among rural island adolescents. **POPVAC B** will determine the effect of intensive malaria treatment on vaccine responses among rural adolescents. **POPVAC C** will determine the effect of BCG re-vaccination on responses to unrelated vaccines.

This paper describes background and methods common to all three trials, summarises objectives linking the trials, and details planned approaches to cross-cutting objectives which will use data and samples from all trials. SPIRIT reporting guidelines[46] are used.

Our objectives are to

i. Determine whether there are reversible effects of chronic parasitic infection on vaccine response (**POPVAC A and B**).
ii. Determine whether BCG "pre-immunisation" enhances responses to unrelated vaccines (**POPVAC C**).
iii. Determine which life-course exposures influence vaccine responses in adolescence (using data from **POPVAC C**).
iv. Compare vaccine response profiles between three Ugandan settings (using data from all Trials): rural, high schistosomiasis exposure; rural, high malaria exposure; and urban.
v. Explore the role of "trans-Kingdom" interactions in determining vaccine responses (using samples and data from all Trials).
vi. Investigate pre-immunisation immunological parameters associated with vaccine responses and determine whether these are driven by parasite or microbial exposure (using samples and data from all Trials).

For peer review only - http://bmjopen.bmj.com/site/about/guidelines.xhtml

Methods and analysis

Setting

Uganda still experiences high schistosomiasis[47 48] and malaria burdens.[49-51] Our study sites (**Figure 2**) will be Lake Victoria Koome Islands (high-schistosomiasis),[47 52] Jinja district rural sub-counties (high-malaria)[51] and Entebbe (urban; low-schistosomiasis and low-malaria).[52] These settings provide ideal opportunities to investigate effects of schistosomiasis and malaria on vaccine responses. Geohelminths are less common in our settings:[47 53] hookworm, especially, has declined dramatically following government intervention programmes; therefore geohelminths will be considered as potential confounders, where appropriate, but not prioritised.

Cohorts

This work will involve adolescents from two rural cohorts and one urban cohort (**Figure 3**). Rural cohorts (Koome islands and Jinja district) will be school based, recruiting participants aged 9 to 17 years from primary schools selected purposefully to assure schistosomiasis and malaria prevalence appropriate to our design. This will allow us to investigate our two major infections separately, averting the risk that a potent effect of one infection masks an impact of the other, or of its treatment.

The urban cohort will recruit members of the Entebbe Mother and Baby Study (EMaBS) birth cohort:[54] 2500 women were recruited between 2003 and 2005 in a trial of anthelminthic treatment during pregnancy, investigating effects on infants' vaccine responses.[54] EMaBS participants will be aged 13 to 17 years during recruitment to this study; about 300 individuals are expected to take part.

Interventions

In the high-schistosomiasis cohort (Trial A) we will individually randomise participants to intensive or standard praziquantel (PZQ) treatment, in a 1:1 ratio.

In the high-malaria cohort (Trial B) we will individually randomise participants to monthly dihydroartemisinin-piperaquine (DP) versus placebo in a double-blind, placebo-controlled trial.

In the urban (EMaBS) cohort (Trial C) we will individually randomise participants to BCG re-vaccination, or no BCG re-vaccination, as the first component of the vaccine schedule.

Recruitment criteria, interventions, randomisation and treatment allocation procedures are detailed in protocols for Trials A, B and C (published in this issue).

For peer review only - http://bmjopen.bmj.com/site/about/guidelines.xhtml

Immunisations

We have previously highlighted the complexity of helminth effects on vaccine responses.[26] Differences in parasite effects on live and non-live, oral and parenteral, priming and boosting vaccines may contribute to this. Activated innate responses may kill live vaccines and suppress subsequent adaptive responses,[5 55] but bias, or enhance, responses to toxoids or proteins;[17 23 56] intestinal inflammation may impair responses to live oral vaccines;[57] priming may be more vulnerable than boosting.[58 59] Thus, results from a single-vaccine study would not be generalisable.

Therefore, we will study a portfolio of licensed vaccines expected to be beneficial (some already given) to adolescents in Uganda: live parenteral (BCG, yellow fever), live oral (typhoid) and non-live (HPV [a viral particle vaccine] and tetanus/diphtheria [Td; toxoid vaccines]). This will allow us to compare effects of interventions and exposures between vaccine types. Each cohort will receive the same vaccine portfolio (**Table 1**), comprising three main immunisation days (weeks 0, 4 and 28). Additional HPV immunisation will be provided for girls aged 14 years or above, and a second tetanus/diphtheria boost will be given after study completion, to accord with national Expanded Programme on Immunisation (EPI) routines, but responses to these will not specifically be addressed. Further rationale for vaccine selection is detailed in Supplementary information. Our schedule has been developed in consultation with the EPI programme (Table S1) and is cognizant of potential interference between vaccines (Supplementary information).

Although optimal timings for outcome measures vary between vaccines, sampling (for primary endpoints) will be done at eight weeks post BCG, four weeks post YF-17D, Ty21a, HPV and Td (**Figure 4**), targeting establishment of memory responses and antibody response peaks. A secondary endpoint at one year will assess waning. Analyses will take baseline measurements into account.

Table 1. Immunisation schedule

	**Immunisation week 0**	**Immunisation week 4**	**[Immunisation week 8]**	**Immunisation week 28**	**[Immunisation week 52]**
Live vaccines	BCG vaccination / re-vaccination[1,2,3]	Yellow fever (YF-17D)<br>Oral typhoid (Ty21a)			
Non-live vaccines		HPV prime	HPV boost for girls aged ≥14 years[4,5]	HPV boost and Tetanus/ diphtheria boost	Tetanus/ diphtheria boost[5,6]

1. All participants in the urban (Entebbe Mother and Baby Study; EMaBS) cohort received BCG at birth, so within EMaBS this will be revaccination; prior BCG status may vary for the rural cohorts (data on history and documentation of prior BCG, and presence of a BCG scar, will be documented although these approaches have limitations for determining BCG status)
2. EMaBS participants will be randomised to receive BCG "pre-immunisation" or not as part of Trial C
3. All participants in rural cohorts will receive BCG (Trials A and B)
4. The National EPI programme recommends three doses of HPV vaccine for older girls
5. These doses will be given to comply with guidelines but outcomes specifically relating to these doses will not be assessed
6. Priming by immunisation in infancy is assumed

For peer review only

Outcomes

Primary outcomes, assessed in all participants, will be:

i. **BCG:** *BCG-specific IFN-γ ELISpot response* eight weeks post BCG immunisation: this response is associated with decreased risk of tuberculosis disease post-immunisation in infants.[60]
ii. **YF-17D:** neutralising antibody titres (plaque-reduction neutralisation test) four weeks post YF immunisation.
iii. **Ty21a:** *Salmonella typhi* lipopolysaccharide (LPS)-specific immunoglobulin (Ig)G concentration four weeks post Ty21a immunisation.
iv. **HPV:** IgG specific for L1-proteins of HPV-16/18 four weeks post HPV priming immunisation.
v. **Td:** tetanus and diphtheria toxoid-specific IgG concentration four weeks post Td immunisation.

Secondary outcomes, assessed in all participants, will further investigate estimates of protective immunity (for vaccines where these are available) and dynamics of the vaccine responses, as well as the impact of interventions on parasite clearance.

i. **Protective immunity.** Proportions with protective neutralising antibody (YF); protective IgG levels (TT);[61] seroconversion rates (Ty21a) four weeks post immunisation.
ii. **Response waning.** Primary outcome measures (all vaccines) repeated at week 52, and area-under-the curve analyses. Parasites may accelerate,[58] and interventions delay, waning.
iii. **Priming versus boosting.** Effects on priming versus boosting will be examined for HPV, comparing outcomes four weeks after the first and second vaccine doses.

Our sample collection will offer opportunities for an array of exploratory immunological evaluations, focusing on vaccine antigen specific outcomes. Exploratory assessments will provide detail on immune response characteristics over the study time-course, and on the role of immunological profiles and trans-kingdom effects in mediating modulation of vaccine-specific responses.

Sample size

Our sample size estimates focus on primary outcomes for the main comparisons for objectives i, ii and iv.

Based on literature,[5 60 62] we anticipate standard deviations (SDs) of primary outcome measures lying between 0.3 and 0.6 $\log_{10}$; responses in rural, high-parasite settings 0.3-0.4 $\log_{10}$ smaller than in the urban setting,[63 64] and effective treatment restoring responses by approximately 0.2 $\log_{10}$.[63] We therefore power our study to detect differences of this magnitude (0.2 $\log_{10}$) or smaller. We assume

For peer review only

S. mansoni prevalence of >80% in the high-schistosomiasis setting[47] and malaria infection prevalence of >60% in the high-malaria setting.

Our planned sample sizes are as follows:

High-schistosomiasis setting (objective i, Trial A): 480 (240 quarterly PZQ, 240 annual PZQ); of whom we anticipate 384 will be *S. mansoni* infected, giving 192 participants in each trial arm with *S. mansoni* infection at baseline.

High-malaria setting (objective i, Trial B): 640 (320 DP, 320 placebo) of whom we anticipate 384 will be malaria infected, giving 192 participants with malaria in each trial arm at baseline.

Urban setting (objective ii, Trial C): 300 EMaBS participants (150 BCG "pre-immunisation", 150 no BCG immunisation).

Urban vs rural and urban vs island comparisons (objective iv): 150 urban EMaBS, 240 rural high-schistosomiasis control group participants and 320 rural high-malaria control group participants will be included. Allowing 20% loss to follow-up in rural cohorts and 10% in the EMaBS cohort, this will give >80% power to detect a difference of $0.14\log_{10}$ or more in vaccine responses in urban compared to each rural setting at 5% significance level assuming vaccine response standard deviation of $0.4\log_{10}$.

Table 2 shows power for objective i, ii and iv primary comparisons, for 5% significance level and assuming 10% loss to follow-up in Trial C (EMaBS) participants and 20% loss to follow-up in rural cohorts. Sample size considerations for additional analyses are detailed in the protocol papers for the individual trials.

Table 2. Power estimates for objectives i, ii and iv (5% significance level)

	**$\log_{10}$ difference**						
**Standard deviation ($\log_{10}$)**	**0.08**	**0.10**	**0.12**	**0.14**	**0.16**	**0.18**	**0.20**
Objective i: 192 high-intensity vs 192 low-intensity (infected only)							
0.3	65%	83%	94%	98%	>99%	>99%	>99%
0.4	42%	59%	75%	87%	94%	98%	99%
0.5	29%	42%	56%	69%	80%	88%	94%
0.6	21%	31%	42%	53%	65%	75%	83%
Objective ii: 150 BCG "pre-immunisation" vs 150 no BCG vaccination							
0.3	59%	78%	91%	97%	99%	>99%	>99%
0.4	37%	53%	69%	82%	91%	96%	98%
0.5	26%	37%	50%	63%	75%	84%	91%
0.6	19%	28%	37%	48%	59%	69%	78%
Objective iv: 240 rural vs 150 urban[1]							
0.3	66%	84%	94%	99%	>99%	>99%	>99%
0.4	43%	60%	76%	87%	94%	98%	99%
0.5	30%	43%	57%	80%	81%	89%	94%
0.6	22%	32%	43%	54%	66%	76%	84%

Cells highlighted in grey correspond to >80% power.

For peer review only

[1]Numbers shown for rural high-schistosomiasis versus urban setting. Power will be greater for rural high-malaria vs urban setting

Approach to trial objectives

Approaches to objective i (trials A and B) and objective ii (trial C) are detailed in the focused papers for these trials (published in this issue). Here we present approaches to objectives iii, iv, v and vi.

Objective iii. Life-course exposures that influence vaccine responses in adolescence (Trial C)

Data collected in the EMaBS over the participants' life course (and as part of this protocol) will be used in regression analyses to investigate associations between infection exposure *in utero*, infancy, childhood, and adolescence on vaccine responses. Socio-demographic variables will be considered as potential confounders. We will include the following important variables: age, sex, sociodemographic variables, BCG strain received at birth (and other vaccines received), helminth-related exposures, malaria and documented illness events.

Regression analyses will be used to evaluate associations between infectionss and outcomes, with adjustment for confounders. We shall also investigate whether multiple infection exposures combine multiplicatively in their effect, and test for interaction.

Genetic factors will also be considered: genetic data is already available for EMaBS based on earlier approvals for work on genetic polymorphisms and vaccine responses in infancy. It will be of interest to determine whether genetic factors also have a strong influence on adolescents' responses.

Objective iv. Urban-rural comparisons in vaccine response

We hypothesise that environmental (especially parasite) exposures are key drivers of population differences in vaccine response. Hence we predict differences between urban and rural settings within Uganda (with urban vaccine responses stronger, as observed in Gabon[8 9] and Senegal[64]) and that these differences will be related to parasite exposure.

The key exposure for this objective is 'setting'. We will compare outcomes between urban EMaBS and (i) rural high-schistosomiasis and (ii) rural high-malaria participants. For these comparisons we shall include in the analysis only the urban EMaBS participants who were randomised to receive BCG "pre-immunisation," such that their immunisation schedule is identical to that in the other two settings. We will include only control groups from the rural high-schistosomiasis and high-malaria settings, since these will have received minimal anti-parasite treatment (**Figures 3** and **4**). In the primary analysis we will adjust for age and sex, but not for factors likely to be on the causal pathway between setting and vaccine response.

For peer review only

For peer review only - http://bmjopen.bmj.com/site/about/guidelines.xhtml

Although our settings are purposely chosen based on parasite prevalence, there will be overlap in this, and other, exposures. Therefore we will undertake exploratory analyses using causal mediation modelling, a statistical approach which aims to identify factors that mediate an observed association [65] (here, between setting and vaccine response). We will focus on current *S. mansoni* and *P. falciparum* infection, and prior exposure (assessed by anti-schistosome and anti-malaria antibody), as key potential mediators of interest. Explanatory factors for urban versus rural setting, and for vaccine responses, will be included in the causal diagram and adjusted for in analyses. Although nutrition may impact vaccine responses,[66] we expect this to be less important in healthy adolescents than might be the case in young children; however, data on anthropometric parameters and diet will be collected and adjusted for.

One limitation is that observational analyses of parasite effects are beset with potential unmeasured confounding. Results will be interpreted cautiously and Objective i (Trials A and B) will address causality rigorously. The sample archive will allow future investigation of additional potential mediators. Another potential limitation is that in Uganda, rural to urban migration for schooling is common. However, in the EMaBS birth cohort we have data on residence. Urban to rural migration for schooling is relatively unlikely but will be documented.

With Objective iv we expect to confirm differences in vaccine responses between settings; and to obtain insights into environmental factors that mediate this. By addressing the portfolio of vaccines and array of outcomes presented above, we will identify categories of vaccine most affected; we will distinguish effects on waning and (for HPV) on priming versus boosting. Vaccine response data from urban EMaBS adolescents will provide a reference, suggesting the extent to which changes in infection exposure and lifestyle are likely to influence responses to vaccines (and to infectious diseases) as Africa's urbanisation advances.

Objective v. The role of "trans-Kingdom" interactions in determining vaccine responses

We will address the hypothesis that parasites also impact vaccine responses through "trans-kingdom" effects, mediated by other components of the host ecosystem. We propose that herpesvirus activation and microbial translocation are likely to be determinants of vaccine response, and they are driven by parasitic infections. Samples from selected participants from each setting and study arm will be examined. To optimise the precision of our comparisons these will be selected among those (for the high-schistosomiasis and high-malaria settings) who had the parasitic infection of interest at baseline (and, if possible, no other parasitic infection detected) and complied with the intended treatment. To test our hypothesis, we will measure markers of viral activation and microbial translocation in plasma/serum and/or stool. Initial analyses (**Figure 5**) will investigate

For peer review only

associations between these markers and vaccine responses (arrow F); and between settings (arrow D) or parasite infection and treatment (arrow E) and viral activation or microbial translocation. Objective vi will link herpesvirus activation, microbial translocation and parasitic infections to immune activation and regulation.

Objective vi. Pre-immunisation immunological parameters

There is strong evidence that pre-immunisation immunological status impacts vaccine responses[5,60] but the factors that determine this status have not been identified. We hypothesise that the environmental, parasitic, viral and microbial exposures addressed in Objectives i-v are key (**Figure 5**). We aim to investigate this by identifying immunological parameters that specifically link the distal exposures to vaccine response. These include the circulating cytokine and chemokine milieu, innate cell responses (which govern adaptive responses), and frequencies and phenotypes of both innate and adaptive cells. Our principal focus will be on pre-immunisation measurements although, in selected groups of most interest, it will also be possible to examine samples obtained post-immunisation to identify which biomarkers or cell populations change.

Initial analyses will confirm hypothesised, and identify new, pre-immunisation immunological parameters associated with responses to vaccines in our portfolio (**Figure 5**, arrow J). If we observe associations between rural vs urban location (arrow B) or parasites and their treatment (arrow C) and vaccines responses, we will explore whether these effects are mediated by viral and microbial translocation variables and pre-immunisation immune profiles using causal mediation analyses.[65]

Operational considerations

A Programme Steering Committee has been set up to guide progress across all projects. A data and safety monitoring board (DSMB) has also been appointed to provide real-time safety oversight. Details of these and other operational activities can be found in Supplementary information.

Ethical and regulatory considerations

Ethical and regulatory approval has been granted from the Research Ethics Committees of Uganda Virus Research Institute (UVRI REC) and the London School of Hygiene and Tropical Medicine (LSHTM), the Uganda National Council for Science and Technology (UNCST) and the Uganda National Drug Authority (NDA); details are given in each Trial paper. Given the importance of the data and sample archive as a resource for mechanistic studies on the determinants of vaccine responses, assent and consent processes will include storage of samples for future and genetic studies, and anonymised data and sample sharing. Any protocol amendments will be submitted to ethics committees and regulatory bodies for approval before implementation.

For peer review only

Patient and public involvement

Concepts involved in this work have been discussed with colleagues at the Vector Control Division and Expanded Programme on Immunisation in the Ministry of Health (Uganda) and with relevant District Councils, community leaders and Village Health Teams. We also have held meetings to explain the proposed work to teachers, parents, participants and village members, and to address their questions. Study findings will be shared with these stakeholders.

Data management and analysis

Socio-demographic information and clinical and laboratory measurements will be recorded and managed using REDCap (Research Electronic Data Capture) tools,[67 68] with paper-based forms as back-up. All data will be recorded under a unique study ID number. When paper forms must be used, data will be entered in a study-specific database, with standard checks for discrepancies. All data for analysis will be anonymised and stored on a secure and password-protected server, with access limited to essential research personnel. Anonymised participant level datasets generated will be available for sharing upon request.

For peer review only

For peer review only - http://bmjopen.bmj.com/site/about/guidelines.xhtml

Discussion

This will be the first well-powered set of studies to investigate effects of schistosomiasis and malaria treatment, and of BCG re-vaccination, on vaccine responses in adolescents. The results will add to understanding of population differences in vaccine responses and of interventions that may enhance them. The sample archives developed will provide a major asset for exploration of new leads arising from this hypothesis-driven work, or for an alternative, “systems biology” approach investigating (for example) transcriptome, microbiome and virome.

Our focus is on the immunological effects of infection exposure in human participants. By understanding these effects, we aim to inform and promote vaccine design tailored to the challenging environment of low-income countries, and to inform the development of public health strategies (such as tailored immunisation regimens and combined parasite-control/immunisation interventions) that will optimise vaccine implementation in parasite-endemic settings.

Our strong immunoepidemiological design and nested immunological studies will address specific hypotheses regarding pathways of effects. Population immunology is useful for translation of findings to and from basic (especially animal) studies to human health. Our randomised design will determine causal, and reversible, effects of parasitic infections. Substantial sample sizes are needed because immune responses are highly variable in human populations.

We have several reasons for studying vaccine responses particularly among adolescents. In this study setting, they bear a heavy parasite burden.[69] As well, this age-group is a target group for vaccines against tuberculosis and sexually transmitted infections (currently human papilloma virus [HPV] – in future, it is hoped, for vaccines against HIV) and for booster immunisations. Also, they enter a period of increased risk of pulmonary tuberculosis after the relatively low-risk period of mid-childhood, and are thus a target group for improved vaccines for tuberculosis.

Study timeline

POPVAC A began recruiting in July 2019. Intervention will be up to 12 months, with completion of the project scheduled for September 2020. POPVAC B is scheduled begin recruiting in February 2021. Intervention will be up to 12 months, with completion of the project scheduled for April 2022. POPVAC C is scheduled to begin recruiting in May 2020. Intervention will be up to 12 months, with completion of the project scheduled for April 2022.

For peer review only

For peer review only - http://bmjopen.bmj.com/site/about/guidelines.xhtml

Competing interests

Alison Elliott reports a grant from the Medical research Council, UK (POPVAC programme funding). The rest of the authors declare that they have no conflicts of interest.

Author contributions

AME conceived the study. AME, GN, ELW, AN, SC, LZ, JN, AW, PK and HL contributed to study design. LZ, GO, CN, CZ, and FA are site clinicians/nurses/clinical laboratory technicians providing valuable input on clinical considerations of the intervention. RK heads the team of field workers handling the organisational integration of the intervention. AN and ELW are involved in organisation of the databases, trial randomization, treatment allocation and drawing up of analytical plans. GN, LZ, AN, SC, ELW and AME drafted the manuscript. All authors reviewed the manuscript, contributed to it and approved the final version.

Acknowledgements

We thank the Uganda National Expanded Programme for Immunisation, the Vector Control Division of the Ministry of Health, the Ministry of Education, Jinja district local government, Wakiso district local government and Entebbe hospital for their support. We thank members of the POPVAC programme steering committee (chaired by Prof. Richard Hayes) and the Data and Safety Monitoring Board (Dr David Meya, Prof. Andrew Prendergast and Dr Elizabeth George).

Funding

The POPVAC programme of work is supported by the Medical Research Council of the United Kingdom (grant number MR/R02118X/1). SC and JN are supported in part by the Makerere University – Uganda Virus Research Institute Centre of Excellence for Infection and Immunity Research and Training (MUII-plus). MUII-plus is funded under the DELTAS Africa Initiative. The DELTAS Africa Initiative is an independent funding scheme of the African Academy of Sciences (AAS), Alliance for Accelerating Excellence in Science in Africa (AESA) and supported by the New Partnership for Africa's Development Planning and Coordinating Agency (NEPAD Agency) with funding from the Wellcome Trust (grant 107743) and the UK Government. The MRC/UVRI and LSHTM Uganda Research Unit is jointly funded by the UK Medical Research Council (MRC) and the UK Department for International Development (DFID) under the MRC/DFID Concordat agreement and is also part of the EDCTP2 programme supported by the European Union.

The study sponsor (London School of Hygiene and Tropical Medicine) and funders had no role in study design; collection, management, analysis, and interpretation of data; writing of the protocol; and the decision to submit the protocol for publication.

For peer review only

POPVAC trial team

Principal investigator*:* Alison Elliott; ***Project leader***: Ludoviko Zirimenya; ***laboratory staff****:* Gyaviira Nkurunungi, Stephen Cose, Rebecca Amongin, Beatrice Nassanga, Jacent Nassuuna, Irene Nambuya, Prossy Kabuubi, Emmanuel Niwagaba, Gloria Oduru, Grace Kabami; ***statisticians and data managers****:* Emily Webb, Agnes Natukunda, Helen Akurut, Alex Mutebe; ***clinicians****:* Anne Wajja, Milly Namutebi, Christopher Zziwa; ***nurses****:* Caroline Onen, Esther Nakazibwe, Josephine Tumusiime, Caroline Ninsiima, Susan Amongi, Florence Akello; ***internal monitor****:* Mirriam Akello; ***field workers****:* Robert Kizindo, Moses Sewankambo, Denis Nsubuga, Samuel Kiwanuka, Fred Kiwudhu; ***boatman****:* David Abiriga; ***administrative management****:* Moses Kizza, Samsi Nansukusa; ***internal and external collaborators:*** Pontiano Kaleebu, Hermelijn Smits, Maria Yazdanbakhsh, Govert van Dam, Paul Corstjens, Sarah Staedke, Henry Luzze, James Kaweesa, Edridah Tukahebwa, Elly Tumushabe, Moses Muwanga

For peer review only

For peer review only - http://bmjopen.bmj.com/site/about/guidelines.xhtml

References

1. Koff WC, Burton DR, Johnson PR, et al. Accelerating next-generation vaccine development for global disease prevention. *Science* 2013;340(6136):1232910. doi: 10.1126/science.1232910

2. Taniuchi M, Platts-Mills JA, Begum S, et al. Impact of enterovirus and other enteric pathogens on oral polio and rotavirus vaccine performance in Bangladeshi infants. *Vaccine* 2016;34(27):3068-75. doi: 10.1016/j.vaccine.2016.04.080 [published Online First: 2016/05/08]

3. Fine PE. Variation in protection by BCG: implications of and for heterologous immunity. *Lancet* 1995;346(8986):1339-45.

4. Black GF, Weir RE, Floyd S, et al. BCG-induced increase in interferon-gamma response to mycobacterial antigens and efficacy of BCG vaccination in Malawi and the UK: two randomised controlled studies. *Lancet* 2002;359(9315):1393-401. doi: 10.1016/S0140-6736(02)08353-8

5. Muyanja E, Ssemaganda A, Ngauv P, et al. Immune activation alters cellular and humoral responses to yellow fever 17D vaccine. *The Journal of clinical investigation* 2014;124(7):3147-58. doi: 10.1172/jci75429 [published Online First: 2014/06/10]

6. Barreto ML, Pilger D, Pereira SM, et al. Causes of variation in BCG vaccine efficacy: examining evidence from the BCG REVAC cluster randomized trial to explore the masking and the blocking hypotheses. *Vaccine* 2014;32(30):3759-64. doi: 10.1016/j.vaccine.2014.05.042

7. Barreto ML, Pereira SM, Pilger D, et al. Evidence of an effect of BCG revaccination on incidence of tuberculosis in school-aged children in Brazil: second report of the BCG-REVAC cluster-randomised trial. *Vaccine* 2011;29(31):4875-7. doi: 10.1016/j.vaccine.2011.05.023

8. van Riet E, Adegnika AA, Retra K, et al. Cellular and humoral responses to influenza in gabonese children living in rural and semi-urban areas. *The Journal of infectious diseases* 2007;196(11):1671-8. doi: 10.1086/522010 [published Online First: 2007/11/17]

9. van Riet E, Retra K, Adegnika AA, et al. Cellular and humoral responses to tetanus vaccination in Gabonese children. *Vaccine* 2008;26(29-30):3690-5. doi: 10.1016/j.vaccine.2008.04.067 [published Online First: 2008/06/10]

10. Tanner R, Kakalacheva K, Miller E, et al. Serum indoleamine 2,3-dioxygenase activity is associated with reduced immunogenicity following vaccination with MVA85A. *BMC infectious diseases* 2014;14:660. doi: 10.1186/s12879-014-0660-7 [published Online First: 2014/12/04]

11. Sissoko MS, Healy SA, Katile A, et al. Safety and efficacy of PfSPZ Vaccine against Plasmodium falciparum via direct venous inoculation in healthy malaria-exposed adults in Mali: a randomised, double-blind phase 1

For peer review only

trial. *The Lancet Infectious diseases* 2017;17(5):498-509. doi: 10.1016/s1473-3099(17)30104-4 [published Online First: 2017/02/22]

12. Venkatraman N, Ndiaye BP, Bowyer G, et al. Safety and Immunogenicity of a Heterologous Prime-Boost Ebola Virus Vaccine Regimen in Healthy Adults in the United Kingdom and Senegal. *The Journal of infectious diseases* 2019;219(8):1187-97. doi: 10.1093/infdis/jiy639

13. Colley DG, Bustinduy AL, Secor WE, et al. Human schistosomiasis. *Lancet* 2014;383(9936):2253-64. doi: 10.1016/s0140-6736(13)61949-2 [published Online First: 2014/04/05]

14. Pullan RL, Smith JL, Jasrasaria R, et al. Global numbers of infection and disease burden of soil transmitted helminth infections in 2010. *Parasites & vectors* 2014;7:37. doi: 10.1186/1756-3305-7-37 [published Online First: 2014/01/23]

15. WHO. World Malaria Report. 2016

16. Crawley J, Sismanidis C, Goodman T, et al. Effect of intermittent preventive treatment for malaria during infancy on serological responses to measles and other vaccines used in the Expanded Programme on Immunization: results from five randomised controlled trials. *Lancet* 2012;380(9846):1001-10. doi: 10.1016/s0140-6736(12)60775-2 [published Online First: 2012/08/02]

17. Brown J, Baisley K, Kavishe B, et al. Impact of malaria and helminth infections on immunogenicity of the human papillomavirus-16/18 AS04-adjuvanted vaccine in Tanzania. *Vaccine* 2014;32(5):611-7. doi: 10.1016/j.vaccine.2013.11.061

18. Cunnington AJ, Riley EM. Suppression of vaccine responses by malaria: insignificant or overlooked? *Expert review of vaccines* 2010;9(4):409-29. doi: 10.1586/erv.10.16 [published Online First: 2010/04/08]

19. Bentwich Z, Kalinkovich A, Weisman Z, et al. Can eradication of helminthic infections change the face of AIDS and tuberculosis? *Immunol Today* 1999;20(11):485-7.

20. Elias D, Akuffo H, Pawlowski A, et al. Schistosoma mansoni infection reduces the protective efficacy of BCG vaccination against virulent Mycobacterium tuberculosis. *Vaccine* 2005;23(11):1326-34. doi: https://doi.org/10.1016/j.vaccine.2004.09.038

21. Laranjeiras RF, Brant LC, Lima AC, et al. Reduced protective effect of Plasmodium berghei immunization by concurrent Schistosoma mansoni infection. *Mem Inst Oswaldo Cruz* 2008;103(7):674-7. doi: 10.1590/s0074-02762008000700008 [published Online First: 2008/12/06]

22. Su Z, Segura M, Stevenson MM. Reduced protective efficacy of a blood-stage malaria vaccine by concurrent nematode infection. *Infection and immunity* 2006;74(4):2138-44. doi: 10.1128/iai.74.4.2138-2144.2006 [published Online First: 2006/03/23]

For peer review only

23. Sabin EA, Araujo MI, Carvalho EM, et al. Impairment of tetanus toxoid-specific Th1-like immune responses in humans infected with Schistosoma mansoni. *The Journal of infectious diseases* 1996;173(1):269-72. [published Online First: 1996/01/01]

24. Esen M, Mordmuller B, de Salazar PM, et al. Reduced antibody responses against Plasmodium falciparum vaccine candidate antigens in the presence of Trichuris trichiura. *Vaccine* 2012;30(52):7621-4. doi: 10.1016/j.vaccine.2012.10.026 [published Online First: 2012/10/23]

25. Kilian HD, Nielsen G. Cell-mediated and humoral immune response to tetanus vaccinations in onchocerciasis patients. *Trop Med Parasitol* 1989;40(3):285-91. [published Online First: 1989/09/01]

26. Sanya RE, Nkurunungi G, Andia Biraro I, et al. A life without worms. *Transactions of the Royal Society of Tropical Medicine and Hygiene* 2017:1-9. doi: 10.1093/trstmh/trx010 [published Online First: 2017/03/25]

27. Netea MG, Joosten LAB, Latz E, et al. Trained immunity: A program of innate immune memory in health and disease. *Science (New York, NY)* 2016;352(6284):aaf1098-aaf98. doi: 10.1126/science.aaf1098 [published Online First: 2016/04/21]

28. Blok BA, Arts RJ, van Crevel R, et al. Trained innate immunity as underlying mechanism for the long-term, nonspecific effects of vaccines. *J Leukoc Biol* 2015;98(3):347-56. doi: 10.1189/jlb.5RI0315-096R

29. Leentjens J, Kox M, Stokman R, et al. BCG Vaccination Enhances the Immunogenicity of Subsequent Influenza Vaccination in Healthy Volunteers: A Randomized, Placebo-Controlled Pilot Study. *The Journal of infectious diseases* 2015;212(12):1930-8. doi: 10.1093/infdis/jiv332

30. Pfeiffer JK, Virgin HW. Viral immunity. Transkingdom control of viral infection and immunity in the mammalian intestine. *Science* 2016;351(6270) doi: 10.1126/science.aad5872

31. Reese TA, Wakeman BS, Choi HS, et al. Helminth infection reactivates latent gamma-herpesvirus via cytokine competition at a viral promoter. *Science* 2014;345(6196):573-7. doi: 10.1126/science.1254517 [published Online First: 2014/06/28]

32. McFarlane AJ, McSorley HJ, Davidson DJ, et al. Enteric helminth-induced type I interferon signaling protects against pulmonary virus infection through interaction with the microbiota. *The Journal of allergy and clinical immunology* 2017 doi: 10.1016/j.jaci.2017.01.016 [published Online First: 2017/02/16]

33. Wakeham K, Johnston WT, Nalwoga A, et al. Trends in Kaposi's sarcoma-associated Herpesvirus antibodies prior to the development of HIV-associated Kaposi's sarcoma: a nested case-control study. *Int J Cancer* 2015;136(12):2822-30. doi: 10.1002/ijc.29329

For peer review only

For peer review only - http://bmjopen.bmj.com/site/about/guidelines.xhtml

539 34. Wakeham K, Webb EL, Sebina I, et al. Parasite infection is associated with Kaposi's sarcoma associated
540 herpesvirus (KSHV) in Ugandan women. *Infectious agents and cancer* 2011;6(1):15. doi: 10.1186/1750-9378-
541 6-15

542 35. Wakeham K, Webb EL, Sebina I, et al. Risk factors for seropositivity to Kaposi sarcoma-associated
543 herpesvirus among children in Uganda. *Journal of acquired immune deficiency syndromes* 2013;63(2):228-33.
544 doi: 10.1097/QAI.0b013e31828a7056

545 36. Moormann AM, Bailey JA. Malaria - how this parasitic infection aids and abets EBV-associated Burkitt
546 lymphomagenesis. *Current opinion in virology* 2016;20:78-84. doi: 10.1016/j.coviro.2016.09.006 [published
547 Online First: 2016/10/01]

548 37. Frasca D, Blomberg BB. Aging, cytomegalovirus (CMV) and influenza vaccine responses. *Human vaccines &*
549 *immunotherapeutics* 2016;12(3):682-90. doi: 10.1080/21645515.2015.1105413 [published Online First:
550 2015/11/21]

551 38. Onguru D, Liang Y, Griffith Q, et al. Human schistosomiasis is associated with endotoxemia and Toll-like
552 receptor 2- and 4-bearing B cells. *The American journal of tropical medicine and hygiene* 2011;84(2):321-4.
553 doi: 10.4269/ajtmh.2011.10-0397 [published Online First: 2011/02/05]

554 39. George PJ, Anuradha R, Kumar NP, et al. Evidence of microbial translocation associated with perturbations
555 in T cell and antigen-presenting cell homeostasis in hookworm infections. *PLoS neglected tropical diseases*
556 2012;6(10):e1830. doi: 10.1371/journal.pntd.0001830 [published Online First: 2012/10/12]

557 40. Rajamanickam A, Munisankar S, Bhootra Y, et al. Microbial Translocation Associated with an Acute-Phase
558 Response and Elevations in MMP-1, HO-1, and Proinflammatory Cytokines in Strongyloides stercoralis
559 Infection. *Infection and immunity* 2017;85(1) doi: 10.1128/iai.00772-16 [published Online First: 2016/11/09]

560 41. Mbow M, de Jong SE, Meurs L, et al. Changes in immunological profile as a function of urbanization and
561 lifestyle. *Immunology* 2014;143(4):569-77. doi: 10.1111/imm.12335

562 42. Smolen KK, Ruck CE, Fortuno ES, 3rd, et al. Pattern recognition receptor-mediated cytokine response in
563 infants across 4 continents. *The Journal of allergy and clinical immunology* 2014;133(3):818-26 e4. doi:
564 10.1016/j.jaci.2013.09.038

565 43. Idaghdour Y, Storey JD, Jadallah SJ, et al. A genome-wide gene expression signature of environmental
566 geography in leukocytes of Moroccan Amazighs. *PLoS genetics* 2008;4(4):e1000052. doi:
567 10.1371/journal.pgen.1000052

568 44. Amoah AS, Obeng BB, May L, et al. Urban-rural differences in the gene expression profiles of Ghanaian
569 children. *Genes and immunity* 2014;15(5):313-9. doi: 10.1038/gene.2014.21

For peer review only

45. Labuda LA, de Jong SE, Meurs L, et al. Differences in innate cytokine responses between European and African children. *PloS one* 2014;9(4):e95241. doi: 10.1371/journal.pone.0095241 [published Online First: 2014/04/20]

46. Chan AW, Tetzlaff JM, Altman DG, et al. SPIRIT 2013 statement: defining standard protocol items for clinical trials. *Ann Intern Med* 2013;158(3):200-7. doi: 10.7326/0003-4819-158-3-201302050-00583 [published Online First: 2013/01/09]

47. Sanya RE, Nkurunungi G, Hoek Spaans R, et al. The Impact of Intensive Versus Standard Anthelminthic Treatment on Allergy-related Outcomes, Helminth Infection Intensity, and Helminth-related Morbidity in Lake Victoria Fishing Communities, Uganda: Results From the LaVIISWA Cluster-randomized Trial. *Clinical Infectious Diseases* 2018:ciy761-ciy61. doi: 10.1093/cid/ciy761

48. Tukahebwa EM, Magnussen P, Madsen H, et al. A very high infection intensity of Schistosoma mansoni in a Ugandan Lake Victoria Fishing Community is required for association with highly prevalent organ related morbidity. *PLoS neglected tropical diseases* 2013;7(7):e2268. doi: 10.1371/journal.pntd.0002268 [published Online First: 2013/08/13]

49. Kakuru A, Jagannathan P, Muhindo MK, et al. Dihydroartemisinin–Piperaquine for the Prevention of Malaria in Pregnancy. *New England Journal of Medicine* 2016;374(10):928-39. doi: 10.1056/NEJMoa1509150

50. Nanteza B, Galukande M, Aceng J, et al. The burden of tetanus in Uganda. *SpringerPlus* 2016;5(1):705. doi: 10.1186/s40064-016-2309-z [published Online First: 2016/06/29]

51. Staedke SG, Maiteki-Sebuguzi C, Rehman AM, et al. Assessment of community-level effects of intermittent preventive treatment for malaria in schoolchildren in Jinja, Uganda (START-IPT trial): a cluster-randomised trial. *The Lancet Global Health* 2018;6(6):e668-e79. doi: https://doi.org/10.1016/S2214-109X(18)30126-8

52. Nkurunungi G, Lubyayi L, Versteeg SA, et al. Do helminth infections underpin urban-rural differences in risk factors for allergy-related outcomes? *Clinical and experimental allergy : journal of the British Society for Allergy and Clinical Immunology* 2019 doi: 10.1111/cea.13335 [published Online First: 2019/01/12]

53. Namara B, Nash S, Lule SA, et al. Effects of treating helminths during pregnancy and early childhood on risk of allergy-related outcomes: Follow-up of a randomized controlled trial. *Pediatric allergy and immunology : official publication of the European Society of Pediatric Allergy and Immunology* 2017;28(8):784-92. doi: 10.1111/pai.12804 [published Online First: 2017/09/12]

54. Webb EL, Mawa PA, Ndibazza J, et al. Effect of single-dose anthelmintic treatment during pregnancy on an infant's response to immunisation and on susceptibility to infectious diseases in infancy: a randomised, double-blind, placebo-controlled trial. *Lancet* 2011;377(9759):52-62. doi: 10.1016/S0140-6736(10)61457-2

For peer review only

55. Akondy RS, Johnson PL, Nakaya HI, et al. Initial viral load determines the magnitude of the human CD8 T cell response to yellow fever vaccination. *Proceedings of the National Academy of Sciences of the United States of America* 2015;112(10):3050-5. doi: 10.1073/pnas.1500475112 [published Online First: 2015/02/26]

56. Elliott AM, Mawa PA, Webb EL, et al. Effects of maternal and infant co-infections, and of maternal immunisation, on the infant response to BCG and tetanus immunisation. *Vaccine* 2010;29(2):247-55. doi: 10.1016/j.vaccine.2010.10.047

57. Naylor C, Lu M, Haque R, et al. Environmental Enteropathy, Oral Vaccine Failure and Growth Faltering in Infants in Bangladesh. *EBioMedicine* 2015;2(11):1759-66. doi: 10.1016/j.ebiom.2015.09.036 [published Online First: 2016/02/13]

58. Riner DK, Ndombi EM, Carter JM, et al. Schistosoma mansoni Infection Can Jeopardize the Duration of Protective Levels of Antibody Responses to Immunizations against Hepatitis B and Tetanus Toxoid. *PLoS neglected tropical diseases* 2016;10(12):e0005180. doi: 10.1371/journal.pntd.0005180

59. Wajja A, Kizito D, Nassanga B, et al. The effect of current Schistosoma mansoni infection on the immunogenicity of a candidate TB vaccine, MVA85A, in BCG-vaccinated adolescents: an open-label trial. *PLoS neglected tropical diseases* (in press)

60. Fletcher HA, Snowden MA, Landry B, et al. T-cell activation is an immune correlate of risk in BCG vaccinated infants. *Nat Commun* 2016;7:11290. doi: 10.1038/ncomms11290

61. Plotkin SA. Correlates of protection induced by vaccination. *Clinical and vaccine immunology : CVI* 2010;17(7):1055-65. doi: 10.1128/cvi.00131-10 [published Online First: 2010/05/14]

62. Safaeian M, Porras C, Pan Y, et al. Durable antibody responses following one dose of the bivalent human papillomavirus L1 virus-like particle vaccine in the Costa Rica Vaccine Trial. *Cancer prevention research (Philadelphia, Pa)* 2013;6(11):1242-50. doi: 10.1158/1940-6207.capr-13-0203 [published Online First: 2013/11/06]

63. Tweyongyere R, Nassanga BR, Muhwezi A, et al. Effect of Schistosoma mansoni infection and its treatment on antibody responses to measles catch-up immunisation in pre-school children: A randomised trial. *PLoS neglected tropical diseases* 2019;13(2):e0007157. doi: 10.1371/journal.pntd.0007157 [published Online First: 2019/02/15]

64. Venkatraman N, Ndiaye BP, Bowyer G, et al. Safety and Immunogenicity of a Heterologous Prime-Boost Ebola Virus Vaccine Regimen in Healthy Adults in the United Kingdom and Senegal. *The Journal of infectious diseases* 2018;219(8):1187-97. doi: 10.1093/infdis/jiy639

For peer review only

For peer review only - http://bmjopen.bmj.com/site/about/guidelines.xhtml

65. Imai K, Keele L, Yamamoto T. Identification, inference and sensitivity analysis for causal mediation effects. *Statistical Science* 2010;25(1):51-71.

66. Prendergast AJ. Malnutrition and vaccination in developing countries. *Philosophical transactions of the Royal Society of London Series B, Biological sciences* 2015;370(1671) doi: 10.1098/rstb.2014.0141 [published Online First: 2015/05/13]

67. Harris PA, Taylor R, Minor BL, et al. The REDCap Consortium: Building an International Community of Software Platform Partners. *Journal of biomedical informatics* 2019:103208. doi: 10.1016/j.jbi.2019.103208 [published Online First: 2019/05/13]

68. Harris PA, Taylor R, Thielke R, et al. Research electronic data capture (REDCap)--a metadata-driven methodology and workflow process for providing translational research informatics support. *Journal of biomedical informatics* 2009;42(2):377-81. doi: 10.1016/j.jbi.2008.08.010 [published Online First: 2008/10/22]

69. Webb EL, Nampijja M, Kaweesa J, et al. Helminths are positively associated with atopy and wheeze in Ugandan fishing communities: results from a cross-sectional survey. *Allergy* 2016 doi: 10.1111/all.12867

FIGURE LEGENDS

Figure 1. Hypothesised pathways to population differences in vaccine responses

Figure 2. Study sites

Figure 3. Overall programme design and sample sizes

Figure 4. Outline of immunisations and interventions

[1]Primary endpoints will be at eight weeks post BCG and four weeks post Yellow fever (YF-17D), oral typhoid (Ty21a), Human Papilloma Virus (HPV) and Tetanus/diptheria (Td) vaccination.

[2]Primary endpoint for responses to Td given at 28 weeks.

Figure 5. Synthesised analysis of study objectives

Red arrows represent our principal hypotheses.

Arrows A, B and C are considered in objectives i and iv;

Arrows D, E and F in objective v;

Arrows G, H, I and J, in objective vi; as well as the fully synthesised analysis

For peer review only - http://bmjopen.bmj.com/site/about/guidelines.xhtml

For peer review only

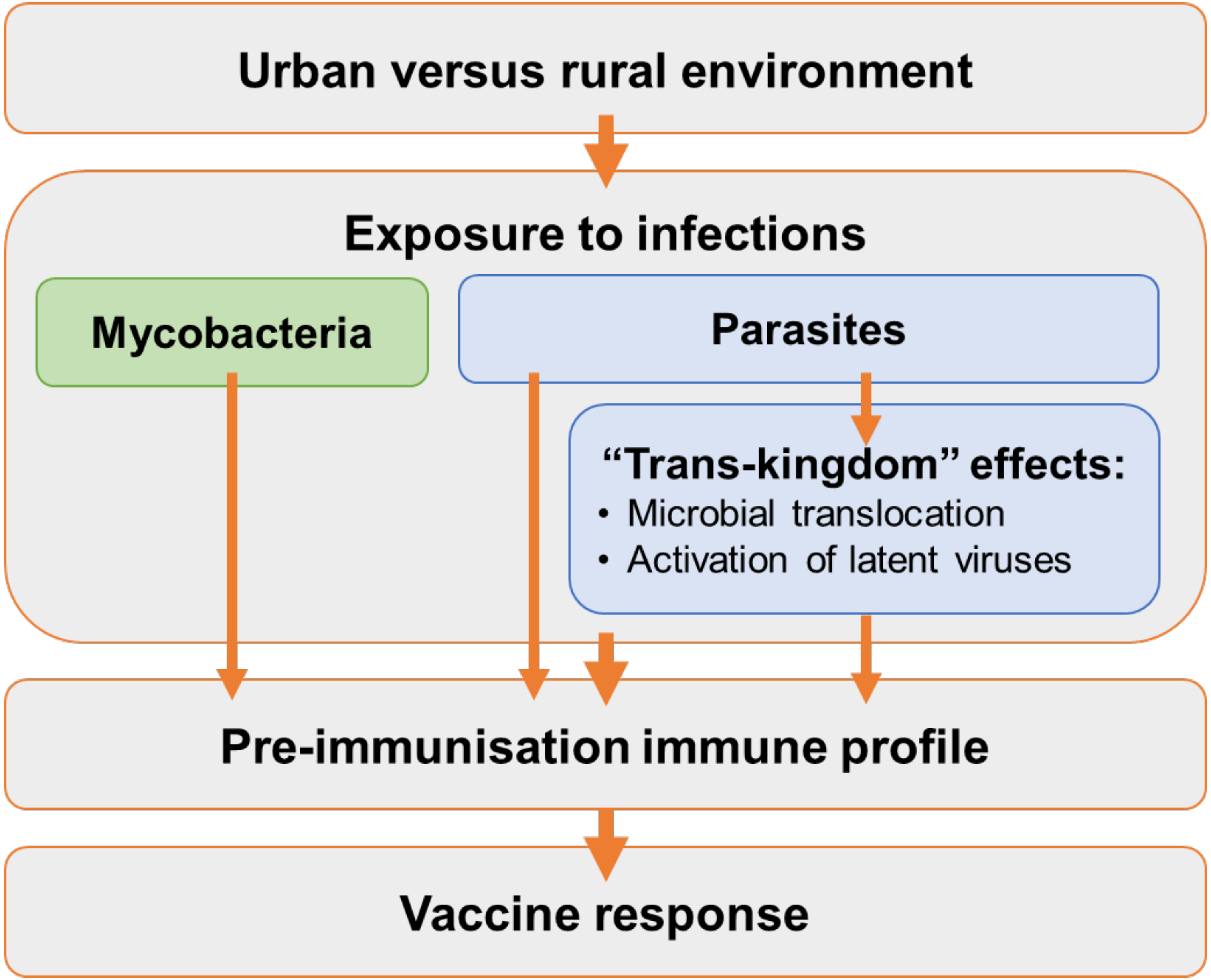
Urban versus rural environment
Exposure to infections
Mycobacteria
Parasites
“Trans-kingdom” effects:
• Microbial translocation
• Activation of latent viruses
Pre-immunisation immune profile
Vaccine response

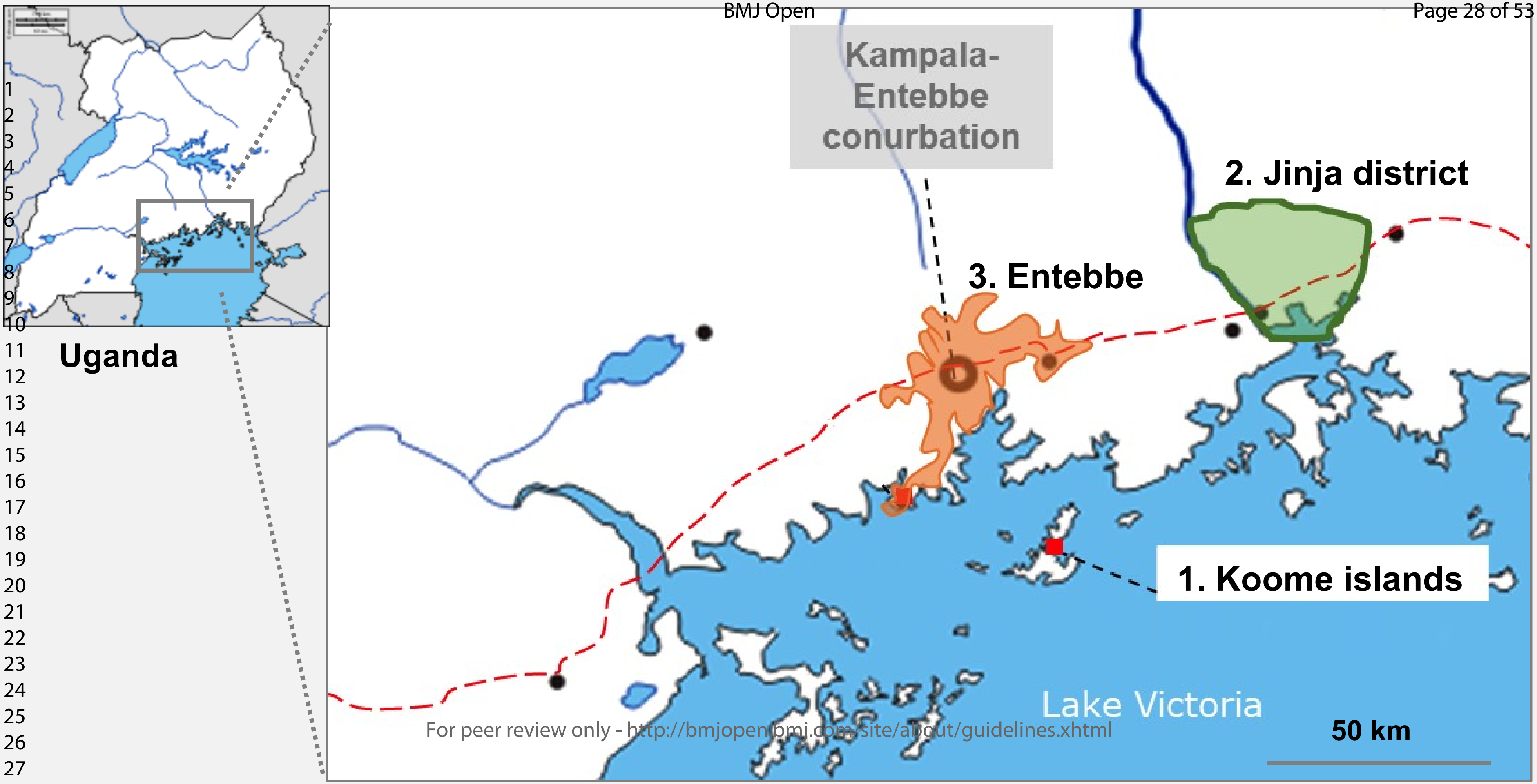
Uganda
Kampala-
Entebbe
conurbation
2. Jinja district
3. Entebbe
1. Koome islands
Lake Victoria
50 km

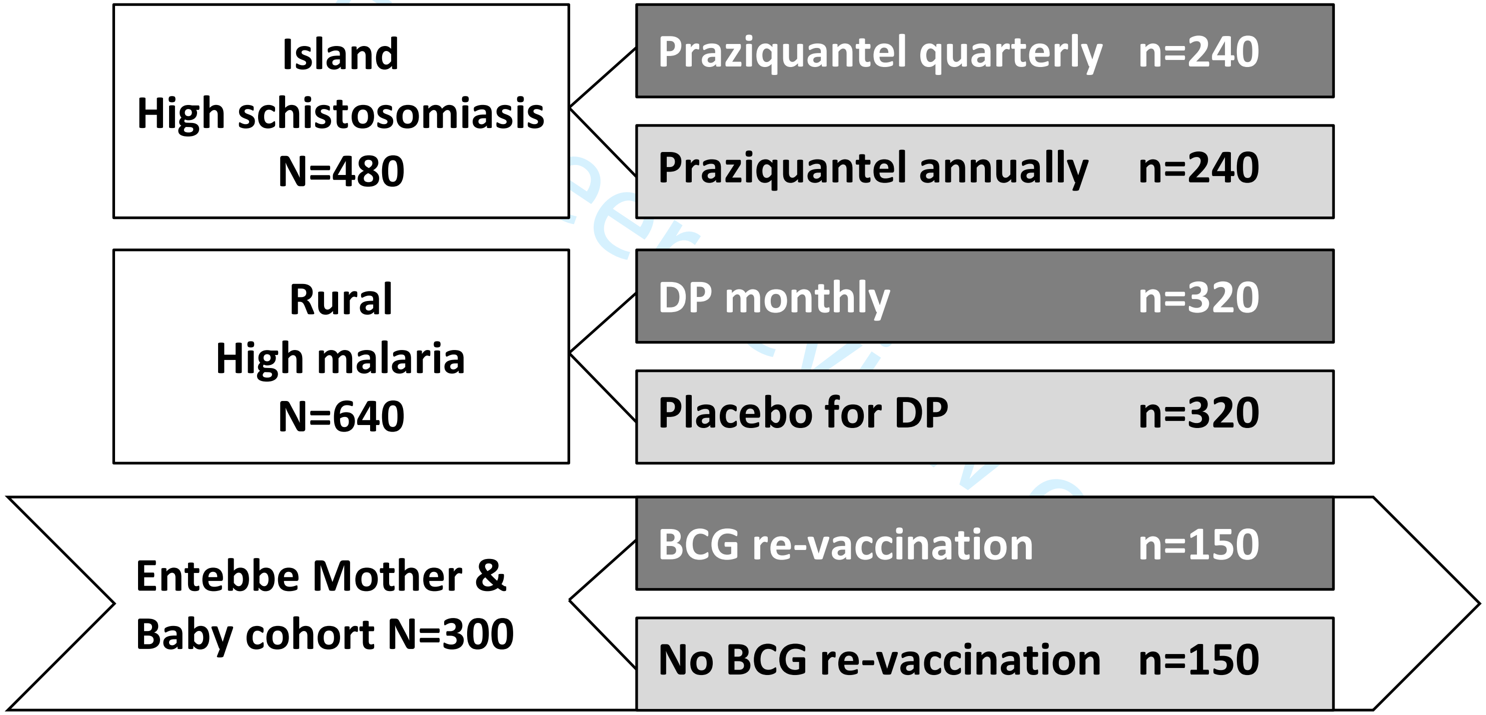
Island
High schistosomiasis
N=480
Praziquantel quarterly n=240
Praziquantel annually n=240
Rural
High malaria
N=640
DP monthly n=320
Placebo for DP n=320
Entebbe Mother & Baby cohort N=300
BCG re-vaccination n=150
No BCG re-vaccination n=150

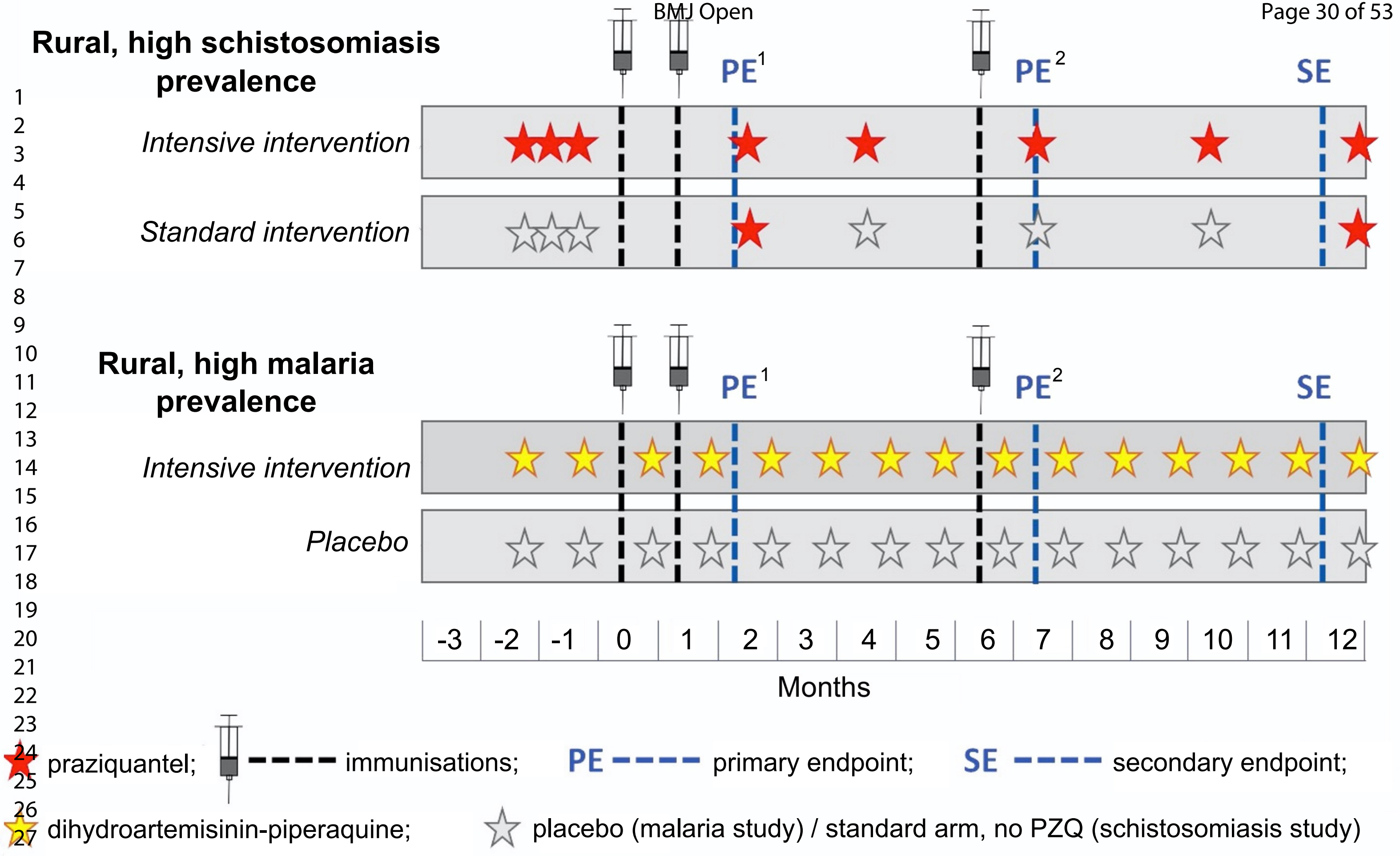
Rural, high schistosomiasis prevalence
PE1
PE2
SE
Intensive intervention
Standard intervention
Rural, high malaria prevalence
PE1
PE2
SE
Intensive intervention
Placebo
-3
-2
-1
0
1
2
3
4
5
6
7
8
9
10
11
12
Months
praziquantel;
immunisations;
PE
primary endpoint;
SE
secondary endpoint;
dihydroartemisinin-piperaquine;
placebo (malaria study) / standard arm, no PZQ (schistosomiasis study)

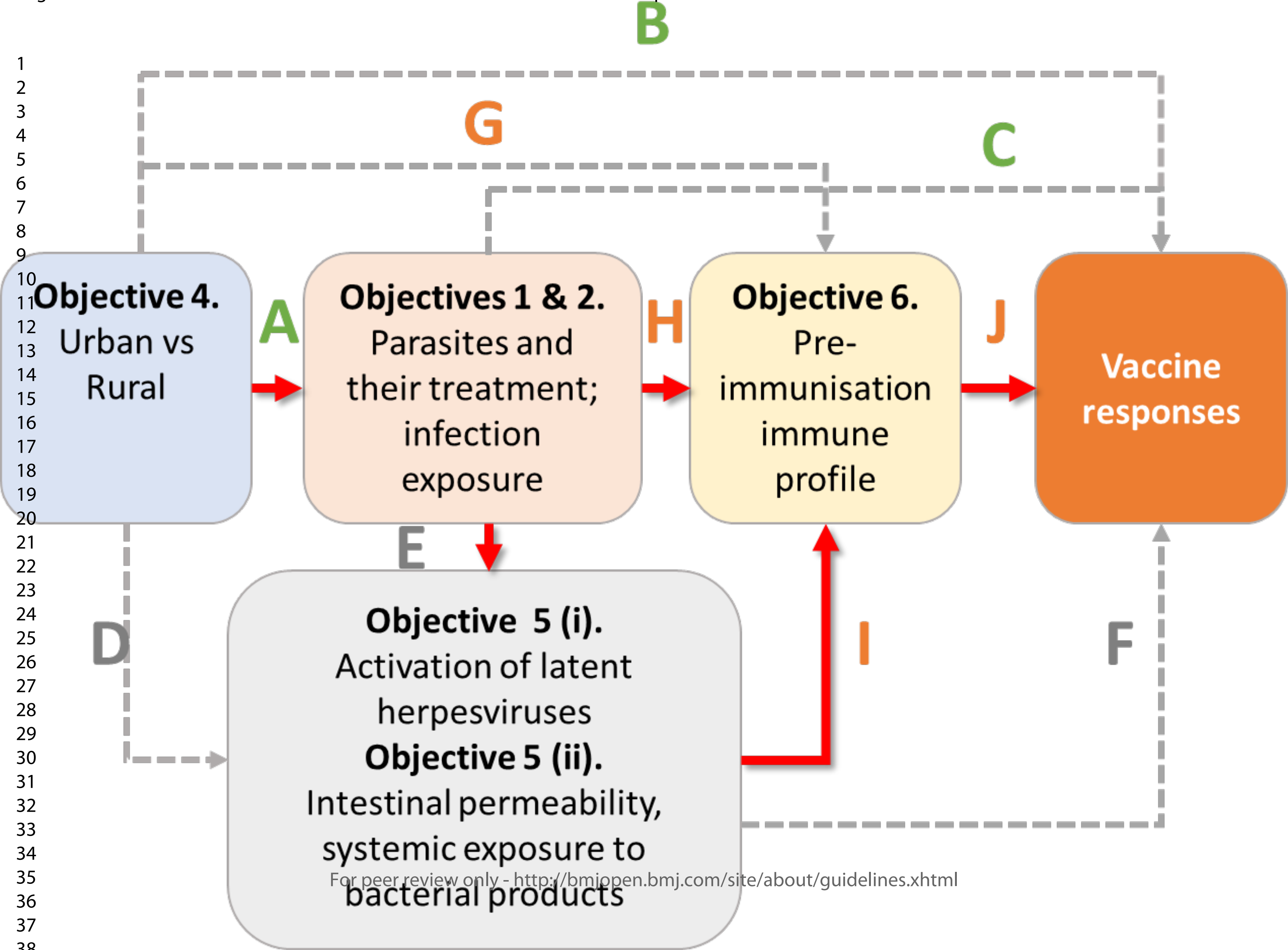
B
G
C
Objective 4.
Urban vs
Rural
A
Objectives 1 & 2.
Parasites and
their treatment;
infection
exposure
H
Objective 6.
Pre-
immunisation
immune
profile
J
Vaccine
responses
E
D
Objective 5 (i).
Activation of latent
herpesviruses
Objective 5 (ii).
Intestinal permeability,
systemic exposure to
bacterial products
I
F

SUPPLEMENTARY INFORMATION

Population differences in vaccine responses (POPVAC): scientific rationale and cross-cutting analyses for three linked, randomised controlled trials assessing the role, reversibility and mediators of immunomodulation by chronic infections in the tropics

Gyaviira Nkurunungi[1,¶,*], Ludoviko Zirimenya[1,¶], Agnes Natukunda[1,¶], Jacent Nassuuna[1], Gloria Oduru[1], Caroline Ninsiima[1], Christopher Zziwa[1], Florence Akello[1], Robert Kizindo[1], Mirriam Akello[1], Pontiano Kaleebu[1], Anne Wajja[1], Henry Luzze[2], Stephen Cose[1,3], Emily L Webb[4], Alison M Elliott[1,5] **for the POPVAC trial team**

[1]Immunomodulation and Vaccines Programme, Medical Research Council/Uganda Virus Research Institute and London School of Hygiene and Tropical Medicine (MRC/UVRI and LSHTM) Uganda Research Unit, Entebbe, Uganda

[2]Uganda National Expanded Program on Immunisation, Ministry of Health, Kampala, Uganda

[3]Department of Clinical Research, London School of Hygiene and Tropical Medicine, London, United Kingdom

[4]MRC Tropical Epidemiology Group, Department of Infectious Disease Epidemiology, London School of Hygiene and Tropical Medicine, London, United Kingdom

[¶]These authors contributed equally

***Correspondence:** Gyaviira Nkurunungi; gyaviira.nkurunungi@lshtm.ac.uk

For peer review only

Table S1. Uganda National Expanded Programme on Immunisation (EPI) schedule

Vaccine/ antigen	Dosage and doses required	Minimum Interval Between Doses	Minimum Age to Start	Mode and site of Administration	Storage Temperatures
**Infant vaccines**					
BCG	Infants (0-11m) 0.05ml. ≥11 months and 0.1ml, 1 dose	Not applicable	At birth (or first contact)	*Intradermal*, right upper arm	+2^{0}C to +8^{0}C
DPT - HepB - Hib	0.5 ml, 3 doses	One month (4 weeks)	At 6 weeks or first contact after this age	*Intramuscular,* outer upper aspect of left thigh	+2^{0}C to +8^{0}C DO NOT FREEZE
PCV	0.5 mls, 3 doses	One month (4 weeks)	At 6 weeks or first contact after this age	*Intramuscular,* outer upper aspect of right thigh	+2^{0}C to +8^{0}C DO NOT FREEZE
Polio	2 drops, 3 doses	One month (4 weeks)	At birth or within the first 2 weeks (Polio 0) and at 6 weeks or first contact after 6 weeks (Polio 1)	*Orally*	+2^{0}C to +8^{0}C
IPV	0.5ml, 1 dose	Nil	At 14 weeks	*Intramuscular,* left upper thigh	+2^{0}C to +8^{0}C DO NOT FREEZE
Rotavirus	drops, 2 doses		6 weeks or 1st contact after this age	*Orally*	
Measles	0.5 ml, 1 doses	Nil	At 9 months (or first contact after that age).	*Subcutaneous,* left upper arm	+2^{0}C to +8^{0}C
Primary school/adolescent/adult vaccines					
Tetanus/Diphtheria	0.5 ml, 5 doses	Td1: First contact with a WCBA<br>Td2: One month after TT1<br>Td3: Six months after TT2<br>Td4: One year after TT3<br>Td5: One year after TT4	At first contact with a pregnant woman or women of childbearing age (15-49 years)	*Intramuscular,* upper arm	+2^{0}C to +8^{0}C, DO NOT FREEZE
HPV	0.5 ml, 2 doses	HPV1: First contact with a girl in primary 4, or aged 10 years and out of school<br>HPV2: Given at 6 months after HPV1[a]	Girls in primary 4 or 10-year-old girls who are out of school	*Intramuscular,* upper arm	+2^{0}C to +8^{0}C, DO NOT FREEZE

BCG: Bacillus Calmette Guerin. DPT: Diphtheria, Pertussis, Tetanus. Hep B: Hepatitis B. Hib: Haemophilus influenzae type B. PCV: pneumococcal conjugate vaccine. IPV: inactivated polio vaccine. HPV: Human Papilloma Virus. WCBA: woman of child-bearing age. [a]An additional dose of HPV, four weeks after the first dose, is recommended for girls aged 14 years or above receiving HPV immunisation for the first time.

For peer review only

Further rationale for the selection of vaccines

Bacillus Calmette–Guérin (BCG)

BCG is a live, replicating parenteral vaccine, the only licensed vaccine against TB. BCG vaccine for these studies will be obtained from the Serum Institute of India either directly, or through a supplier in Uganda. The Serum Institute of India provides much of the BCG vaccine used in Uganda.

Worldwide, TB is among the top 10 causes of death; Uganda has an estimated incidence of 202/100,000 people.[1] Infectious, sputum positive, pulmonary TB classically emerges in adolescence, driving the on-going epidemic.[2] Thus adolescent booster immunisation is a key TB control strategy.[3] However, BCG vaccine response and efficacy are often impaired in tropical and rural settings[4-6] and new TB vaccines are similarly affected.[7] In the past, WHO has been hesitant to recommend BCG re-vaccination. However, in 2017 WHO's Strategic Advisory Group of Experts (SAGE) recommended: "Further research is warranted to explore whether certain sub-groups of age, geographic or *M. tuberculosis* exposure categories would benefit from re-vaccination."[8] Recent results suggest that, despite the variability of BCG efficacy between populations, BCG vaccination in adolescence offers benefit in some tropical settings, especially for individuals who are not yet infected with *Mycobacterium tuberculosis*, and may also be cost-effective.[5 9] Also, BCG vaccine is currently being used among adolescents in South Africa as a comparator in a trial of a novel TB vaccine (trial registration NCT02075203). To our knowledge, BCG efficacy in Ugandan adolescents, and differences in BCG vaccine responses between urban and rural Ugandan populations, have not been tested. Information obtained from this study is expected to further inform the use of BCG in adolescents, and also to inform the development of new vaccines for tuberculosis.

Yellow fever vaccine

Yellow fever vaccine YF-17D is a live replicating parenteral vaccine. The vaccine (Stamaril; Sanofi Pasteur) is available for purchase in Uganda. Yellow Fever (YF) causes outbreaks in Uganda and the wider region[10] and YF-17D is a candidate for Uganda's expanded programme on immunisation (EPI). Lower vaccine replication, lower neutralising antibody induction, and greater waning, are described in Uganda compared to Switzerland.[11] YF-17D is a potential vector for novel vaccine constructs,[12] adding relevance to vaccine development.

Typhoid vaccine Ty21a

Typhoid vaccine Ty21a is a live replicating oral vaccine and also a potential vector for new vaccine constructs.[13] Ty21a vaccine will be purchased from PaxVax, Redwood City, California. Substantial, multi-year typhoid outbreaks occur in Uganda and immunisation campaigns have been advocated as

For peer review only

cost effective.[14] Schistosomiasis has been associated with prolonged *S. typhi* infection[15] and impaired antibody responses to killed typhoid vaccines.[16]

Ty21a was developed in the 1970s. Although not routinely used in Uganda, it has been (and is currently) registered in many countries. It was first registered in the United States and United Kingdom in the 1980s, and is recommended by the WHO for both endemic and epidemic settings.[17] It has comparable efficacy to the parenteral Vi polysaccharide typhoid vaccine, good durability and minimal adverse effects.[17] It is proposed for use in this study to model effects of study exposures and intervention on the response to a live oral vaccine.

The Ty21a vaccine is given as a three-dose regimen on alternate days.

Human Papilloma Virus (HPV) vaccine

Human Papilloma Virus (HPV) vaccine is a protein virus-like particle. The quadrivalent HPV Vaccine Gardasil (Merck) is available for purchase in Uganda and is the vaccine used by the national EPI programme. Studies after three vaccine doses have found somewhat enhanced responses in the presence of malaria, but no effect of helminths.[18] No study has previously investigated parasite effects on the priming response, but recent results for tetanus suggest that priming may be more susceptible than boosting to adverse effects.[19] This will be important if forthcoming trials support single-dose HPV immunisation (NCT02834637). HPV immunisation is being rolled out among girls to prevent cervical neoplasia, the commonest cancer among Ugandan women and we will coordinate provision with the national HPV immunisation programme.[20] HPV immunisation is also beneficial for boys since HPV infection is associated with anogenital warts, anal cancer and oropharyngeal cancers in both males and females, and with penile cancer in men,[21] and we will include boys in these studies.

Tetanus and diphtheria vaccines

Tetanus and diphtheria vaccines comprise inert toxoids (Td). Schistosomiasis is associated with a Th2 biased response to tetanus toxoid[22] and with suppressed antibody responses among those with low pre-immunisation antibody levels.[19] Booster immunisation is recommended for young women to prevent maternal and neonatal tetanus. Recent evidence emphasises the need to protect young men also.[23] Uganda's EPI programme recommends tetanus boosters in adolescence and plans to change from tetanus alone to Td in 2018.

For peer review only

For peer review only - http://bmjopen.bmj.com/site/about/guidelines.xhtml

Additional considerations regarding the vaccine schedule

Live vaccines given in combination may influence the response to each other – a phenomenon described as "interference". Observations in the 1960s suggested that elevated circulating interferon(IFN)-γ after measles immunisation might interfere with the response to Vaccinia[24] and, to avoid such interference between live vaccines, it was recommended that live vaccines be given either together or three to four weeks apart.[25] However, with the introduction of new, live vaccines into use, Public Health England reviewed and revised this recommendation in 2014, limiting it to vaccines for which there was an evidence base (**Table S2**). We have adopted a four-week interval between BCG immunisation and the other proposed live vaccines (YF and Ty21a), which will be given together.[26]

Non-live vaccines can be given at the same time as live vaccines and there are no specific recommendations as to the number of non-live vaccines that can be given together. Two injections can be given into the same muscle although it is suggested that these should be at least 2.5 cm apart in case it is necessary to distinguish local adverse reactions to the two injections.[27] Our schedule avoids giving more than two injections on the same date but at week 4 it may be appropriate to give two into the deltoid muscle of the same arm if a BCG scar is developing on the other arm. Nevertheless, uncertainties remain regarding the effects of vaccines on responses to each other. Generalisations from this programme of work will need to take potential "interference" between vaccines into account.

Table S2: Public Health England recommendations for giving more than one live attenuated vaccine in current use in the UK[26]

Vaccine combinations	Recommendations
Yellow Fever and Measles, Mumps, Rubella (MMR)	A four-week minimum interval period should be observed between the administration of these two vaccines. Yellow Fever and MMR should not be administered on the same day.
Varicella (and zoster) vaccine and MMR	If these vaccines are not administered on the same day, then a four-week minimum interval should be observed between vaccines.
Tuberculin skin testing (Mantoux) and MMR	If a tuberculin skin test has already been initiated, then MMR should be delayed until the skin test has been read unless protection against measles is required urgently. If a child has had a recent MMR, and requires a tuberculin test, then a four-week interval should be observed.
All currently used live vaccines (BCG, rotavirus, live attenuated influenza vaccine (LAIV), oral typhoid vaccine, yellow fever, varicella, zoster and MMR) and tuberculin (Mantoux) skin testing.	Apart from those combinations listed above, these live vaccines can be administered at any time before or after each other. This includes tuberculin (Mantoux) skin testing.

For peer review only - http://bmjopen.bmj.com/site/about/guidelines.xhtml

Vaccine storage and transport

In order to maintain a reliable vaccine cold chain, the vaccines and diluents to be used will be stored and transported within the recommended temperature range of +2°C to +8°C. Care will be taken to ensure that the vaccines are not frozen. BCG, being sensitive to light, will be kept in the dark (normally within its secondary packaging) for as long as possible to protect it during storage and transportation. All vaccines will be kept in appropriate refrigeration equipment with a temperature monitoring device to ensure temperatures remain between +2°C and +8°C. Cold boxes/vaccines carriers with temperature monitors will be used to transport vaccines and the diluents from the MRC/UVRI and LSHTM Uganda Research Unit (Entebbe) to Koome island and while transporting vaccines to immunization sessions. Designated staff will be given responsibility for managing the vaccine cold chain. All cold chain equipment including the temperature monitoring devices used for this project will comply with relevant technical specifications as defined by the EPI standards. Basic routine maintenance will be regularly carried out on all cold chain equipment.

Additional laboratory measurements

Additional assays will comprise HIV serology, pregnancy testing and full blood counts. HIV testing and pregnancy testing will be accompanied by appropriate counselling by trained staff.

- HIV serology will be done on blood samples using rapid tests and according to prevailing national algorithms.[28] This will be done at baseline.
- Pregnancy testing will be done using urine samples and standard operating procedures for assessment of urine β-human chorionic gonadotropin (βhCG). This will be done at baseline and before immunisation on each immunisation day.
- Full blood counts will be conducted using a haematology analyser. Mild, moderate and severe anaemia will be defined according to WHO guidelines, by age.[29] This will be done at baseline to test for anaemia as part of the eligibility assessment, and pre-immunisation as part of the assessment of immunological profile.

Individuals found to be HIV positive or pregnant will be referred to appropriate providers for further care. Individuals with severe anaemia (haemoglobin <82g/L) will be excluded from the randomised intervention (since the intervention might be beneficial in management of anaemia). They will be treated for anaemia and for any underlying cause identified.

For peer review only

Sample handling and archive

Blood and other samples will be processed according to local laboratory standard operating procedures (SOPs). All samples will reach the laboratory in anonymised form.

A sample archive will be developed. Although our current programme of work plans to address specific hypotheses regarding pathways of effects of parasites and interventions, the sample archive will provide a major asset for exploration of new leads arising from this work, or for an alternative, "systems biology" approach employing (for example) proteomic, genomic, epigenetic and transcriptomic analyses, and investigating the microbiome and virome. Information provided to participants, and consent forms, will include considerations of sample storage, and the possibility of sample analysis in laboratories within and outside Uganda. Participants will be able to decide if they will permit such future use of any leftover samples. We plan to store the samples for up to 20 years. If further storage is needed after that time, permission will be requested from the Uganda Virus Research Institute and London School of Hygiene and Tropical Medicine review committees. If they elect not to permit this, all of those leftover samples will be discarded after the completion of the work included in the current protocol.

For peer review only

Operational considerations

Programme governance

A Programme Steering Committee has been set up to guide progress across all projects. This comprises the following:

- An independent chair
- Representatives from the Ministry of Health programmes for immunisation and for vector borne disease control
- Representatives of district authorities (Mukono and Jinja districts)
- Community representatives
- Principal investigator and co-investigators
- Project leader and post-doctoral immunologist
- Trial statistician
- Laboratory manager
- Medical Research Council observer

Informed consent

Both written informed assent from the participants and written informed consent from a parent or guardian will be required for participation, although these may not necessarily be obtained at the

For peer review only - http://bmjopen.bmj.com/site/about/guidelines.xhtml

same time. Information will be provided in both English and the appropriate local language. For individuals who cannot speak the languages used, or who cannot read or write, a witness who can read the information sheet and translate the information to the participant or parent/guardian will be used. For trials A and B, two different types of age specific assent forms will be used for the group of participants aged 9 – 12 years and for the group aged 13 – 17 years. Informed consent by emancipated or mature minors will be obtained using a designated consent form for these categories of participants

The aims of the study, all tests, treatments and immunisations to be carried out and potential risks will be explained. The participant will be given the opportunity to ask about details of the trial, and will then have time to consider whether or not to participate. If they do decide to participate, they and their parent/guardian will sign and date two copies of the assent and consent forms, one for them to take away and keep, and one to be stored securely by the research team. Separate information and consent forms will be provided (i) for consent for storage of samples for future studies and for anonymous sharing of data from this study and (ii) for possible genetic studies (for the urban cohort genetic data are already available based on previous approval); the information sheet will explain that these data may be used in analyses related to this protocol.

Screening and Eligibility Assessment

Once the informed consent process has been completed, and consent (and assent) given, a baseline medical history (including concomitant medication) will be collected. Vital signs will be checked and a physical examination will be performed. Inclusion and exclusion criteria will be checked.

Participants will undergo pre- and post-test counselling for HIV and (for girls) pregnancy testing by a trained and experienced nurse- or clinician-counsellor. Blood, urine and stool samples will be obtained, for tests as specified in the schedule of procedures. These tests are to exclude the major, immunomodulating co-infection, HIV, and conditions that might impact safety (anaemia, pregnancy).

Enrolment

Participants who consent/assent, complete the screening processes, satisfy all the inclusion criteria and meet none of the exclusion criteria will be enrolled.

Discontinuation / withdrawal criteria

In accordance with the principles of the current revision of the Declaration of Helsinki and any other applicable regulations, a participant has the right to withdraw from the study at any time and for any reason, and is not obliged to give his or her reasons for doing so. The Investigator may withdraw the

For peer review only

participant at any time in the interests of the participant's health and well-being. In addition, the participant may withdraw/be withdrawn for any of the following reasons:

- Ineligibility (either arising during the study or retrospectively, having been overlooked at screening)
- Administrative decision by the Investigator
- Significant protocol deviation
- Participant non-compliance with study requirements
- An adverse event which requires discontinuation of the study involvement or results in inability to continue to comply with study procedures.

Any participant who becomes pregnant during the trial will be followed up until the end of the pregnancy but no further immunisations will be given unless indicated during pregnancy (as is the case for tetanus toxoid). The trial allocation for this participant will be unblinded and the participant will only be given further treatment if clinically indicated. The babies will also be followed up and examined for any adverse effects. We will not routinely perform venipuncture in a pregnant participant.

The reason for withdrawal will be recorded in the case report form (CRF). If withdrawal is due to an AE, appropriate follow-up visits or medical care will be arranged, with the agreement of the participant, until the AE has resolved, stabilised or a non-trial related causality has been assigned.

If a participant withdraws from the study samples collected before their withdrawal from the trial will be used/ stored unless the participant specifically requests otherwise.

Trial discontinuation

The trial will be discontinued in the event of new scientific information that renders continuation futile or unethical, or for any other reason, at the discretion of the Programme Steering Committee.

End of study definition

The trial will be completed when the last participant enrolled into the trial has completed their final follow up visit.

Safety assessments and oversight

No new investigational drug or product will be used in the proposed trial. However, standard approaches for monitoring safety and reporting of serious adverse events will be followed.

For peer review only

Monitoring

The trial will be monitored by both internal and external monitors according to a pre-defined monitoring plan which will include a site initiation visit, monitoring visits at least annually, and a close-out visit. The monitors will assess patient safety, data integrity, and adherence to the protocol and to Good Clinical Research Practice procedures.

Considerations regarding standard of care for parasitic infections

Malaria and *S. mansoni* infection status will be determined retrospectively through assays conducted in bulk on stored samples. These results will not, therefore, be useful to determine management of individual participants.

Participants in the standard anthelminthic treatment (trial A) and the malaria placebo (trial B) arms will receive lower levels of treatment. However, all trial arms will receive a minimum of well-implemented national standard of care.

In trial A, standard of care will comprise annual praziquantel treatment. Our own results from the Lake Victoria Island Intervention Study on Worms and Allergy-related diseases (LaVIISWA),[30] which compared annual versus quarterly intervention for schistosomiasis at community level over three years, showed no advantage of quarterly treatment for morbidity outcomes attributed to schistosomiasis. Schistosomiasis can cause anaemia. To manage the expected differential benefits of the interventions for anaemia, a full blood count will be performed at baseline, as discussed above; anaemic children will be managed appropriately and severely anaemic children excluded. Albendazole will be provided twice a year to manage nematode infections (after collection of primary and secondary endpoint samples).

Dihydroartemisinin/piperaquine is considered an attractive option for preventive treatment and preventive chemotherapy for malaria because of the long half-life of piperaquine (approximately 23 days).[31] Monthly treatment with DP has been shown to reduce the prevalence of anaemia and reduce episodes of clinical malaria in Ugandan schools[32] but has not been adopted as standard of care. Trial B is expected to add further evidence regarding the potential benefits of monthly DP for school children by determining the effect on vaccine responses, thereby further contributing to policy debate in this field. To manage the expected differential benefits of the interventions for anaemia, a full blood count will be performed at baseline, as discussed above; anaemic children will be managed appropriately and severely anaemic children excluded. Malaria standard of care will comprise provision of bed nets to minimise malaria exposure for all participants. Rapid diagnostic tests and treatment will be made readily available for participants who develop symptomatic

For peer review only

For peer review only - http://bmjopen.bmj.com/site/about/guidelines.xhtml

malaria. Albendazole will be provided twice a year to manage nematode infections (after collection of primary and secondary endpoint samples).

Procedures to be followed in the event of abnormal findings

Abnormal clinical findings from medical history, examination or blood tests will be assessed as to their clinical significance throughout the trials. If an abnormal test result is deemed clinically significant, it may be repeated. If a test remains clinically significant, the participant will be informed and appropriate medical care arranged as appropriate and with the permission of the participant. Specific details regarding findings, discussion with participants and resulting actions will be recorded in the clinical records. Decisions to exclude the participant from enrolling in the trial or to withdraw a participant from the trial will be at the discretion of the Investigator.

Data and Safety Monitoring Board (DSMB)

The DSMB will be notified within 7 days of the Investigators' being aware of the occurrence of SAEs. The DSMB may recommend the Investigators to place the trial on hold if deemed necessary following an intervention-related SAE. The DSMB will be chaired by a clinician experienced in clinical trials. There will be a minimum of two other appropriately qualified committee members. In the case of events related to a blinded intervention, the DSMB can request unblinding. Membership will include a statistician, and at least one Ugandan member. All correspondence between Investigators and the DSMB will be conveyed by the Principal Investigator to the trial Sponsor. The Chair of the DSMB will be contacted for advice and independent review by the Investigator or trial Sponsor in the following situations:

- The occurrence of any SAE
- Any other situation where the Investigator or trial Sponsor feels independent advice or review is important

Ethical and regulatory considerations

Further information regarding risks

The immunisations to be given have recognised side effects which are usually mild and resolve spontaneously in a few days to one week. Parenteral vaccines are likely to result in pain and swelling at the site of injection and mild fever; very occasionally pain and swelling can be severe and associated with difficulty in moving the shoulder. Sometimes headache and tiredness occur. Rarely a vaccine may cause a severe allergic reaction. For most vaccines this is estimated at less than one in a million doses (but 1 in 55,000 for Yellow Fever vaccine).[33] Individuals with a history of a possible allergic reaction to drugs or vaccines, or to vaccine components including eggs or chicken

For peer review only - http://bmjopen.bmj.com/site/about/guidelines.xhtml

proteins, will be excluded from the studies. The research team will be trained and prepared to manage severe allergic reactions.

Adverse reactions to Yellow Fever vaccine include severe nervous system reaction (about 1 person in 125,000) and severe, life-threatening illness with organ failure (about 1 person in 250,000). The mortality for this severe, life-threatening adverse effect is reported as about 50%.[33]

BCG immunisation is likely to induce a scar in many cases. This may develop over several weeks, starting as a small papule at the injection site which may become ulcerated and then heal over a period of 2 to 5 months; and lymphadenopathy may develop. Occasionally a more severe local reaction occurs (estimated at 1 per 1,000-10,000 doses): for example, an abscess develops and scars may develop into keloids. Rarely BCG can cause disseminated disease (1 per 230,000 to 640,000 doses), or disease in sites remote from the immunisation site. Disseminated BCG disease usually occurs in immunocompromised people: HIV positive people will be excluded from these studies.[34] BCG "pre-immunisation" may interfere with the response to the subsequent live vaccines; indeed our hypothesis, and published results, suggest that it may suppress replication of YF 17D vaccine.[35] However, this reduced replication has not been shown to correlate with, or result in, reduced levels of neutralising antibody titres (which are the desired protective outcome).[11 35]

Oral typhoid vaccine (Ty21a) may occasionally be associated with stomach pain, nausea, vomiting and (rarely) rash.[33]

Praziquantel has been in use for about 30 years. It has a well-recognised profile of side effects including dizziness, nausea, vomiting, abdominal pain, diarrhoea (sometimes with blood) and urticarial rash. The symptoms are considered to arise largely from the effects of killing worms and to be more severe in people with heavy infections. Symptoms are better tolerated when the drugs are given after food and we will provide treatment after a meal or snack. Simple medications, such as paracetamol and cetirizine, can alleviate symptoms and these will be available on treatment days.

More information on causal mediation analysis

Causal mediation analysis is a statistical approach that aims to assess the relative importance of intermediate variables (mediators) through which an exposure may affect an outcome. It uses a counterfactual framework. In essence, this investigates how, for each individual, values of the mediator and outcome might change if their main exposure status changed. For example, in objective iv, it will be used in exploratory analysis to assess whether, and to what degree, any differences in vaccine response (the outcome) by setting (the exposure) may be explained by differences in current or previous *S. mansoni* and *P. falciparum* infection experience (the potential

For peer review only

For peer review only - http://bmjopen.bmj.com/site/about/guidelines.xhtml

mediators). An alternative approach would be to use a path analytic approach such as structural equation modelling, but estimation of pathway-specific effects using this technique requires strong assumptions regarding linearity and normality and lack of pairwise confounding for all variables in the model. Causal mediation analysis is itself subject to key assumptions regarding confounding between exposure, mediator and outcomes, and we will assess the potential impact of violations in these assumptions using sensitivity analysis. Specifically, we will assess the degree of uncontrolled confounding that would substantially change conclusions regarding pathway-specific effects using techniques such as those described by Hafemen (2011).[36] Regarding the mediators for objective 1, we will consider *S. mansoni* and *P. falciparum* separately, under the assumption that there is no direct causal link between these infections. For each infection, we will include both prior exposure and current infection as mediators in the same set of models (**Figure S1**), allowing the possibility that current infection depends on prior exposure, using the approach outlined in Steen *et al*.[37]

Figure S1. Causal diagram for assessment of whether exposure to parasites mediates differences in vaccine response between urban and rural settings

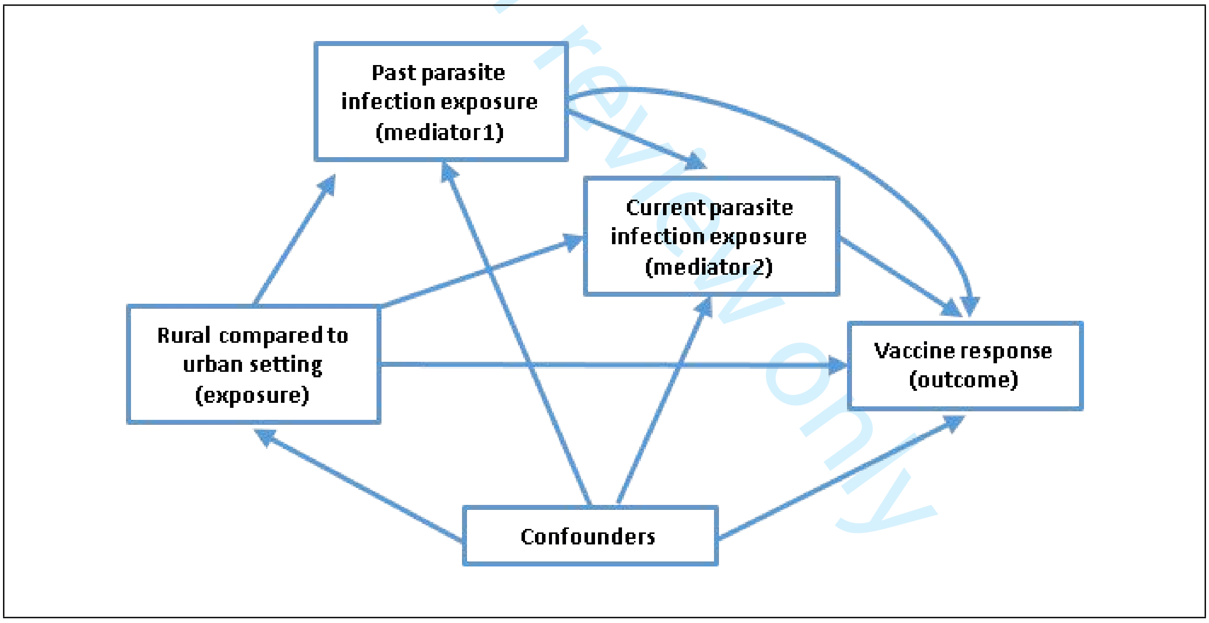

For peer review only - http://bmjopen.bmj.com/site/about/guidelines.xhtml

References

1. WHO. Global tuberculosis report 2016 http://www.who.int/tb/publications/global_report/en/ (accessed 17 June 2017), 2016.

2. Alcais A, Fieschi C, Abel L, et al. Tuberculosis in children and adults: two distinct genetic diseases. *J Exp Med* 2005;202(12):1617-21. doi: 10.1084/jem.20052302

3. Weiner J, 3rd, Kaufmann SH. Recent advances towards tuberculosis control: vaccines and biomarkers. *J Intern Med* 2014;275(5):467-80. doi: 10.1111/joim.12212

4. Fine PE. Variation in protection by BCG: implications of and for heterologous immunity. *Lancet* 1995;346(8986):1339-45.

5. Barreto ML, Pilger D, Pereira SM, et al. Causes of variation in BCG vaccine efficacy: examining evidence from the BCG REVAC cluster randomized trial to explore the masking and the blocking hypotheses. *Vaccine* 2014;32(30):3759-64. doi: 10.1016/j.vaccine.2014.05.042

6. Black GF, Weir RE, Floyd S, et al. BCG-induced increase in interferon-gamma response to mycobacterial antigens and efficacy of BCG vaccination in Malawi and the UK: two randomised controlled studies. *Lancet* 2002;359(9315):1393-401. doi: 10.1016/S0140-6736(02)08353-8

7. Tanner R, Kakalacheva K, Miller E, et al. Serum indoleamine 2,3-dioxygenase activity is associated with reduced immunogenicity following vaccination with MVA85A. *BMC infectious diseases* 2014;14:660. doi: 10.1186/s12879-014-0660-7 [published Online First: 2014/12/04]

8. WHO. SAGE Evidence to recommendations framework. 2017. http://www.who.int/immunization/sage/meetings/2017/october/2_EvidencetoRecommendationFramework_BCG.pdf (accessed 16th March 2018).

9. Dye C. Making wider use of the world's most widely used vaccine: Bacille Calmette-Guerin revaccination reconsidered. *Journal of the Royal Society, Interface* 2013;10(87):20130365. doi: 10.1098/rsif.2013.0365 [published Online First: 2013/08/02]

10. Kraemer MU, Faria NR, Reiner RC, Jr., et al. Spread of yellow fever virus outbreak in Angola and the Democratic Republic of the Congo 2015-16: a modelling study. *The Lancet Infectious diseases* 2016 doi: 10.1016/s1473-3099(16)30513-8 [published Online First: 2016/12/27]

11. Muyanja E, Ssemaganda A, Ngauv P, et al. Immune activation alters cellular and humoral responses to yellow fever 17D vaccine. *The Journal of clinical investigation* 2014;124(7):3147-58. doi: 10.1172/jci75429 [published Online First: 2014/06/10]

12. Aguiar M, Stollenwerk N, Halstead SB. The Impact of the Newly Licensed Dengue Vaccine in Endemic Countries. *PLoS neglected tropical diseases* 2016;10(12):e0005179. doi: 10.1371/journal.pntd.0005179 [published Online First: 2016/12/22]

13. Dharmasena MN, Osorio M, Filipova S, et al. Stable expression of Shigella dysenteriae serotype 1 O-antigen genes integrated into the chromosome of live Salmonella oral vaccine vector Ty21a. *Pathogens and disease* 2016 doi: 10.1093/femspd/ftw098 [published Online First: 2016/09/23]

14. Carias C, Walters MS, Wefula E, et al. Economic evaluation of typhoid vaccination in a prolonged typhoid outbreak setting: the case of Kasese district in Uganda. *Vaccine* 2015;33(17):2079-85. doi: 10.1016/j.vaccine.2015.02.027 [published Online First: 2015/02/26]

15. Melhem RF, LoVerde PT. Mechanism of interaction of Salmonella and Schistosoma species. *Infection and immunity* 1984;44(2):274-81. [published Online First: 1984/05/01]

For peer review only

For peer review only - http://bmjopen.bmj.com/site/about/guidelines.xhtml

16. Muniz-Junqueira MI, Tavares-Neto J, Prata A, et al. Antibody response to Salmonella typhi in human schistosomiasis mansoni. *Revista da Sociedade Brasileira de Medicina Tropical* 1996;29(5):441-5. [published Online First: 1996/09/01]

17. WHO. Position Paper on Typhoid vaccines: WHO position paper – March 2018 2018

18. Brown J, Baisley K, Kavishe B, et al. Impact of malaria and helminth infections on immunogenicity of the human papillomavirus-16/18 AS04-adjuvanted vaccine in Tanzania. *Vaccine* 2014;32(5):611-7. doi: 10.1016/j.vaccine.2013.11.061

19. Riner DK, Ndombi EM, Carter JM, et al. Schistosoma mansoni Infection Can Jeopardize the Duration of Protective Levels of Antibody Responses to Immunizations against Hepatitis B and Tetanus Toxoid. *PLoS neglected tropical diseases* 2016;10(12):e0005180. doi: 10.1371/journal.pntd.0005180

20. Centre HI. HPV and related diseases report: Uganda. 2016. http://www.hpvcentre.net/statistics/reports/UGA.pdf (accessed 20.01.2017).

21. WHO. Human papillomavirus vaccines: WHO position paper, May 2017. *Releve epidemiologique hebdomadaire* 2017;92(19):241-68. [published Online First: 2017/05/23]

22. Sabin EA, Araujo MI, Carvalho EM, et al. Impairment of tetanus toxoid-specific Th1-like immune responses in humans infected with Schistosoma mansoni. *The Journal of infectious diseases* 1996;173(1):269-72. [published Online First: 1996/01/01]

23. Nanteza B, Galukande M, Aceng J, et al. The burden of tetanus in Uganda. *SpringerPlus* 2016;5(1):705. doi: 10.1186/s40064-016-2309-z [published Online First: 2016/06/29]

24. Petralli JK, Merigan TC, Wilbur JR. ACTION OF ENDOGENOUS INTERFERON AGAINST VACCINIA INFECTION IN CHILDREN. *Lancet* 1965;2(7409):401-5. [published Online First: 1965/08/28]

25. C-A S. Vaccine Immunology. In: Plotkin SA, Orenstein W, Offit P, et al., eds. Vaccines: Elsevier 2017.

26. PHE. Public Health England: revised recommendations for the administration of more than one live vaccine 2015

27. PHE. Immunization procedures. The Green Book. Public Health England. 2012

28. Ministry of Health–Uganda. Uganda Clinical Guidelines 2016. http://apps.who.int/medicinedocs/documents/s23532en/s23532en.pdf

29. WHO. Haemoglobin concentrations for the diagnosis of anaemia and assessment of severity. Vitamin and Mineral Nutrition Information System. Geneva, World Health Organization, 2011 (WHO/NMH/NHD/MNM/11.1).

30. Sanya RE, Nkurunungi G, Hoek Spaans R, et al. The Impact of Intensive Versus Standard Anthelminthic Treatment on Allergy-related Outcomes, Helminth Infection Intensity, and Helminth-related Morbidity in Lake Victoria Fishing Communities, Uganda: Results From the LaVIISWA Cluster-randomized Trial. *Clinical Infectious Diseases* 2018:ciy761-ciy61. doi: 10.1093/cid/ciy761

31. Gutman J, Kovacs S, Dorsey G, et al. Safety, tolerability, and efficacy of repeated doses of dihydroartemisinin-piperaquine for prevention and treatment of malaria: a systematic review and meta-analysis. *The Lancet Infectious diseases* 2017;17(2):184-93. doi: 10.1016/s1473-3099(16)30378-4 [published Online First: 2016/11/21]

32. Nankabirwa JI, Wandera B, Amuge P, et al. Impact of intermittent preventive treatment with dihydroartemisinin-piperaquine on malaria in Ugandan schoolchildren: a randomized, placebo-controlled

For peer review only

trial. *Clinical infectious diseases : an official publication of the Infectious Diseases Society of America* 2014;58(10):1404-12. doi: 10.1093/cid/ciu150 [published Online First: 2014/03/14]

33. CDC. Centers for Disease Control and Prevention, vaccines and immunizations.

34. WHO. Information sheet observed rate of vaccine reactions Bacille Calmette-Guérin (BCG) vaccine. 2012

35. Arts RJW, Moorlag S, Novakovic B, et al. BCG Vaccination Protects against Experimental Viral Infection in Humans through the Induction of Cytokines Associated with Trained Immunity. *Cell host & microbe* 2018;23(1):89-100.e5. doi: 10.1016/j.chom.2017.12.010 [published Online First: 2018/01/13]

36. Hafeman DM. Confounding of indirect effects: a sensitivity analysis exploring the range of bias due to a cause common to both the mediator and the outcome. *American journal of epidemiology* 2011;174(6):710-7. doi: 10.1093/aje/kwr173 [published Online First: 2011/06/10]

37. Steen J, Loeys T, Moerkerke B, et al. Flexible Mediation Analysis With Multiple Mediators. *American journal of epidemiology* 2017;186(2):184-93. doi: 10.1093/aje/kwx051 [published Online First: 2017/05/05]

For peer review only

Reporting checklist for protocol of a clinical trial.

Based on the SPIRIT guidelines.

Instructions to authors

Complete this checklist by entering the page numbers from your manuscript where readers will find each of the items listed below.

Your article may not currently address all the items on the checklist. Please modify your text to include the missing information. If you are certain that an item does not apply, please write "n/a" and provide a short explanation.

Upload your completed checklist as an extra file when you submit to a journal.

In your methods section, say that you used the SPIRITreporting guidelines, and cite them as:

Chan A-W, Tetzlaff JM, Altman DG, Laupacis A, Gøtzsche PC, Krleža-Jerić K, Hróbjartsson A, Mann H, Dickersin K, Berlin J, Doré C, Parulekar W, Summerskill W, Groves T, Schulz K, Sox H, Rockhold FW, Rennie D, Moher D. SPIRIT 2013 Statement: Defining standard protocol items for clinical trials. Ann Intern Med. 2013;158(3):200-207

		Reporting Item	Page Number
**Administrative information**			
Title	#1	Descriptive title identifying the study design, population, interventions, and, if applicable, trial acronym	1
Trial registration	#2a	Trial identifier and registry name. If not yet registered, name of intended registry	2
Trial registration: data set	#2b	All items from the World Health Organization Trial Registration Data Set	n/a
Protocol version	#3	Date and version identifier	Information available at ISRCTN60517191, ISRCTN62041885, ISRCTN10482904.
Funding	#4	Sources and types of financial, material, and other support	16

For peer review only

Roles and responsibilities: contributorship	#5a	Names, affiliations, and roles of protocol contributors	16
Roles and responsibilities: sponsor contact information	#5b	Name and contact information for the trial sponsor	Information available at ISRCTN60517191, ISRCTN62041885, ISRCTN10482904.
Roles and responsibilities: sponsor and funder	#5c	Role of study sponsor and funders, if any, in study design; collection, management, analysis, and interpretation of data; writing of the report; and the decision to submit the report for publication, including whether they will have ultimate authority over any of these activities	16
Roles and responsibilities: committees	#5d	Composition, roles, and responsibilities of the coordinating centre, steering committee, endpoint adjudication committee, data management team, and other individuals or groups overseeing the trial, if applicable (see Item 21a for data monitoring committee)	Details in supplementary information
**Introduction**			
Background and rationale	#6a	Description of research question and justification for undertaking the trial, including summary of relevant studies (published and unpublished) examining benefits and harms for each intervention	4
Background and rationale: choice of comparators	#6b	Explanation for choice of comparators	7
Objectives	#7	Specific objectives or hypotheses	6
Trial design	#8	Description of trial design including type of trial (eg, parallel group, crossover, factorial, single group), allocation ratio, and framework (eg, superiority, equivalence, non-inferiority, exploratory)	6,7

For peer review only

**Methods: Participants, interventions, and outcomes**			
Study setting	#9	Description of study settings (eg, community clinic, academic hospital) and list of countries where data will be collected. Reference to where list of study sites can be obtained	6,7
Eligibility criteria	#10	Inclusion and exclusion criteria for participants. If applicable, eligibility criteria for study centres and individuals who will perform the interventions (eg, surgeons, psychotherapists)	7
Interventions: description	#11a	Interventions for each group with sufficient detail to allow replication, including how and when they will be administered	7
Interventions: modifications	#11b	Criteria for discontinuing or modifying allocated interventions for a given trial participant (eg, drug dose change in response to harms, participant request, or improving / worsening disease)	Detailed in supplementary information files
Interventions: adherance	#11c	Strategies to improve adherence to intervention protocols, and any procedures for monitoring adherence (eg, drug tablet return; laboratory tests)	Detailed in supplementary information files
Interventions: concomitant care	#11d	Relevant concomitant care and interventions that are permitted or prohibited during the trial	Detailed in supplementary information files
Outcomes	#12	Primary, secondary, and other outcomes, including the specific measurement variable (eg, systolic blood pressure), analysis metric (eg, change from baseline, final value, time to event), method of aggregation (eg, median, proportion), and time point for each outcome. Explanation of the clinical relevance of chosen efficacy and harm outcomes is strongly recommended	9

For peer review only

Participant timeline	#13	Time schedule of enrolment, interventions (including any run-ins and washouts), assessments, and visits for participants. A schematic diagram is highly recommended (see Figure)	Detailed in supplementary information files for protocols A, B and C
Sample size	#14	Estimated number of participants needed to achieve study objectives and how it was determined, including clinical and statistical assumptions supporting any sample size calculations	9
Recruitment	#15	Strategies for achieving adequate participant enrolment to reach target sample size	Detailed in supplementary information files for protocols A, B and C
**Methods: Assignment of interventions (for controlled trials)**			
Allocation: sequence generation	#16a	Method of generating the allocation sequence (eg, computer-generated random numbers), and list of any factors for stratification. To reduce predictability of a random sequence, details of any planned restriction (eg, blocking) should be provided in a separate document that is unavailable to those who enrol participants or assign interventions	Detailed in protocols A, B and C
Allocation concealment mechanism	#16b	Mechanism of implementing the allocation sequence (eg, central telephone; sequentially numbered, opaque, sealed envelopes), describing any steps to conceal the sequence until interventions are assigned	Detailed in protocols A, B and C
Allocation: implementation	#16c	Who will generate the allocation sequence, who will enrol participants, and who will assign participants to interventions	Detailed in protocols A, B and C
Blinding (masking)	#17a	Who will be blinded after assignment to interventions (eg, trial participants, care	Detailed in protocols A, B and C

For peer review only

		providers, outcome assessors, data analysts), and how	
Blinding (masking): emergency unblinding	#17b	If blinded, circumstances under which unblinding is permissible, and procedure for revealing a participant's allocated intervention during the trial	Detailed in protocols A, B and C
**Methods: Data collection, management, and analysis**			
Data collection plan	#18a	Plans for assessment and collection of outcome, baseline, and other trial data, including any related processes to promote data quality (eg, duplicate measurements, training of assessors) and a description of study instruments (eg, questionnaires, laboratory tests) along with their reliability and validity, if known. Reference to where data collection forms can be found, if not in the protocol	Detailed in protocols A, B and C
Data collection plan: retention	#18b	Plans to promote participant retention and complete follow-up, including list of any outcome data to be collected for participants who discontinue or deviate from intervention protocols	Detailed in protocols A, B and C
Data management	#19	Plans for data entry, coding, security, and storage, including any related processes to promote data quality (eg, double data entry; range checks for data values). Reference to where details of data management procedures can be found, if not in the protocol	Detailed in protocols A, B and C
Statistics: outcomes	#20a	Statistical methods for analysing primary and secondary outcomes. Reference to where other details of the statistical analysis plan can be found, if not in the protocol	11, 12, and in protocols A, B and C. These will also be detailed in a statistical analysis plans that will be uploaded to the online trial registrations.

For peer review only

Statistics: additional analyses	#20b	Methods for any additional analyses (eg, subgroup and adjusted analyses)	11, 12, and in protocols A, B and C. These will also be detailed in a statistical analysis plans that will be uploaded to the online trial registrations.
Statistics: analysis population and missing data	#20c	Definition of analysis population relating to protocol non-adherence (eg, as randomised analysis), and any statistical methods to handle missing data (eg, multiple imputation)	11, 12, and in protocols A, B and C. These will also be detailed in a statistical analysis plans that will be uploaded to the online trial registrations.
**Methods: Monitoring**			
Data monitoring: formal committee	#21a	Composition of data monitoring committee (DMC); summary of its role and reporting structure; statement of whether it is independent from the sponsor and competing interests; and reference to where further details about its charter can be found, if not in the protocol. Alternatively, an explanation of why a DMC is not needed	Detailed in supplementary information files
Data monitoring: interim analysis	#21b	Description of any interim analyses and stopping guidelines, including who will have access to these interim results and make the final decision to terminate the trial	Detailed in supplementary information files
Harms	#22	Plans for collecting, assessing, reporting, and managing solicited and spontaneously reported adverse events and other unintended effects of trial interventions or trial conduct	Detailed in supplementary information files
Auditing	#23	Frequency and procedures for auditing trial conduct, if any, and whether the process will be independent from investigators and the sponsor	Detailed in supplementary information files
**Ethics and dissemination**			

For peer review only

Research ethics approval	#24	Plans for seeking research ethics committee / institutional review board (REC / IRB) approval	13
Protocol amendments	#25	Plans for communicating important protocol modifications (eg, changes to eligibility criteria, outcomes, analyses) to relevant parties (eg, investigators, REC / IRBs, trial participants, trial registries, journals, regulators)	13
Consent or assent	#26a	Who will obtain informed consent or assent from potential trial participants or authorised surrogates, and how (see Item 32)	13, and in protocols A, B and C
Consent or assent: ancillary studies	#26b	Additional consent provisions for collection and use of participant data and biological specimens in ancillary studies, if applicable	13, and in protocols A, B and C
Confidentiality	#27	How personal information about potential and enrolled participants will be collected, shared, and maintained in order to protect confidentiality before, during, and after the trial	Details in protocols A, B and C
Declaration of interests	#28	Financial and other competing interests for principal investigators for the overall trial and each study site	16
Data access	#29	Statement of who will have access to the final trial dataset, and disclosure of contractual agreements that limit such access for investigators	n/a
Ancillary and post trial care	#30	Provisions, if any, for ancillary and post-trial care, and for compensation to those who suffer harm from trial participation	Details in supplementary files for protocols A, B and C
Dissemination policy: trial results	#31a	Plans for investigators and sponsor to communicate trial results to participants, healthcare professionals, the public, and other relevant groups (eg, via publication, reporting in results databases, or other data sharing arrangements), including any publication restrictions	2, 14

For peer review only

Dissemination policy: authorship	#31b	Authorship eligibility guidelines and any intended use of professional writers	n/a
Dissemination policy: reproducible research	#31c	Plans, if any, for granting public access to the full protocol, participant-level dataset, and statistical code	14

Appendices

Informed consent materials	#32	Model consent form and other related documentation given to participants and authorised surrogates	n/a
Biological specimens	#33	Plans for collection, laboratory evaluation, and storage of biological specimens for genetic or molecular analysis in the current trial and for future use in ancillary studies, if applicable	n/a

None The SPIRIT checklist is distributed under the terms of the Creative Commons Attribution License CC-BY-ND 3.0. This checklist can be completed online using https://www.goodreports.org/, a tool made by the EQUATOR Network in collaboration with Penelope.ai

For peer review only

BMJ Open

Population differences in vaccine responses (POPVAC): scientific rationale and cross-cutting analyses for three linked, randomised controlled trials assessing the role, reversibility and mediators of immunomodulation by chronic infections in the tropics

Journal:	*BMJ Open*
Manuscript ID	bmjopen-2020-040425.R1
Article Type:	Protocol
Date Submitted by the Author:	01-Oct-2020
Complete List of Authors:	Nkurunungi, Gyaviira; MRC/UVRI and LSHTM Uganda Research Unit, Immunomodulation and Vaccines Programme<br>Zirimenya , Ludoviko ; MRC/UVRI and LSHTM Uganda Research Unit, Immunomodulation and Vaccines Programme<br>Natukunda , Agnes ; MRC/UVRI and LSHTM Uganda Research Unit, Immunomodulation and Vaccines Programme<br>Nassuuna , Jacent; MRC/UVRI and LSHTM Uganda Research Unit, Immunomodulation and Vaccines Programme<br>Oduru, Gloria; MRC/UVRI and LSHTM Uganda Research Unit, Immunomodulation and Vaccines Programme<br>Ninsiima , Caroline ; MRC/UVRI and LSHTM Uganda Research Unit, Immunomodulation and Vaccines Programme<br>Zziwa , Christopher ; MRC/UVRI and LSHTM Uganda Research Unit, Immunomodulation and Vaccines Programme<br>Akello , Florence ; MRC/UVRI and LSHTM Uganda Research Unit, Immunomodulation and Vaccines Programme<br>Kizindo , Robert ; MRC/UVRI and LSHTM Uganda Research Unit, Immunomodulation and Vaccines Programme<br>Akello, Mirriam; MRC/UVRI and LSHTM Uganda Research Unit, Immunomodulation and Vaccines Programme<br>Kaleebu, Pontiano; MRC/UVRI and LSHTM Uganda Research Unit<br>Wajja, Anne; MRC/UVRI and LSHTM Uganda Research Unit, Immunomodulation and Vaccines Programme<br>Luzze, Henry; Republic of Uganda Ministry of Health, National Expanded Program on Immunisation<br>Cose , Stephen ; MRC/UVRI and LSHTM Uganda Research Unit, Immunomodulation and Vaccines Programme; London School of Hygiene and Tropical Medicine Department of Clinical Research<br>Webb, Emily; London School of Hygiene & Tropical Medicine, MRC Tropical Epidemiology Group, Department of Infectious Disease Epidemiology,<br>Elliott, Alison ; MRC/UVRI and LSHTM Uganda Research Unit, Immunomodulation and Vaccines Programme; London School of Hygiene and Tropical Medicine Department of Clinical Research
**Primary Subject Heading**:	Infectious diseases

Secondary Subject Heading:	Immunology (including allergy), Infectious diseases, Public health, Epidemiology
Keywords:	Infection control < INFECTIOUS DISEASES, PARASITOLOGY, PUBLIC HEALTH, Immunology < TROPICAL MEDICINE, Epidemiology < TROPICAL MEDICINE, Paediatric infectious disease & immunisation < PAEDIATRICS

SCHOLARONE™
Manuscripts

I, the Submitting Author has the right to grant and does grant on behalf of all authors of the Work (as defined in the below author licence), an exclusive licence and/or a non-exclusive licence for contributions from authors who are: i) UK Crown employees; ii) where BMJ has agreed a CC-BY licence shall apply, and/or iii) in accordance with the terms applicable for US Federal Government officers or employees acting as part of their official duties; on a worldwide, perpetual, irrevocable, royalty-free basis to BMJ Publishing Group Ltd ("BMJ") its licensees and where the relevant Journal is co-owned by BMJ to the co-owners of the Journal, to publish the Work in this journal and any other BMJ products and to exploit all rights, as set out in our licence.

The Submitting Author accepts and understands that any supply made under these terms is made by BMJ to the Submitting Author unless you are acting as an employee on behalf of your employer or a postgraduate student of an affiliated institution which is paying any applicable article publishing charge ("APC") for Open Access articles. Where the Submitting Author wishes to make the Work available on an Open Access basis (and intends to pay the relevant APC), the terms of reuse of such Open Access shall be governed by a Creative Commons licence – details of these licences and which Creative Commons licence will apply to this Work are set out in our licence referred to above.

Other than as permitted in any relevant BMJ Author's Self Archiving Policies, I confirm this Work has not been accepted for publication elsewhere, is not being considered for publication elsewhere and does not duplicate material already published. I confirm all authors consent to publication of this Work and authorise the granting of this licence.

For peer review only

Population differences in vaccine responses (POPVAC): scientific rationale and cross-cutting analyses for three linked, randomised controlled trials assessing the role, reversibility and mediators of immunomodulation by chronic infections in the tropics

Gyaviira Nkurunungi[1,¶,*], Ludoviko Zirimenya[1,¶], Agnes Natukunda[1,¶], Jacent Nassuuna[1], Gloria Oduru[1], Caroline Ninsiima[1], Christopher Zziwa[1], Florence Akello[1], Robert Kizindo[1], Mirriam Akello[1], Pontiano Kaleebu[1], Anne Wajja[1], Henry Luzze[2], Stephen Cose[1,3], Emily L Webb[4], Alison M Elliott[1,5] **for the POPVAC trial team**

[1]Immunomodulation and Vaccines Programme, Medical Research Council/Uganda Virus Research Institute and London School of Hygiene and Tropical Medicine (MRC/UVRI and LSHTM) Uganda Research Unit, Entebbe, Uganda

[2]Uganda National Expanded Program on Immunisation, Ministry of Health, Kampala, Uganda

[3]Department of Clinical Research, London School of Hygiene and Tropical Medicine, London, United Kingdom

[4]MRC Tropical Epidemiology Group, Department of Infectious Disease Epidemiology, London School of Hygiene and Tropical Medicine, London, United Kingdom

¶These authors contributed equally

***Correspondence:** Gyaviira Nkurunungi; Gyaviira.Nkurunungi@mrcuganda.org

For peer review only - http://bmjopen.bmj.com/site/about/guidelines.xhtml

Abstract

Introduction

Vaccine-specific immune responses vary between populations and are often impaired in low-income, rural settings. Drivers of these differences are not fully elucidated, hampering identification of strategies for optimising vaccine effectiveness. We hypothesise that urban-rural (and regional and international) differences in vaccine responses are mediated to an important extent by differential exposure to chronic infections, particularly parasitic infections.

Methods and analysis

Three related trials sharing core elements of study design and procedures (allowing comparison of outcomes across the trials) will test the effects of 1) individually randomised intervention against schistosomiasis (Trial A) and malaria (Trial B), and 2) BCG re-vaccination (Trial C), on a common set of vaccine responses. We will enrol adolescents from Ugandan schools in rural high-schistosomiasis (Trial A) and rural high-malaria (Trial B) settings, and from an established urban birth cohort (Trial C). All participants will receive BCG on day 'zero'; yellow fever, oral typhoid and Human Papilloma Virus (HPV) vaccines at week 4; and HPV and Tetanus/diphtheria booster vaccine at week 28. Primary outcomes are BCG-specific IFN-γ responses (eight weeks after BCG) and for other vaccines, antibody responses to key vaccine antigens at four weeks after immunisation. Secondary analyses will determine effects of interventions on correlates of protective immunity, vaccine response waning, priming versus boosting immunisations, and parasite infection status and intensity. Overarching analyses will compare outcomes between the three trial settings. Sample archives will offer opportunities for exploratory evaluation of the role of immunological and 'trans-kingdom' mediators in parasite modulation of vaccine-specific responses.

Ethics and dissemination

Ethics approval has been obtained from relevant Ugandan and UK ethics committees. Results will be shared with Uganda Ministry of Health, relevant district councils, community leaders and study participants. Further dissemination will be done through conference proceedings and publications.

Trial registration

Current Controlled Trials identifiers: Trial A, ISRCTN60517191; Trial B, ISRCTN62041885; Trial C, ISRCTN10482904.

Article summary

For peer review only - http://bmjopen.bmj.com/site/about/guidelines.xhtml

Strengths and limitations of this study

- This will be the first well-powered programme of work to investigate effects of schistosomiasis treatment, of malaria treatment, and of BCG revaccination on vaccine responses in adolescents.
- A major strength of this work is the opportunity to synthesise findings from three different study settings with differential parasite exposure using causal mediation analysis to obtain a comprehensive understanding of how parasitic infections influence vaccine responses in human populations.
- The results will provide insight into effects of parasites on infectious disease susceptibility: immunisation, notably with live vaccines, offers a surrogate for infection challenge in human subjects.
- The sample archives developed will provide a major asset for exploration of new leads arising from this hypothesis-driven work, or for an alternative, "systems biology" approach investigating (for example) transcriptome, microbiome and virome.
- One limitation is that observational analyses of parasite effects are subject to potential unmeasured confounding; this will be mitigated by cautious interpretation of results and our intervention studies will address causality rigorously.

Word count

4098

Keywords

Vaccine; parasite; BCG; malaria; schistosomiasis

For peer review only

Introduction

Population differences in vaccine responses

Effective vaccines are key weapons against infectious diseases,[1] but are still lacking for many poverty-related, neglected, emerging and re-emerging infections. Vaccine responses vary between populations and are often impaired in low-income, rural settings.[2-6] A notable example is Bacillus Calmette–Guérin (BCG): both vaccine response and efficacy against tuberculosis differ internationally[3 4] and regionally.[6 7] Among other vaccines, yellow fever vaccine induced lower neutralising antibody levels, and responses waned faster, in Uganda compared to Switzerland.[5] Oral rotavirus and polio vaccines, are also affected.[2] Within-country, influenza and tetanus responses differed between urban and rural Gabon.[8 9] Responses to candidate tuberculosis,[10] malaria[11] and Ebola[12] vaccines are lower in Africa than in Europe or America. Prior exposure to the pathogen targeted by the vaccine, or to related organisms, may contribute to this phenomenon, but recent analyses implicate broader "environmental sensitisation",[6] the drivers of which have not been determined. Prior exposure cannot explain results for vaccines against rare organisms, such as Ebola. Thus, drivers of population differences in vaccine response are not fully elucidated; improved understanding is important for effective vaccine development and implementation.

The POPulation differences in VACcine responses (POPVAC) programme comprises three trials, A, B and C, designed to address this challenge. The individual trial protocols are presented separately in this journal (bmjopen-2020-040426, bmjopen-2020-040427 and bmjopen-2020-040430). This "Protocol X" provides an overview of our hypotheses and objectives.

Immunomodulation by parasitic infections

Parasitic infections are important in tropical low-income countries (LICs),[13-15] and long proposed as modulators of vaccine responses.[16-19] As detailed in POPVAC A (bmjopen-2020-040426) and POPVAC B (bmjopen-2020-040427) protocols, animal models[20-22] and observational human studies[23-25] support this hypothesis, but no well-powered trials have been conducted to evaluate causality and reversibility of parasite effects on vaccine responses in adolescents or adults.[26]

Trained immunity

Exposure to other unrelated infections or microbial antigens may also contribute to "environmental sensitisation", modulating vaccine responses through training of the innate immune system.[27] BCG immunisation is a key model for this effect,[28 29] as considered in Protocol C (bmjopen-2020-040430).

For peer review only - http://bmjopen.bmj.com/site/about/guidelines.xhtml

The "trans-kingdom" concept

The "trans-kingdom" concept[30] emphasises that mammals support a complex ecosystem of multicellular organisms, such as helminths, as well as bacteria, fungi, protozoa and viruses, and suggests that these interact in their effects on the mammalian immune system, rather than acting alone, as individual agents.[30] For example, in a mouse model, infection with the gut helminth *Heligmosomoides polygyrus*, or exposure to schistosome eggs, activated latent herpesvirus infection via alternative macrophage activation and the IL-4/Stat6 pathway.[31] In a contrasting study, *H. polygyrus* caused enhanced responses to Respiratory Syncytial Virus (RSV) in the mouse lung through interaction with the gut microbiome, translocation of microbial products to the circulation and enhanced systemic type I interferon expression.[32] Interestingly, in these studies, the same helminth resulted in opposite outcomes for latent viruses (for which it induced activation), versus exogenous viruses (for which it improved control).

Little has been done to evaluate these phenomena in human populations but, regarding latent herpesviruses our studies show that parasite exposure associates with elevated Kaposi's Sarcoma Herpesvirus antibody prevalence and titre (indicating viral activation).[33-35] The impact of malaria on Epstein Barr Virus (EBV), promoting induction of Burkitt's lymphoma, is well recognised.[36] Cytomegalovirus (CMV) has major immunological effects, including impact on vaccine responses.[37]

In humans, evidence of enhanced microbial translocation (MT) has been found during *S. mansoni*,[38] hookworm[39] and *Strongyloides*[40] infection, with altered expression of parameters such as toll-like receptor expression, but without the level of immune activation associated with septic shock or with MT in HIV infection. Nevertheless, the second mouse model discussed above shows that this may have profound effects on responses to infectious agents at remote sites.[32]

Thus, herpesvirus activation and, or, MT may mediate, in part, parasite-induced modulation of vaccine responses.

Differences in immunological characteristics between populations

Whatever the key exposures and mechanisms involved, immunological characteristics differ markedly between populations internationally,[5 41 42] and between urban and rural settings.[41 43 44] Characteristics that differ include gene methylation and expression (not solely attributable to population genetics);[43 45] responses to innate stimuli;[42 45] frequency and activation of innate immune cells, T and B cells, and memory cell pools.[5 41] Understanding the immunological predictors of vaccine response, and factors that drive them, will contribute to strategies for improving vaccine efficacy for rural, tropical settings.

For peer review only - http://bmjopen.bmj.com/site/about/guidelines.xhtml

Hypothesis and objectives

The overarching goal of the 'POPulation differences in VACcine responses' programme (POPVAC) is to understand population differences in vaccine responses, in order to identify strategies through which vaccine effectiveness can be optimised for low-income, tropical settings where they are especially needed. We focus on the hypothesis (**Figure 1**) that *geographical differences in vaccine responses are mediated to an important extent by differential exposure to chronic, particularly parasitic, infections; that parasites act in part via "trans-kingdom" effects; and that these exposures impact the pre-immunisation immune profile and hence vaccine response (and efficacy)"*.

We will address this hypothesis in three, linked trials (POPVAC A, B and C; detailed protocols published separately in this journal: bmjopen-2020-040426, bmjopen-2020-040427 and bmjopen-2020-040430, respectively) which share core elements of study design and procedures, allowing comparison of outcomes across the trials. Each trial will test effects of a different randomised intervention on a common set of vaccine responses. **POPVAC A** will determine the effect of intensive schistosomiasis treatment on vaccine responses among rural island adolescents. **POPVAC B** will determine the effect of intensive malaria treatment on vaccine responses among rural adolescents. **POPVAC C** will determine the effect of BCG re-vaccination on responses to unrelated vaccines.

This paper describes background and methods common to all three trials, summarises objectives linking the trials, and details planned approaches to cross-cutting objectives which will use data and samples from all trials. SPIRIT reporting guidelines[46] are used.

Our objectives are to

i. Determine whether there are reversible effects of chronic parasitic infection on vaccine response (**POPVAC A and B**).
ii. Determine whether BCG "pre-immunisation" enhances responses to unrelated vaccines (**POPVAC C**).
iii. Determine which life-course exposures influence vaccine responses in adolescence (using data from **POPVAC C**).
iv. Compare vaccine response profiles between three Ugandan settings (using data from all Trials): rural, high schistosomiasis exposure; rural, high malaria exposure; and urban.
v. Explore the role of "trans-Kingdom" interactions in determining vaccine responses (using samples and data from all Trials).
vi. Investigate pre-immunisation immunological parameters associated with vaccine responses and determine whether these are driven by parasite or microbial exposure (using samples and data from all Trials).

For peer review only - http://bmjopen.bmj.com/site/about/guidelines.xhtml

Methods and analysis

Setting

Uganda still experiences high schistosomiasis[47 48] and malaria burdens.[49-51] Our study sites (**Figure 2**) will be Lake Victoria Koome Islands (high-schistosomiasis),[47 52] Jinja district rural sub-counties (high-malaria)[51] and Entebbe (urban; low-schistosomiasis and low-malaria).[52] These settings provide ideal opportunities to investigate effects of schistosomiasis and malaria on vaccine responses. Geohelminths are less common in our settings:[47 53] hookworm, especially, has declined dramatically following government intervention programmes; therefore geohelminths will be considered as potential confounders, where appropriate, but not prioritised.

Cohorts

This work will involve adolescents from two rural settings and one urban cohort (**Figure 3**). Rural trials will recruit participants aged 9 to 17 years from primary schools in Koome islands (POPVAC A) and Jinja district (POPVAC B), selected purposefully to assure schistosomiasis and malaria prevalence appropriate to our design. This will allow us to investigate our two major infections separately, averting the risk that a potent effect of one infection masks an impact of the other, or of its treatment.

POPVAC C will recruit members of the Entebbe Mother and Baby Study (EMaBS) birth cohort:[54] 2500 women were recruited between 2003 and 2005 in a trial of anthelminthic treatment during pregnancy, investigating effects on infants' vaccine responses.[54] Children from the EMaBS birth cohort will be aged 13 to 17 years during recruitment to this study; about 300 individuals are expected to take part.

Interventions

In the high-schistosomiasis cohort (Trial A) we will individually randomise participants to intensive or standard praziquantel (PZQ) treatment, in a 1:1 ratio, in an open-label, parallel group trial.

In the high-malaria cohort (Trial B) we will individually randomise participants to monthly dihydroartemisinin-piperaquine (DP) versus placebo in a double-blind, placebo-controlled trial.

In the urban (EMaBS) cohort (Trial C) we will individually randomise participants to BCG re-vaccination, or no BCG re-vaccination, as the first component of the vaccine schedule, in a controlled, open-label, parallel group trial.

For peer review only

Recruitment criteria, interventions, randomisation and treatment allocation procedures are detailed in protocols for Trials A, B and C (bmjopen-2020-040426, bmjopen-2020-040427 and bmjopen-2020-040430, respectively; published in this journal).

Immunisations

We have previously highlighted the complexity of helminth effects on vaccine responses.[26] Differences in parasite effects on live and non-live, oral and parenteral, priming and boosting vaccines may contribute to this. Activated innate responses may kill live vaccines and suppress subsequent adaptive responses,[5 55] but bias, or enhance, responses to toxoids or proteins;[17 23 56] intestinal inflammation may impair responses to live oral vaccines;[57] priming may be more vulnerable than boosting.[58 59] Thus, results from a single-vaccine study would not be generalisable.

Therefore, we will study a portfolio of licensed vaccines expected to be beneficial (some already given) to adolescents in Uganda: live parenteral (BCG, yellow fever), live oral (typhoid) and non-live (HPV [a viral particle vaccine] and tetanus/diphtheria [Td; toxoid vaccines]). This will allow us to compare effects of interventions and exposures between vaccine types. Each cohort will receive the same vaccine portfolio (**Table 1**), comprising three main immunisation days (weeks 0, 4 and 28). Additional HPV immunisation will be provided for girls aged 14 years or above, and a second tetanus/diphtheria boost will be given after study completion, to accord with national Expanded Programme on Immunisation (EPI) routines, but responses to these will not specifically be addressed. Further rationale for vaccine selection is detailed in Supplementary information. Our schedule has been developed in consultation with the EPI programme (Table S1) and is cognizant of potential interference between vaccines (Table S2, Supplementary information).

Although optimal timings for outcome measures vary between vaccines, sampling (for primary endpoints) will be done at eight weeks post BCG, four weeks post YF-17D, Ty21a, HPV and Td (**Figure 4**), targeting establishment of memory responses and antibody response peaks. A secondary endpoint at one year will assess waning. Analyses will take baseline measurements into account.

Table 1. Immunisation schedule

	**Immunisation week 0**	**Immunisation week 4**	**[Immunisation week 8]**	**Immunisation week 28**	**[Immunisation week 52]**
Live vaccines	BCG vaccination / re-vaccination[1,2,3]	Yellow fever (YF-17D)<br>Oral typhoid (Ty21a)			
Non-live vaccines		HPV prime	HPV boost for girls aged ≥14 years[4,5]	HPV boost and Tetanus/ diphtheria boost	Tetanus/ diphtheria boost[5,6]

1. All participants in the urban (Entebbe Mother and Baby Study; EMaBS) cohort received BCG at birth, so within EMaBS this will be revaccination; prior BCG status may vary for the rural cohorts (data on history and documentation of prior BCG, and presence of a BCG scar,

For peer review only - http://bmjopen.bmj.com/site/about/guidelines.xhtml

will be documented although these approaches have limitations for determining BCG status)
2. EMaBS participants will be randomised to receive BCG "pre-immunisation" or not as part of Trial C
3. All participants in rural cohorts will receive BCG (Trials A and B)
4. The National EPI programme recommends three doses of HPV vaccine for older girls
5. These doses will be given to comply with guidelines but outcomes specifically relating to these doses will not be assessed
6. Priming by immunisation in infancy is assumed

Outcomes

Primary outcomes, assessed in all participants, will be:

i. **BCG:** *BCG-specific IFN-γ ELISpot response* eight weeks post BCG immunisation: this response is associated with decreased risk of tuberculosis disease post-immunisation in infants.[60]
ii. **YF-17D:** neutralising antibody titres (plaque-reduction neutralisation test) four weeks post YF immunisation.
iii. **Ty21a:** *Salmonella typhi* lipopolysaccharide (LPS)-specific immunoglobulin (Ig)G concentration four weeks post Ty21a immunisation.
iv. **HPV:** IgG specific for L1-proteins of HPV-16/18 four weeks post HPV priming immunisation.
v. **Td:** tetanus and diphtheria toxoid-specific IgG concentration four weeks post Td immunisation.

Secondary outcomes, assessed in all participants, will further investigate estimates of protective immunity (for vaccines where these are available) and dynamics of the vaccine responses, as well as the impact of interventions on parasite clearance.

i. **Protective immunity.** Proportions with protective neutralising antibody (YF); protective IgG levels (TT);[61] seroconversion rates (Ty21a) four weeks post immunisation.
ii. **Response waning.** Primary outcome measures (all vaccines) repeated at week 52, and area-under-the curve analyses. Parasites may accelerate,[58] and interventions delay, waning.
iii. **Priming versus boosting.** Effects on priming versus boosting will be examined for HPV, comparing outcomes four weeks after the first and second vaccine doses.

Our sample collection will offer opportunities for an array of exploratory immunological evaluations, focusing on vaccine antigen specific outcomes. Exploratory assessments will provide detail on immune response characteristics over the study time-course, and on the role of immunological profiles and trans-kingdom effects in mediating modulation of vaccine-specific responses.

Sample size

Our sample size estimates focus on primary outcomes for the main comparisons for objectives i, ii and iv.

Based on literature,[5 60 62] we anticipate standard deviations (SDs) of primary outcome measures lying between 0.3 and 0.6 $\log_{10}$; responses in rural, high-parasite settings 0.3-0.4 $\log_{10}$ smaller than in

For peer review only

the urban setting,[63 64] and effective treatment restoring responses by approximately 0.2 $\log_{10}$.[63] We therefore power our study to detect differences of this magnitude (0.2 $\log_{10}$) or smaller. We assume *S. mansoni* prevalence of $\geq$80% in the high-schistosomiasis setting[47] and malaria infection prevalence of $\geq$60% in the high-malaria setting.

Our planned sample sizes are as follows:

High-schistosomiasis setting (objective i, Trial A): 480 (240 quarterly PZQ, 240 annual PZQ); of whom we anticipate 384 will be *S. mansoni* infected, giving 192 participants in each trial arm with *S. mansoni* infection at baseline.

High-malaria setting (objective i, Trial B): 640 (320 DP, 320 placebo) of whom we anticipate 384 will be malaria infected, giving 192 participants with malaria in each trial arm at baseline.

Urban setting (objective ii, Trial C): 300 EMaBS participants (150 BCG “pre-immunisation”, 150 no BCG immunisation).

Urban vs rural and urban vs island comparisons (objective iv): 150 urban EMaBS, 240 rural high-schistosomiasis control group participants and 320 rural high-malaria control group participants will be included. Allowing 20% loss to follow-up in rural cohorts and 10% in the EMaBS cohort, this will give >80% power to detect a difference of 0.14$\log_{10}$ or more in vaccine responses in urban compared to each rural setting at 5% significance level assuming vaccine response standard deviation of 0.4$\log_{10}$.

Table 2 shows power estimates for objective i, ii and iv. Sample size considerations for additional analyses are detailed in the protocol papers for the individual trials (bmjopen-2020-040426, bmjopen-2020-040427 and bmjopen-2020-040430).

Table 2. Power estimates for objectives i, ii and iv (5% significance level)

Standard deviation ($\log_{10}$)	Log_{10} difference 0.08	0.10	0.12	0.14	0.16	0.18	0.20
Objective i: 192 high-intensity vs 192 low-intensity (infected only)							
0.3	65%	83%	94%	98%	>99%	>99%	>99%
0.4	42%	59%	75%	87%	94%	98%	99%
0.5	29%	42%	56%	69%	80%	88%	94%
0.6	21%	31%	42%	53%	65%	75%	83%
Objective ii: 150 BCG “pre-immunisation” vs 150 no BCG vaccination							
0.3	59%	78%	91%	97%	99%	>99%	>99%
0.4	37%	53%	69%	82%	91%	96%	98%
0.5	26%	37%	50%	63%	75%	84%	91%
0.6	19%	28%	37%	48%	59%	69%	78%
Objective iv: 240 rural vs 150 urban[1]							
0.3	66%	84%	94%	99%	>99%	>99%	>99%
0.4	43%	60%	76%	87%	94%	98%	99%
0.5	30%	43%	57%	80%	81%	89%	94%
0.6	22%	32%	43%	54%	66%	76%	84%

For peer review only

Cells highlighted in grey correspond to >80% power.
[1]Numbers shown for rural high-schistosomiasis versus urban setting. Power will be greater for rural high-malaria vs urban setting

Approach to trial objectives

Approaches to objective i (trials A and B) and objective ii (trial C) are detailed in the focused papers for these trials (bmjopen-2020-040426, bmjopen-2020-040427 and bmjopen-2020-040430). Here we present approaches to objectives iii, iv, v and vi.

Objective iii. Life-course exposures that influence vaccine responses in adolescence (Trial C)

Data collected in the EMaBS over the participants' life course (and as part of this protocol) will be used in regression analyses to investigate associations between infection exposure *in utero*, infancy, childhood, and adolescence on vaccine responses. Socio-demographic variables will be considered as potential confounders. We will include the following important variables: age, sex, sociodemographic variables, BCG strain received at birth (and other vaccines received), helminth-related exposures, malaria and documented illness events.

Regression analyses will be used to evaluate associations between infections and outcomes, with adjustment for confounders. We will use a hierarchical statistical modelling approach, so that for analysis of early life exposures we will not adjust for later life exposures that may be on the causal pathway, but for associations between later childhood or current exposures and vaccine responses we will adjust for early life exposures as potential confounders. Linear regression will be used for the primary outcomes and for continuous secondary outcomes. Outcome distributions are likely to be positively skewed; where necessary, we will apply log transformations to normalise outcome distributions before linear regression analysis. Logistic regression will be used for the protective immunity secondary outcomes, which are binary. We shall also investigate whether multiple infection exposures combine multiplicatively in their effect, and test for interaction.

Genetic factors will also be considered: genetic data is already available for EMaBS based on earlier approvals for work on genetic polymorphisms and vaccine responses in infancy. It will be of interest to determine whether genetic factors also have a strong influence on adolescents' responses.

Objective iv. Urban-rural comparisons in vaccine response

We hypothesise that environmental (especially parasite) exposures are key drivers of population differences in vaccine response. Hence we predict differences between urban and rural settings within Uganda (with urban vaccine responses stronger, as observed in Gabon[8 9] and Senegal[64]) and that these differences will be related to parasite exposure.

For peer review only - http://bmjopen.bmj.com/site/about/guidelines.xhtml

The key exposure for this objective is ‘setting’. We will compare outcomes between urban EMaBS and (i) rural high-schistosomiasis and (ii) rural high-malaria participants. For these comparisons we shall include in the analysis only the urban EMaBS participants who were randomised to receive BCG “pre-immunisation,” such that their immunisation schedule is identical to that in the other two settings. We will include only control groups from the rural high-schistosomiasis and high-malaria settings, since these will have received minimal anti-parasite treatment (**Figures 3** and **4**). In the primary analysis we will adjust for age and sex, but not for factors likely to be on the causal pathway between setting and vaccine response.

Although our settings are purposely chosen based on parasite prevalence, there will be overlap in this, and other, exposures. Therefore we will undertake exploratory analyses using causal mediation modelling, a statistical approach which aims to identify factors that mediate an observed association [65] (here, between setting and vaccine response). We will focus on current *S. mansoni* and *P. falciparum* infection, and prior exposure (assessed by anti-schistosome and anti-malaria antibody), as key potential mediators of interest (Figure S1). Explanatory factors for urban versus rural setting, and for vaccine responses, will be included in the causal diagram and adjusted for in analyses. Although nutrition may impact vaccine responses,[66] we expect this to be less important in healthy adolescents than might be the case in young children; however, data on anthropometric parameters and diet will be collected and adjusted for.

One limitation is that observational analyses of parasite effects are beset with potential unmeasured confounding factors. Results will be interpreted cautiously and Objective i (Trials A and B) will address causality rigorously. The sample archive will allow future investigation of additional potential mediators. Another potential limitation is that in Uganda, rural to urban migration for schooling is common. However, in the EMaBS birth cohort we have data on residence. Urban to rural migration for schooling is relatively unlikely but will be documented.

With Objective iv we expect to confirm differences in vaccine responses between settings; and to obtain insights into environmental factors that mediate this. By addressing the portfolio of vaccines and array of outcomes presented above, we will identify categories of vaccine most affected; we will distinguish effects on waning and (for HPV) on priming versus boosting. Vaccine response data from urban EMaBS adolescents will provide a reference, suggesting the extent to which changes in infection exposure and lifestyle are likely to influence responses to vaccines (and to infectious diseases) as Africa’s urbanisation advances.

For peer review only

For peer review only - http://bmjopen.bmj.com/site/about/guidelines.xhtml

331 *Objective v. The role of "trans-Kingdom" interactions in determining vaccine responses*

332 We will address the hypothesis that parasites also impact vaccine responses through "trans-
333 kingdom" effects, mediated by other components of the host ecosystem. We propose that
334 herpesvirus activation and microbial translocation are likely to be determinants of vaccine response,
335 and they are driven by parasitic infections. Samples from selected participants from each setting and
336 study arm will be examined. To optimise the precision of our comparisons these will be selected
337 among those (for the high-schistosomiasis and high-malaria settings) who had the parasitic infection
338 of interest at baseline (and, if possible, no other parasitic infection detected) and complied with the
339 intended treatment. To test our hypothesis, we will measure markers of viral activation and
340 microbial translocation in plasma/serum and/or stool. Initial analyses (**Figure 5**) will investigate
341 associations between these markers and vaccine responses (arrow F); and between settings (arrow
342 D) or parasite infection and treatment (arrow E) and viral activation or microbial translocation.
343 Objective vi will link herpesvirus activation, microbial translocation and parasitic infections to
344 immune activation and regulation.

345 *Objective vi. Pre-immunisation immunological parameters*

346 There is strong evidence that pre-immunisation immunological status impacts vaccine responses[5 60]
347 but the factors that determine this status have not been identified. We hypothesise that the
348 environmental, parasitic, viral and microbial exposures addressed in Objectives i-v are key (**Figure 5**).
349 We aim to investigate this by identifying immunological parameters that specifically link the distal
350 exposures to vaccine response. These include the circulating cytokine and chemokine milieu, innate
351 cell responses (which govern adaptive responses), and frequencies and phenotypes of both innate
352 and adaptive cells. Our principal focus will be on pre-immunisation measurements although, in
353 selected groups of most interest, it will also be possible to examine samples obtained post-
354 immunisation to identify which biomarkers or cell populations change.

355 Initial analyses will confirm hypothesised, and identify new, pre-immunisation immunological
356 parameters associated with responses to vaccines in our portfolio (**Figure 5**, arrow J). If we observe
357 associations between rural vs urban location (arrow B) or parasites and their treatment (arrow C)
358 and vaccines responses, we will explore whether these effects are mediated by viral and microbial
359 translocation variables and pre-immunisation immune profiles using causal mediation analyses.[65]

360 ***Operational considerations***

For peer review only - http://bmjopen.bmj.com/site/about/guidelines.xhtml

A Programme Steering Committee has been set up to guide progress across all projects. A data and safety monitoring board (DSMB) has also been appointed to provide real-time safety oversight. Details of these and other operational activities can be found in Supplementary information.

Ethics and dissemination

Ethical and regulatory approval has been granted from the Research Ethics Committees of Uganda Virus Research Institute (UVRI REC) and the London School of Hygiene and Tropical Medicine (LSHTM), the Uganda National Council for Science and Technology (UNCST) and the Uganda National Drug Authority (NDA); details are given in Supplementary information and in each Trial paper. Given the importance of the data and sample archive as a resource for mechanistic studies on the determinants of vaccine responses, assent and consent processes will include storage of samples for future and genetic studies, and anonymised data and sample sharing. Any protocol amendments will be submitted to ethics committees and regulatory bodies for approval before implementation.

Study findings will be published through open access peer-reviewed journals, presentations at local, national and international conferences and to the local community through community meetings. Anonymised participant level datasets generated will be available upon request.

Patient and public involvement

Concepts involved in this work have been discussed with colleagues at the Vector Control Division and Expanded Programme on Immunisation in the Ministry of Health (Uganda) and with relevant District Councils, community leaders and Village Health Teams. We also have held meetings to explain the proposed work to teachers, parents, participants and village members, and to address their questions. Study findings will be shared with these stakeholders.

Data management and analysis

Socio-demographic information and clinical and laboratory measurements will be recorded and managed using REDCap (Research Electronic Data Capture) tools,[67 68] with paper-based forms as back-up. All data will be recorded under a unique study ID number. When paper forms must be used, data will be entered in a study-specific database, with standard checks for discrepancies. All data for analysis will be anonymised and stored on a secure and password-protected server, with access limited to essential research personnel. Anonymised participant level datasets generated will be available for sharing upon request.

For peer review only - http://bmjopen.bmj.com/site/about/guidelines.xhtml

Discussion

This will be the first well-powered set of studies to investigate effects of schistosomiasis and malaria treatment, and of BCG re-vaccination, on vaccine responses in adolescents. The results will add to understanding of population differences in vaccine responses and of interventions that may enhance them. The sample archives developed will provide a major asset for exploration of new leads arising from this hypothesis-driven work, or for an alternative, “systems biology” approach investigating (for example) transcriptome, microbiome and virome.

Our focus is on the immunological effects of infection exposure in human participants. By understanding these effects, we aim to inform and promote vaccine design tailored to the challenging environment of low-income countries, and to inform the development of public health strategies (such as tailored immunisation regimens and combined parasite-control/immunisation interventions) that will optimise vaccine implementation in parasite-endemic settings.

Our strong immunoepidemiological design and nested immunological studies will address specific hypotheses regarding pathways of effects. Population immunology is useful for translation of findings to and from basic (especially animal) studies to human health. Our randomised design will determine causal, and reversible, effects of parasitic infections. Substantial sample sizes are needed because immune responses are highly variable in human populations.

We have several reasons for studying vaccine responses particularly among adolescents. In this study setting, they bear a heavy parasite burden.[69] As well, this age-group is a target group for vaccines against tuberculosis and sexually transmitted infections (currently human papilloma virus [HPV] – in future, it is hoped, for vaccines against HIV) and for booster immunisations. Also, they enter a period of increased risk of pulmonary tuberculosis after the relatively low-risk period of mid-childhood, and are thus a target group for improved vaccines for tuberculosis.

Study timeline

POPVAC A began recruiting in July 2019. Intervention will be up to 12 months, with completion of the project scheduled for September 2020. POPVAC B is scheduled begin recruiting in February 2021. Intervention will be up to 12 months, with completion of the project scheduled for April 2022. POPVAC C is scheduled to begin recruiting in May 2020. Intervention will be up to 12 months, with completion of the project scheduled for April 2022.

For peer review only

For peer review only - http://bmjopen.bmj.com/site/about/guidelines.xhtml

Competing interests

Alison Elliott reports a grant from the Medical research Council, UK (POPVAC programme funding). The rest of the authors declare that they have no conflicts of interest.

Author contributions

AME conceived the study. AME, GN, ELW, AN, SC, LZ, JN, AW, PK and HL contributed to study design. LZ, GO, CN, CZ, and FA are site clinicians/nurses/clinical laboratory technicians providing valuable input on clinical considerations of the intervention. RK heads the team of field workers handling the organisational integration of the intervention. MA is the study internal monitor. AN and ELW are involved in organisation of the databases, trial randomization, treatment allocation and drawing up of analytical plans. GN, LZ, AN, SC, ELW and AME drafted the manuscript. All authors reviewed the manuscript, contributed to it and approved the final version.

Acknowledgements

We thank the Uganda National Expanded Programme for Immunisation, the Vector Control Division of the Ministry of Health, the Ministry of Education, Jinja district local government, Wakiso district local government and Entebbe hospital for their support. We thank members of the POPVAC programme steering committee (chaired by Prof. Richard Hayes) and the Data and Safety Monitoring Board (Dr David Meya, Prof. Andrew Prendergast and Dr Elizabeth George).

Funding

The POPVAC programme of work is supported by the Medical Research Council of the United Kingdom (grant number MR/R02118X/1). SC and JN are supported in part by the Makerere University – Uganda Virus Research Institute Centre of Excellence for Infection and Immunity Research and Training (MUII-plus). MUII-plus is funded under the DELTAS Africa Initiative. The DELTAS Africa Initiative is an independent funding scheme of the African Academy of Sciences (AAS), Alliance for Accelerating Excellence in Science in Africa (AESA) and supported by the New Partnership for Africa's Development Planning and Coordinating Agency (NEPAD Agency) with funding from the Wellcome Trust (grant 107743) and the UK Government. The MRC/UVRI and LSHTM Uganda Research Unit is jointly funded by the UK Medical Research Council (MRC) and the UK Department for International Development (DFID) under the MRC/DFID Concordat agreement and is also part of the EDCTP2 programme supported by the European Union.

The study sponsor (London School of Hygiene and Tropical Medicine) and funders had no role in study design; collection, management, analysis, and interpretation of data; writing of the protocol; and the decision to submit the protocol for publication.

For peer review only

POPVAC trial team

Principal investigator*:* Alison Elliott; ***Project leader***: Ludoviko Zirimenya; ***laboratory staff****:* Gyaviira Nkurunungi, Stephen Cose, Rebecca Amongin, Beatrice Nassanga, Jacent Nassuuna, Irene Nambuya, Prossy Kabuubi, Emmanuel Niwagaba, Gloria Oduru, Grace Kabami; ***statisticians and data managers****:* Emily Webb, Agnes Natukunda, Helen Akurut, Alex Mutebe; ***clinicians****:* Anne Wajja, Milly Namutebi, Christopher Zziwa; ***nurses****:* Caroline Onen, Esther Nakazibwe, Josephine Tumusiime, Caroline Ninsiima, Susan Amongi, Florence Akello; ***internal monitor****:* Mirriam Akello; ***field workers****:* Robert Kizindo, Moses Sewankambo, Denis Nsubuga, Samuel Kiwanuka, Fred Kiwudhu; ***boatman****:* David Abiriga; ***administrative management****:* Moses Kizza, Samsi Nansukusa; ***internal and external collaborators:*** Pontiano Kaleebu, Hermelijn Smits, Maria Yazdanbakhsh, Govert van Dam, Paul Corstjens, Sarah Staedke, Henry Luzze, James Kaweesa, Edridah Tukahebwa, Elly Tumushabe, Moses Muwanga

For peer review only

For peer review only - http://bmjopen.bmj.com/site/about/guidelines.xhtml

References

1. Koff WC, Burton DR, Johnson PR, et al. Accelerating next-generation vaccine development for global disease prevention. *Science* 2013;340(6136):1232910. doi: 10.1126/science.1232910

2. Taniuchi M, Platts-Mills JA, Begum S, et al. Impact of enterovirus and other enteric pathogens on oral polio and rotavirus vaccine performance in Bangladeshi infants. *Vaccine* 2016;34(27):3068-75. doi: 10.1016/j.vaccine.2016.04.080 [published Online First: 2016/05/08]

3. Fine PE. Variation in protection by BCG: implications of and for heterologous immunity. *Lancet* 1995;346(8986):1339-45.

4. Black GF, Weir RE, Floyd S, et al. BCG-induced increase in interferon-gamma response to mycobacterial antigens and efficacy of BCG vaccination in Malawi and the UK: two randomised controlled studies. *Lancet* 2002;359(9315):1393-401. doi: 10.1016/S0140-6736(02)08353-8

5. Muyanja E, Ssemaganda A, Ngauv P, et al. Immune activation alters cellular and humoral responses to yellow fever 17D vaccine. *The Journal of clinical investigation* 2014;124(7):3147-58. doi: 10.1172/jci75429 [published Online First: 2014/06/10]

6. Barreto ML, Pilger D, Pereira SM, et al. Causes of variation in BCG vaccine efficacy: examining evidence from the BCG REVAC cluster randomized trial to explore the masking and the blocking hypotheses. *Vaccine* 2014;32(30):3759-64. doi: 10.1016/j.vaccine.2014.05.042

7. Barreto ML, Pereira SM, Pilger D, et al. Evidence of an effect of BCG revaccination on incidence of tuberculosis in school-aged children in Brazil: second report of the BCG-REVAC cluster-randomised trial. *Vaccine* 2011;29(31):4875-7. doi: 10.1016/j.vaccine.2011.05.023

8. van Riet E, Adegnika AA, Retra K, et al. Cellular and humoral responses to influenza in gabonese children living in rural and semi-urban areas. *The Journal of infectious diseases* 2007;196(11):1671-8. doi: 10.1086/522010 [published Online First: 2007/11/17]

9. van Riet E, Retra K, Adegnika AA, et al. Cellular and humoral responses to tetanus vaccination in Gabonese children. *Vaccine* 2008;26(29-30):3690-5. doi: 10.1016/j.vaccine.2008.04.067 [published Online First: 2008/06/10]

10. Tanner R, Kakalacheva K, Miller E, et al. Serum indoleamine 2,3-dioxygenase activity is associated with reduced immunogenicity following vaccination with MVA85A. *BMC infectious diseases* 2014;14:660. doi: 10.1186/s12879-014-0660-7 [published Online First: 2014/12/04]

11. Sissoko MS, Healy SA, Katile A, et al. Safety and efficacy of PfSPZ Vaccine against Plasmodium falciparum via direct venous inoculation in healthy malaria-exposed adults in Mali: a randomised, double-blind phase 1

For peer review only - http://bmjopen.bmj.com/site/about/guidelines.xhtml

trial. *The Lancet Infectious diseases* 2017;17(5):498-509. doi: 10.1016/s1473-3099(17)30104-4 [published Online First: 2017/02/22]

12. Venkatraman N, Ndiaye BP, Bowyer G, et al. Safety and Immunogenicity of a Heterologous Prime-Boost Ebola Virus Vaccine Regimen in Healthy Adults in the United Kingdom and Senegal. *The Journal of infectious diseases* 2019;219(8):1187-97. doi: 10.1093/infdis/jiy639

13. Colley DG, Bustinduy AL, Secor WE, et al. Human schistosomiasis. *Lancet* 2014;383(9936):2253-64. doi: 10.1016/s0140-6736(13)61949-2 [published Online First: 2014/04/05]

14. Pullan RL, Smith JL, Jasrasaria R, et al. Global numbers of infection and disease burden of soil transmitted helminth infections in 2010. *Parasites & vectors* 2014;7:37. doi: 10.1186/1756-3305-7-37 [published Online First: 2014/01/23]

15. WHO. World Malaria Report. 2016

16. Crawley J, Sismanidis C, Goodman T, et al. Effect of intermittent preventive treatment for malaria during infancy on serological responses to measles and other vaccines used in the Expanded Programme on Immunization: results from five randomised controlled trials. *Lancet* 2012;380(9846):1001-10. doi: 10.1016/s0140-6736(12)60775-2 [published Online First: 2012/08/02]

17. Brown J, Baisley K, Kavishe B, et al. Impact of malaria and helminth infections on immunogenicity of the human papillomavirus-16/18 AS04-adjuvanted vaccine in Tanzania. *Vaccine* 2014;32(5):611-7. doi: 10.1016/j.vaccine.2013.11.061

18. Cunnington AJ, Riley EM. Suppression of vaccine responses by malaria: insignificant or overlooked? *Expert review of vaccines* 2010;9(4):409-29. doi: 10.1586/erv.10.16 [published Online First: 2010/04/08]

19. Bentwich Z, Kalinkovich A, Weisman Z, et al. Can eradication of helminthic infections change the face of AIDS and tuberculosis? *Immunol Today* 1999;20(11):485-7.

20. Elias D, Akuffo H, Pawlowski A, et al. Schistosoma mansoni infection reduces the protective efficacy of BCG vaccination against virulent Mycobacterium tuberculosis. *Vaccine* 2005;23(11):1326-34. doi: https://doi.org/10.1016/j.vaccine.2004.09.038

21. Laranjeiras RF, Brant LC, Lima AC, et al. Reduced protective effect of Plasmodium berghei immunization by concurrent Schistosoma mansoni infection. *Mem Inst Oswaldo Cruz* 2008;103(7):674-7. doi: 10.1590/s0074-02762008000700008 [published Online First: 2008/12/06]

22. Su Z, Segura M, Stevenson MM. Reduced protective efficacy of a blood-stage malaria vaccine by concurrent nematode infection. *Infection and immunity* 2006;74(4):2138-44. doi: 10.1128/iai.74.4.2138-2144.2006 [published Online First: 2006/03/23]

For peer review only

23. Sabin EA, Araujo MI, Carvalho EM, et al. Impairment of tetanus toxoid-specific Th1-like immune responses in humans infected with Schistosoma mansoni. *The Journal of infectious diseases* 1996;173(1):269-72. [published Online First: 1996/01/01]

24. Esen M, Mordmuller B, de Salazar PM, et al. Reduced antibody responses against Plasmodium falciparum vaccine candidate antigens in the presence of Trichuris trichiura. *Vaccine* 2012;30(52):7621-4. doi: 10.1016/j.vaccine.2012.10.026 [published Online First: 2012/10/23]

25. Kilian HD, Nielsen G. Cell-mediated and humoral immune response to tetanus vaccinations in onchocerciasis patients. *Trop Med Parasitol* 1989;40(3):285-91. [published Online First: 1989/09/01]

26. Sanya RE, Nkurunungi G, Andia Biraro I, et al. A life without worms. *Transactions of the Royal Society of Tropical Medicine and Hygiene* 2017:1-9. doi: 10.1093/trstmh/trx010 [published Online First: 2017/03/25]

27. Netea MG, Joosten LAB, Latz E, et al. Trained immunity: A program of innate immune memory in health and disease. *Science (New York, NY)* 2016;352(6284):aaf1098-aaf98. doi: 10.1126/science.aaf1098 [published Online First: 2016/04/21]

28. Blok BA, Arts RJ, van Crevel R, et al. Trained innate immunity as underlying mechanism for the long-term, nonspecific effects of vaccines. *J Leukoc Biol* 2015;98(3):347-56. doi: 10.1189/jlb.5RI0315-096R

29. Leentjens J, Kox M, Stokman R, et al. BCG Vaccination Enhances the Immunogenicity of Subsequent Influenza Vaccination in Healthy Volunteers: A Randomized, Placebo-Controlled Pilot Study. *The Journal of infectious diseases* 2015;212(12):1930-8. doi: 10.1093/infdis/jiv332

30. Pfeiffer JK, Virgin HW. Viral immunity. Transkingdom control of viral infection and immunity in the mammalian intestine. *Science* 2016;351(6270) doi: 10.1126/science.aad5872

31. Reese TA, Wakeman BS, Choi HS, et al. Helminth infection reactivates latent gamma-herpesvirus via cytokine competition at a viral promoter. *Science* 2014;345(6196):573-7. doi: 10.1126/science.1254517 [published Online First: 2014/06/28]

32. McFarlane AJ, McSorley HJ, Davidson DJ, et al. Enteric helminth-induced type I interferon signaling protects against pulmonary virus infection through interaction with the microbiota. *The Journal of allergy and clinical immunology* 2017 doi: 10.1016/j.jaci.2017.01.016 [published Online First: 2017/02/16]

33. Wakeham K, Johnston WT, Nalwoga A, et al. Trends in Kaposi's sarcoma-associated Herpesvirus antibodies prior to the development of HIV-associated Kaposi's sarcoma: a nested case-control study. *Int J Cancer* 2015;136(12):2822-30. doi: 10.1002/ijc.29329

For peer review only

For peer review only - http://bmjopen.bmj.com/site/about/guidelines.xhtml

34. Wakeham K, Webb EL, Sebina I, et al. Parasite infection is associated with Kaposi's sarcoma associated herpesvirus (KSHV) in Ugandan women. *Infectious agents and cancer* 2011;6(1):15. doi: 10.1186/1750-9378-6-15

35. Wakeham K, Webb EL, Sebina I, et al. Risk factors for seropositivity to Kaposi sarcoma-associated herpesvirus among children in Uganda. *Journal of acquired immune deficiency syndromes* 2013;63(2):228-33. doi: 10.1097/QAI.0b013e31828a7056

36. Moormann AM, Bailey JA. Malaria - how this parasitic infection aids and abets EBV-associated Burkitt lymphomagenesis. *Current opinion in virology* 2016;20:78-84. doi: 10.1016/j.coviro.2016.09.006 [published Online First: 2016/10/01]

37. Frasca D, Blomberg BB. Aging, cytomegalovirus (CMV) and influenza vaccine responses. *Human vaccines & immunotherapeutics* 2016;12(3):682-90. doi: 10.1080/21645515.2015.1105413 [published Online First: 2015/11/21]

38. Onguru D, Liang Y, Griffith Q, et al. Human schistosomiasis is associated with endotoxemia and Toll-like receptor 2- and 4-bearing B cells. *The American journal of tropical medicine and hygiene* 2011;84(2):321-4. doi: 10.4269/ajtmh.2011.10-0397 [published Online First: 2011/02/05]

39. George PJ, Anuradha R, Kumar NP, et al. Evidence of microbial translocation associated with perturbations in T cell and antigen-presenting cell homeostasis in hookworm infections. *PLoS neglected tropical diseases* 2012;6(10):e1830. doi: 10.1371/journal.pntd.0001830 [published Online First: 2012/10/12]

40. Rajamanickam A, Munisankar S, Bhootra Y, et al. Microbial Translocation Associated with an Acute-Phase Response and Elevations in MMP-1, HO-1, and Proinflammatory Cytokines in Strongyloides stercoralis Infection. *Infection and immunity* 2017;85(1) doi: 10.1128/iai.00772-16 [published Online First: 2016/11/09]

41. Mbow M, de Jong SE, Meurs L, et al. Changes in immunological profile as a function of urbanization and lifestyle. *Immunology* 2014;143(4):569-77. doi: 10.1111/imm.12335

42. Smolen KK, Ruck CE, Fortuno ES, 3rd, et al. Pattern recognition receptor-mediated cytokine response in infants across 4 continents. *The Journal of allergy and clinical immunology* 2014;133(3):818-26 e4. doi: 10.1016/j.jaci.2013.09.038

43. Idaghdour Y, Storey JD, Jadallah SJ, et al. A genome-wide gene expression signature of environmental geography in leukocytes of Moroccan Amazighs. *PLoS genetics* 2008;4(4):e1000052. doi: 10.1371/journal.pgen.1000052

44. Amoah AS, Obeng BB, May L, et al. Urban-rural differences in the gene expression profiles of Ghanaian children. *Genes and immunity* 2014;15(5):313-9. doi: 10.1038/gene.2014.21

For peer review only - http://bmjopen.bmj.com/site/about/guidelines.xhtml

45. Labuda LA, de Jong SE, Meurs L, et al. Differences in innate cytokine responses between European and African children. *PloS one* 2014;9(4):e95241. doi: 10.1371/journal.pone.0095241 [published Online First: 2014/04/20]

46. Chan AW, Tetzlaff JM, Altman DG, et al. SPIRIT 2013 statement: defining standard protocol items for clinical trials. *Ann Intern Med* 2013;158(3):200-7. doi: 10.7326/0003-4819-158-3-201302050-00583 [published Online First: 2013/01/09]

47. Sanya RE, Nkurunungi G, Hoek Spaans R, et al. The Impact of Intensive Versus Standard Anthelminthic Treatment on Allergy-related Outcomes, Helminth Infection Intensity, and Helminth-related Morbidity in Lake Victoria Fishing Communities, Uganda: Results From the LaVIISWA Cluster-randomized Trial. *Clinical Infectious Diseases* 2018:ciy761-ciy61. doi: 10.1093/cid/ciy761

48. Tukahebwa EM, Magnussen P, Madsen H, et al. A very high infection intensity of Schistosoma mansoni in a Ugandan Lake Victoria Fishing Community is required for association with highly prevalent organ related morbidity. *PLoS neglected tropical diseases* 2013;7(7):e2268. doi: 10.1371/journal.pntd.0002268 [published Online First: 2013/08/13]

49. Kakuru A, Jagannathan P, Muhindo MK, et al. Dihydroartemisinin–Piperaquine for the Prevention of Malaria in Pregnancy. *New England Journal of Medicine* 2016;374(10):928-39. doi: 10.1056/NEJMoa1509150

50. Nanteza B, Galukande M, Aceng J, et al. The burden of tetanus in Uganda. *SpringerPlus* 2016;5(1):705. doi: 10.1186/s40064-016-2309-z [published Online First: 2016/06/29]

51. Staedke SG, Maiteki-Sebuguzi C, Rehman AM, et al. Assessment of community-level effects of intermittent preventive treatment for malaria in schoolchildren in Jinja, Uganda (START-IPT trial): a cluster-randomised trial. *The Lancet Global Health* 2018;6(6):e668-e79. doi: https://doi.org/10.1016/S2214-109X(18)30126-8

52. Nkurunungi G, Lubyayi L, Versteeg SA, et al. Do helminth infections underpin urban-rural differences in risk factors for allergy-related outcomes? *Clinical and experimental allergy : journal of the British Society for Allergy and Clinical Immunology* 2019 doi: 10.1111/cea.13335 [published Online First: 2019/01/12]

53. Namara B, Nash S, Lule SA, et al. Effects of treating helminths during pregnancy and early childhood on risk of allergy-related outcomes: Follow-up of a randomized controlled trial. *Pediatric allergy and immunology : official publication of the European Society of Pediatric Allergy and Immunology* 2017;28(8):784-92. doi: 10.1111/pai.12804 [published Online First: 2017/09/12]

54. Webb EL, Mawa PA, Ndibazza J, et al. Effect of single-dose anthelmintic treatment during pregnancy on an infant's response to immunisation and on susceptibility to infectious diseases in infancy: a randomised, double-blind, placebo-controlled trial. *Lancet* 2011;377(9759):52-62. doi: 10.1016/S0140-6736(10)61457-2

For peer review only

For peer review only - http://bmjopen.bmj.com/site/about/guidelines.xhtml

55. Akondy RS, Johnson PL, Nakaya HI, et al. Initial viral load determines the magnitude of the human CD8 T cell response to yellow fever vaccination. *Proceedings of the National Academy of Sciences of the United States of America* 2015;112(10):3050-5. doi: 10.1073/pnas.1500475112 [published Online First: 2015/02/26]

56. Elliott AM, Mawa PA, Webb EL, et al. Effects of maternal and infant co-infections, and of maternal immunisation, on the infant response to BCG and tetanus immunisation. *Vaccine* 2010;29(2):247-55. doi: 10.1016/j.vaccine.2010.10.047

57. Naylor C, Lu M, Haque R, et al. Environmental Enteropathy, Oral Vaccine Failure and Growth Faltering in Infants in Bangladesh. *EBioMedicine* 2015;2(11):1759-66. doi: 10.1016/j.ebiom.2015.09.036 [published Online First: 2016/02/13]

58. Riner DK, Ndombi EM, Carter JM, et al. Schistosoma mansoni Infection Can Jeopardize the Duration of Protective Levels of Antibody Responses to Immunizations against Hepatitis B and Tetanus Toxoid. *PLoS neglected tropical diseases* 2016;10(12):e0005180. doi: 10.1371/journal.pntd.0005180

59. Wajja A, Kizito D, Nassanga B, et al. The effect of current Schistosoma mansoni infection on the immunogenicity of a candidate TB vaccine, MVA85A, in BCG-vaccinated adolescents: an open-label trial. *PLoS neglected tropical diseases* (in press)

60. Fletcher HA, Snowden MA, Landry B, et al. T-cell activation is an immune correlate of risk in BCG vaccinated infants. *Nat Commun* 2016;7:11290. doi: 10.1038/ncomms11290

61. Plotkin SA. Correlates of protection induced by vaccination. *Clinical and vaccine immunology : CVI* 2010;17(7):1055-65. doi: 10.1128/cvi.00131-10 [published Online First: 2010/05/14]

62. Safaeian M, Porras C, Pan Y, et al. Durable antibody responses following one dose of the bivalent human papillomavirus L1 virus-like particle vaccine in the Costa Rica Vaccine Trial. *Cancer prevention research (Philadelphia, Pa)* 2013;6(11):1242-50. doi: 10.1158/1940-6207.capr-13-0203 [published Online First: 2013/11/06]

63. Tweyongyere R, Nassanga BR, Muhwezi A, et al. Effect of Schistosoma mansoni infection and its treatment on antibody responses to measles catch-up immunisation in pre-school children: A randomised trial. *PLoS neglected tropical diseases* 2019;13(2):e0007157. doi: 10.1371/journal.pntd.0007157 [published Online First: 2019/02/15]

64. Venkatraman N, Ndiaye BP, Bowyer G, et al. Safety and Immunogenicity of a Heterologous Prime-Boost Ebola Virus Vaccine Regimen in Healthy Adults in the United Kingdom and Senegal. *The Journal of infectious diseases* 2018;219(8):1187-97. doi: 10.1093/infdis/jiy639

For Peer review only

For peer review only - http://bmjopen.bmj.com/site/about/guidelines.xhtml

65. Imai K, Keele L, Yamamoto T. Identification, inference and sensitivity analysis for causal mediation effects. *Statistical Science* 2010;25(1):51-71.

66. Prendergast AJ. Malnutrition and vaccination in developing countries. *Philosophical transactions of the Royal Society of London Series B, Biological sciences* 2015;370(1671) doi: 10.1098/rstb.2014.0141 [published Online First: 2015/05/13]

67. Harris PA, Taylor R, Minor BL, et al. The REDCap Consortium: Building an International Community of Software Platform Partners. *Journal of biomedical informatics* 2019:103208. doi: 10.1016/j.jbi.2019.103208 [published Online First: 2019/05/13]

68. Harris PA, Taylor R, Thielke R, et al. Research electronic data capture (REDCap)--a metadata-driven methodology and workflow process for providing translational research informatics support. *Journal of biomedical informatics* 2009;42(2):377-81. doi: 10.1016/j.jbi.2008.08.010 [published Online First: 2008/10/22]

69. Webb EL, Nampijja M, Kaweesa J, et al. Helminths are positively associated with atopy and wheeze in Ugandan fishing communities: results from a cross-sectional survey. *Allergy* 2016 doi: 10.1111/all.12867

FIGURE LEGENDS

Figure 1. Hypothesised pathways to population differences in vaccine responses

Figure 2. Study sites

Figure 3. Overall programme design and sample sizes

Figure 4. Outline of immunisations and interventions

[1]Primary endpoints will be at eight weeks post BCG and four weeks post Yellow fever (YF-17D), oral typhoid (Ty21a), Human Papilloma Virus (HPV) and Tetanus/diptheria (Td) vaccination.

[2]Primary endpoint for responses to Td given at 28 weeks.

Figure 5. Synthesised analysis of study objectives

Red arrows represent our principal hypotheses.

Arrows A, B and C are considered in objectives i and iv;

Arrows D, E and F in objective v;

Arrows G, H, I and J, in objective vi; as well as the fully synthesised analysis

For peer review only

For peer review only - http://bmjopen.bmj.com/site/about/guidelines.xhtml

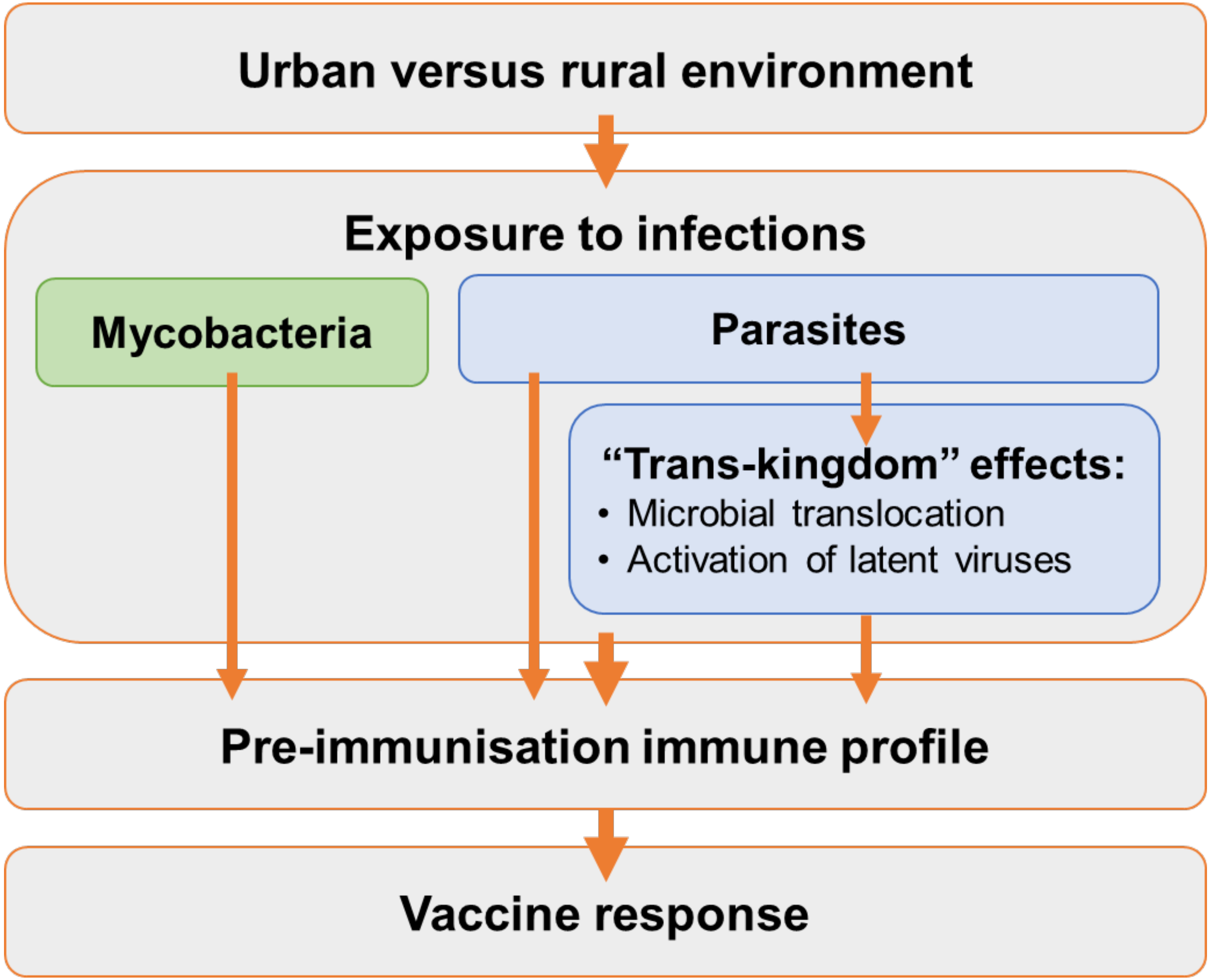
Urban versus rural environment
Exposure to infections
Mycobacteria
Parasites
“Trans-kingdom” effects:
• Microbial translocation
• Activation of latent viruses
Pre-immunisation immune profile
Vaccine response

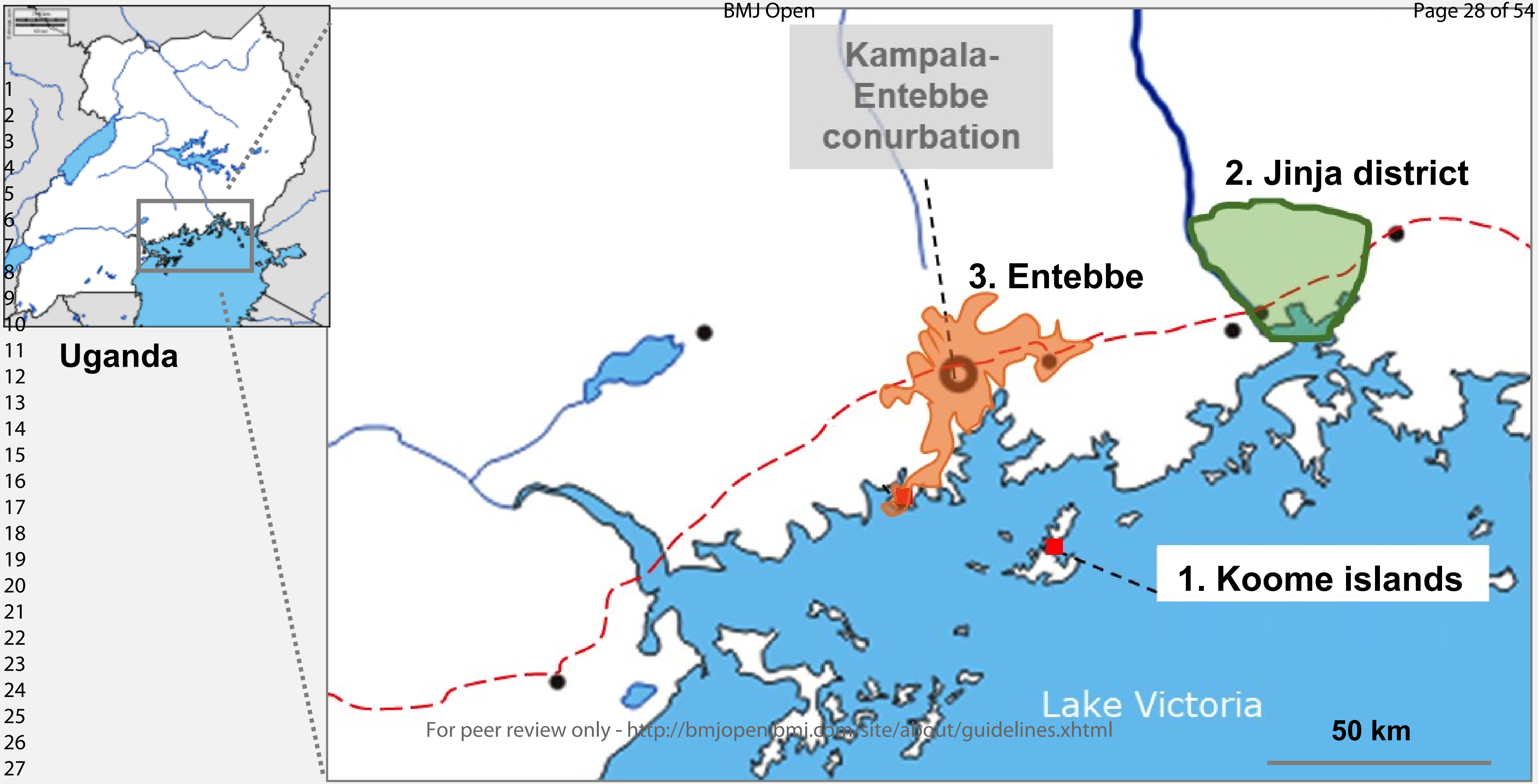

For peer review only - http://bmjopen.bmj.com/site/about/guidelines.xhtml

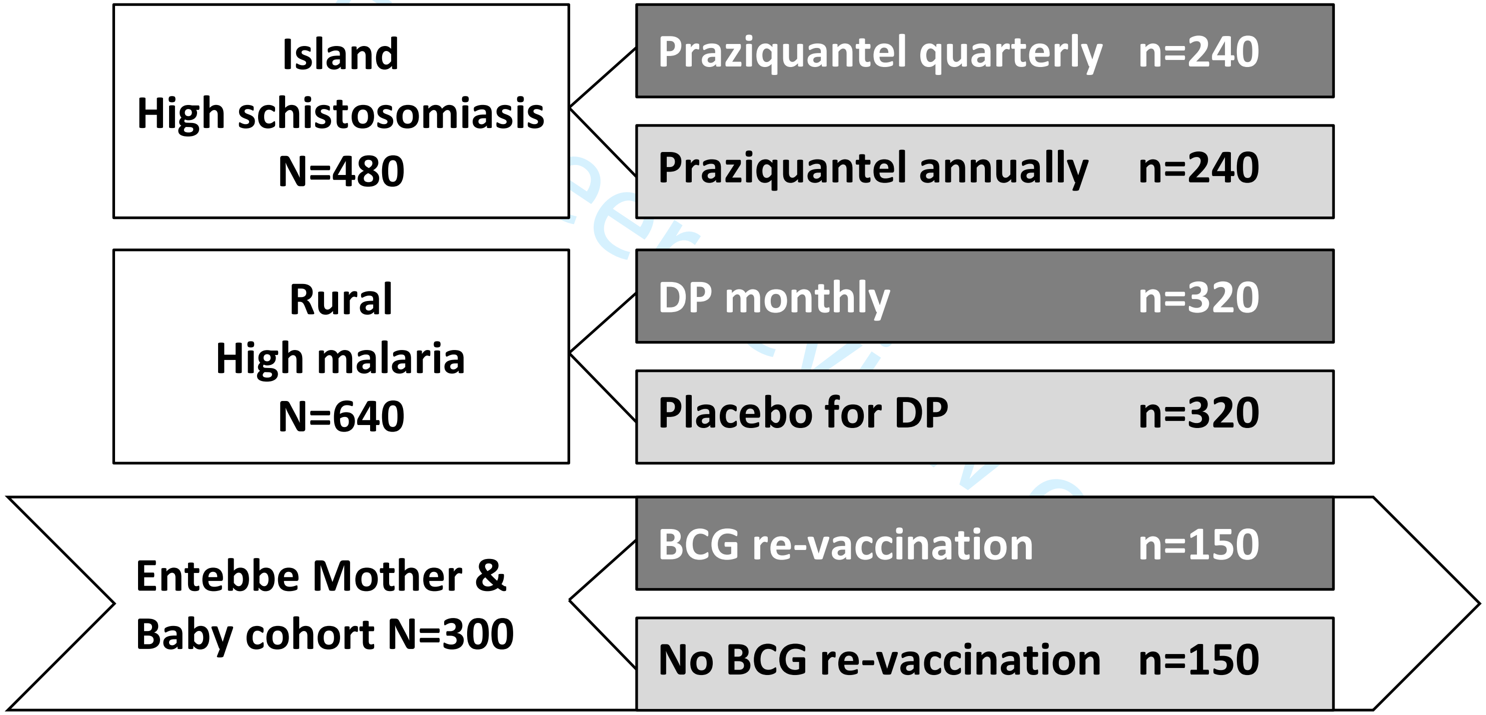
Island
High schistosomiasis
N=480
Praziquantel quarterly n=240
Praziquantel annually n=240
Rural
High malaria
N=640
DP monthly n=320
Placebo for DP n=320
Entebbe Mother & Baby cohort N=300
BCG re-vaccination n=150
No BCG re-vaccination n=150

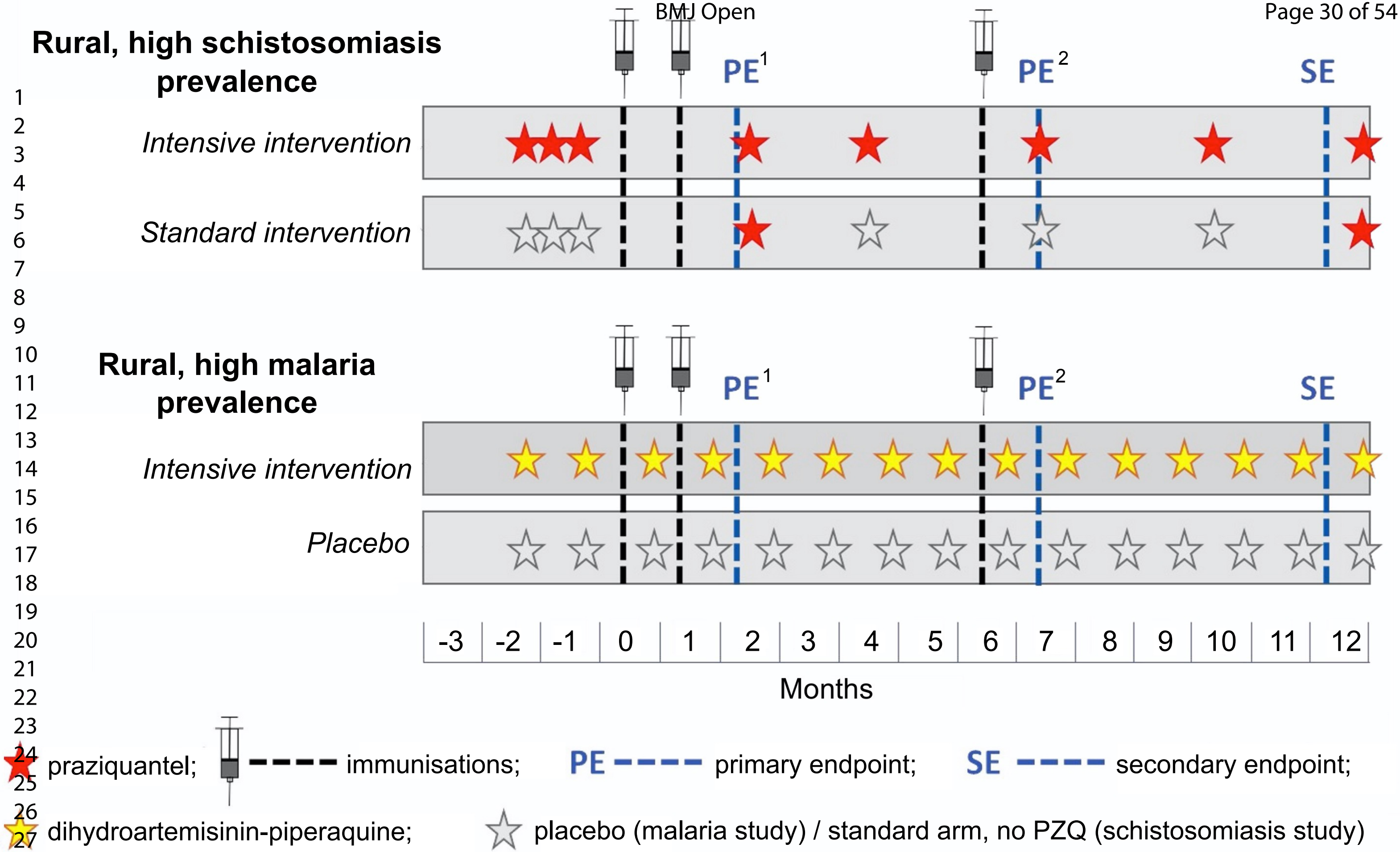
Rural, high schistosomiasis prevalence
PE1
PE2
SE
Intensive intervention
Standard intervention
Rural, high malaria prevalence
PE1
PE2
SE
Intensive intervention
Placebo
-3
-2
-1
0
1
2
3
4
5
6
7
8
9
10
11
12
Months
praziquantel;
immunisations;
PE primary endpoint;
SE secondary endpoint;
dihydroartemisinin-piperaquine;
placebo (malaria study) / standard arm, no PZQ (schistosomiasis study)

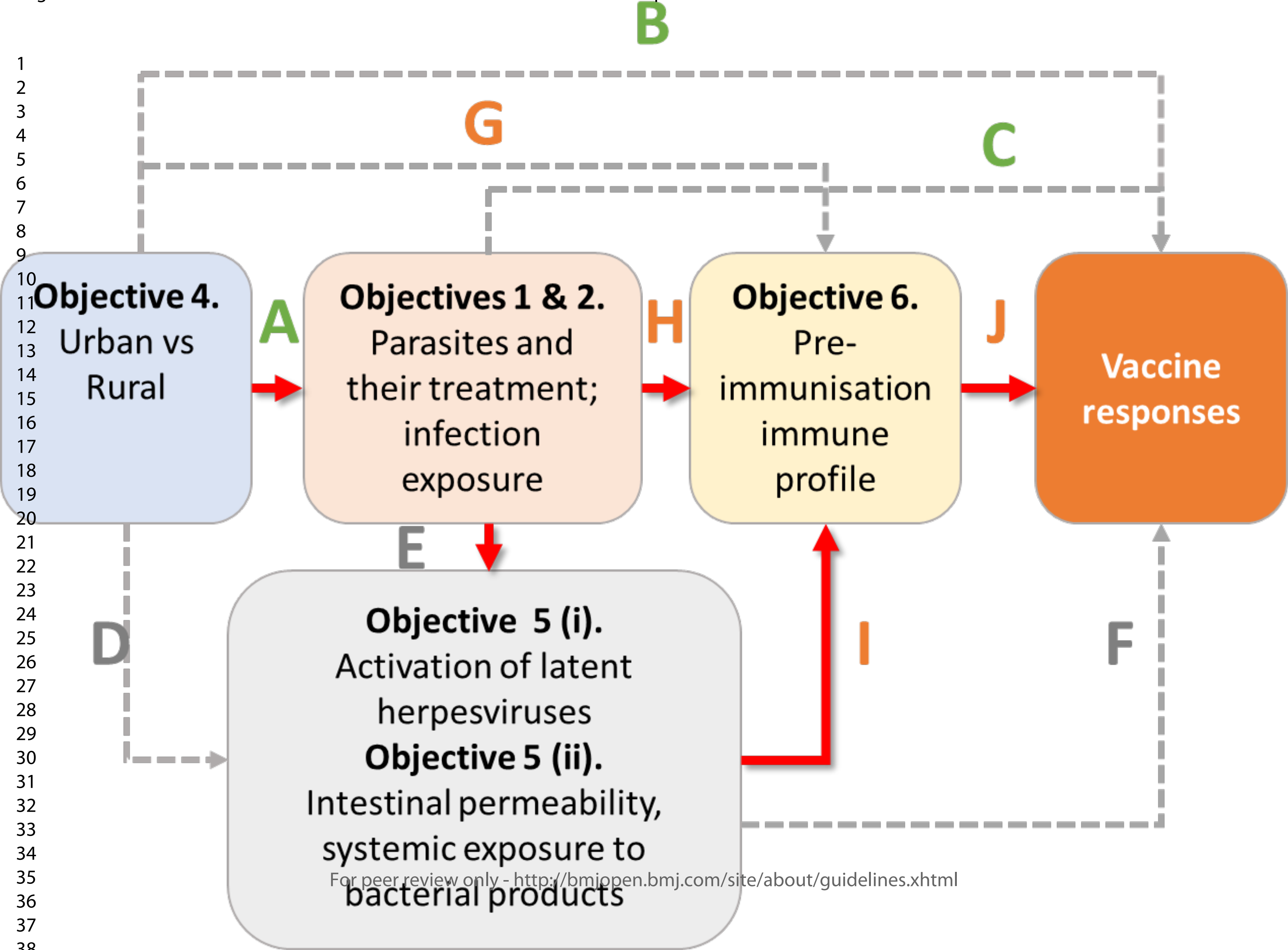
B
G
C
Objective 4.
Urban vs
Rural
A
Objectives 1 & 2.
Parasites and
their treatment;
infection
exposure
H
Objective 6.
Pre-
immunisation
immune
profile
J
Vaccine
responses
E
D
Objective 5 (i).
Activation of latent
herpesviruses
Objective 5 (ii).
Intestinal permeability,
systemic exposure to
bacterial products
I
F

SUPPLEMENTARY INFORMATION

Population differences in vaccine responses (POPVAC): scientific rationale and cross-cutting analyses for three linked, randomised controlled trials assessing the role, reversibility and mediators of immunomodulation by chronic infections in the tropics

Gyaviira Nkurunungi[1,¶,*], Ludoviko Zirimenya[1,¶], Agnes Natukunda[1,¶], Jacent Nassuuna[1], Gloria Oduru[1], Caroline Ninsiima[1], Christopher Zziwa[1], Florence Akello[1], Robert Kizindo[1], Mirriam Akello[1], Pontiano Kaleebu[1], Anne Wajja[1], Henry Luzze[2], Stephen Cose[1,3], Emily L Webb[4], Alison M Elliott[1,5] **for the POPVAC trial team**

[1]Immunomodulation and Vaccines Programme, Medical Research Council/Uganda Virus Research Institute and London School of Hygiene and Tropical Medicine (MRC/UVRI and LSHTM) Uganda Research Unit, Entebbe, Uganda

[2]Uganda National Expanded Program on Immunisation, Ministry of Health, Kampala, Uganda

[3]Department of Clinical Research, London School of Hygiene and Tropical Medicine, London, United Kingdom

[4]MRC Tropical Epidemiology Group, Department of Infectious Disease Epidemiology, London School of Hygiene and Tropical Medicine, London, United Kingdom

[¶]These authors contributed equally

***Correspondence:** Gyaviira Nkurunungi; Gyaviira.Nkurunungi@mrcuganda.org

For peer review only

Table S1. Uganda National Expanded Programme on Immunisation (EPI) schedule

Vaccine/ antigen	Dosage and doses required	Minimum Interval Between Doses	Minimum Age to Start	Mode and site of Administration	Storage Temperatures
**Infant vaccines**					
BCG	Infants (0-11m) 0.05ml. ≥11 months and 0.1ml, 1 dose	Not applicable	At birth (or first contact)	*Intradermal*, right upper arm	$+2^0C$ to $+8^0C$
DPT - HepB - Hib	0.5 ml, 3 doses	One month (4 weeks)	At 6 weeks or first contact after this age	*Intramuscular,* outer upper aspect of left thigh	$+2^0C$ to $+8^0C$ DO NOT FREEZE
PCV	0.5 mls, 3 doses	One month (4 weeks)	At 6 weeks or first contact after this age	*Intramuscular,* outer upper aspect of right thigh	$+2^0C$ to $+8^0C$ DO NOT FREEZE
Polio	2 drops, 3 doses	One month (4 weeks)	At birth or within the first 2 weeks (Polio 0) and at 6 weeks or first contact after 6 weeks (Polio 1)	*Orally*	$+2^0C$ to $+8^0C$
IPV	0.5ml, 1 dose	Nil	At 14 weeks	*Intramuscular,* left upper thigh	$+2^0C$ to $+8^0C$ DO NOT FREEZE
Rotavirus	drops, 2 doses		6 weeks or 1st contact after this age	*Orally*	
Measles	0.5 ml, 1 doses	Nil	At 9 months (or first contact after that age).	*Subcutaneous,* left upper arm	$+2^0C$ to $+8^0C$
Primary school/adolescent/adult vaccines					
Tetanus/Diphtheria	0.5 ml, 5 doses	Td1: First contact with a WCBA<br>Td2: One month after TT1<br>Td3: Six months after TT2<br>Td4: One year after TT3<br>Td5: One year after TT4	At first contact with a pregnant woman or women of childbearing age (15-49 years)	*Intramuscular,* upper arm	$+2^0C$ to $+8^0C$, DO NOT FREEZE
HPV	0.5 ml, 2 doses	HPV1: First contact with a girl in primary 4, or aged 10 years and out of school<br>HPV2: Given at 6 months after HPV1[a]	Girls in primary 4 or 10-year-old girls who are out of school	*Intramuscular,* upper arm	$+2^0C$ to $+8^0C$, DO NOT FREEZE

BCG: Bacillus Calmette Guerin. DPT: Diphtheria, Pertussis, Tetanus. Hep B: Hepatitis B. Hib: Haemophilus influenzae type B. PCV: pneumococcal conjugate vaccine. IPV: inactivated polio vaccine. HPV: Human Papilloma Virus. WCBA: woman of child-bearing age. [a]An additional dose of HPV, four weeks after the first dose, is recommended for girls aged 14 years or above receiving HPV immunisation for the first time.

For peer review only

For peer review only - http://bmjopen.bmj.com/site/about/guidelines.xhtml

Further rationale for the selection of vaccines

Bacillus Calmette–Guérin (BCG)

BCG is a live, replicating parenteral vaccine, the only licensed vaccine against TB. BCG vaccine for these studies will be obtained from the Serum Institute of India either directly, or through a supplier in Uganda. The Serum Institute of India provides much of the BCG vaccine used in Uganda.

Worldwide, TB is among the top 10 causes of death; Uganda has an estimated incidence of 202/100,000 people.[1] Infectious, sputum positive, pulmonary TB classically emerges in adolescence, driving the on-going epidemic.[2] Thus adolescent booster immunisation is a key TB control strategy.[3] However, BCG vaccine response and efficacy are often impaired in tropical and rural settings[4-6] and new TB vaccines are similarly affected.[7] In the past, WHO has been hesitant to recommend BCG re-vaccination. However, in 2017 WHO's Strategic Advisory Group of Experts (SAGE) recommended: "Further research is warranted to explore whether certain sub-groups of age, geographic or *M. tuberculosis* exposure categories would benefit from re-vaccination."[8] Recent results suggest that, despite the variability of BCG efficacy between populations, BCG vaccination in adolescence offers benefit in some tropical settings, especially for individuals who are not yet infected with *Mycobacterium tuberculosis*, and may also be cost-effective.[5 9] Also, BCG vaccine is currently being used among adolescents in South Africa as a comparator in a trial of a novel TB vaccine (trial registration NCT02075203). To our knowledge, BCG efficacy in Ugandan adolescents, and differences in BCG vaccine responses between urban and rural Ugandan populations, have not been tested. Information obtained from this study is expected to further inform the use of BCG in adolescents, and also to inform the development of new vaccines for tuberculosis.

Yellow fever vaccine

Yellow fever vaccine YF-17D is a live replicating parenteral vaccine. The vaccine (Stamaril; Sanofi Pasteur) is available for purchase in Uganda. Yellow Fever (YF) causes outbreaks in Uganda and the wider region[10] and YF-17D is a candidate for Uganda's expanded programme on immunisation (EPI). Lower vaccine replication, lower neutralising antibody induction, and greater waning, are described in Uganda compared to Switzerland.[11] YF-17D is a potential vector for novel vaccine constructs,[12] adding relevance to vaccine development.

Typhoid vaccine Ty21a

Typhoid vaccine Ty21a is a live replicating oral vaccine and also a potential vector for new vaccine constructs.[13] Ty21a vaccine will be purchased from PaxVax, Redwood City, California. Substantial, multi-year typhoid outbreaks occur in Uganda and immunisation campaigns have been advocated as

For peer review only - http://bmjopen.bmj.com/site/about/guidelines.xhtml

cost effective.[14] Schistosomiasis has been associated with prolonged *S. typhi* infection[15] and impaired antibody responses to killed typhoid vaccines.[16]

Ty21a was developed in the 1970s. Although not routinely used in Uganda, it has been (and is currently) registered in many countries. It was first registered in the United States and United Kingdom in the 1980s, and is recommended by the WHO for both endemic and epidemic settings.[17] It has comparable efficacy to the parenteral Vi polysaccharide typhoid vaccine, good durability and minimal adverse effects.[17] It is proposed for use in this study to model effects of study exposures and intervention on the response to a live oral vaccine.

The Ty21a vaccine is given as a three-dose regimen on alternate days.

Human Papilloma Virus (HPV) vaccine

Human Papilloma Virus (HPV) vaccine is a protein virus-like particle. The quadrivalent HPV Vaccine Gardasil (Merck) is available for purchase in Uganda and is the vaccine used by the national EPI programme. Studies after three vaccine doses have found somewhat enhanced responses in the presence of malaria, but no effect of helminths.[18] No study has previously investigated parasite effects on the priming response, but recent results for tetanus suggest that priming may be more susceptible than boosting to adverse effects.[19] This will be important if forthcoming trials support single-dose HPV immunisation (NCT02834637). HPV immunisation is being rolled out among girls to prevent cervical neoplasia, the commonest cancer among Ugandan women and we will coordinate provision with the national HPV immunisation programme.[20] HPV immunisation is also beneficial for boys since HPV infection is associated with anogenital warts, anal cancer and oropharyngeal cancers in both males and females, and with penile cancer in men,[21] and we will include boys in these studies.

Tetanus and diphtheria vaccines

Tetanus and diphtheria vaccines comprise inert toxoids (Td). Schistosomiasis is associated with a Th2 biased response to tetanus toxoid[22] and with suppressed antibody responses among those with low pre-immunisation antibody levels.[19] Booster immunisation is recommended for young women to prevent maternal and neonatal tetanus. Recent evidence emphasises the need to protect young men also.[23] Uganda's EPI programme recommends tetanus boosters in adolescence and plans to change from tetanus alone to Td in 2018.

For peer review only

Additional considerations regarding the vaccine schedule

Live vaccines given in combination may influence the response to each other – a phenomenon described as "interference". Observations in the 1960s suggested that elevated circulating interferon(IFN)-γ after measles immunisation might interfere with the response to Vaccinia[24] and, to avoid such interference between live vaccines, it was recommended that live vaccines be given either together or three to four weeks apart.[25] However, with the introduction of new, live vaccines into use, Public Health England reviewed and revised this recommendation in 2014, limiting it to vaccines for which there was an evidence base (**Table S2**). We have adopted a four-week interval between BCG immunisation and the other proposed live vaccines (YF and Ty21a), which will be given together.[26]

Non-live vaccines can be given at the same time as live vaccines and there are no specific recommendations as to the number of non-live vaccines that can be given together. Two injections can be given into the same muscle although it is suggested that these should be at least 2.5 cm apart in case it is necessary to distinguish local adverse reactions to the two injections.[27] Our schedule avoids giving more than two injections on the same date but at week 4 it may be appropriate to give two into the deltoid muscle of the same arm if a BCG scar is developing on the other arm. Nevertheless, uncertainties remain regarding the effects of vaccines on responses to each other. Generalisations from this programme of work will need to take potential "interference" between vaccines into account.

Table S2: Public Health England recommendations for giving more than one live attenuated vaccine in current use in the UK[26]

Vaccine combinations	Recommendations
Yellow Fever and Measles, Mumps, Rubella (MMR)	A four-week minimum interval period should be observed between the administration of these two vaccines. Yellow Fever and MMR should not be administered on the same day.
Varicella (and zoster) vaccine and MMR	If these vaccines are not administered on the same day, then a four-week minimum interval should be observed between vaccines.
Tuberculin skin testing (Mantoux) and MMR	If a tuberculin skin test has already been initiated, then MMR should be delayed until the skin test has been read unless protection against measles is required urgently. If a child has had a recent MMR, and requires a tuberculin test, then a four-week interval should be observed.
All currently used live vaccines (BCG, rotavirus, live attenuated influenza vaccine (LAIV), oral typhoid vaccine, yellow fever, varicella, zoster and MMR) and tuberculin (Mantoux) skin testing.	Apart from those combinations listed above, these live vaccines can be administered at any time before or after each other. This includes tuberculin (Mantoux) skin testing.

For peer review only - http://bmjopen.bmj.com/site/about/guidelines.xhtml

Vaccine storage and transport

In order to maintain a reliable vaccine cold chain, the vaccines and diluents to be used will be stored and transported within the recommended temperature range of +2°C to +8°C. Care will be taken to ensure that the vaccines are not frozen. BCG, being sensitive to light, will be kept in the dark (normally within its secondary packaging) for as long as possible to protect it during storage and transportation. All vaccines will be kept in appropriate refrigeration equipment with a temperature monitoring device to ensure temperatures remain between +2°C and +8°C. Cold boxes/vaccines carriers with temperature monitors will be used to transport vaccines and the diluents from the MRC/UVRI and LSHTM Uganda Research Unit (Entebbe) to Koome island and while transporting vaccines to immunization sessions. Designated staff will be given responsibility for managing the vaccine cold chain. All cold chain equipment including the temperature monitoring devices used for this project will comply with relevant technical specifications as defined by the EPI standards. Basic routine maintenance will be regularly carried out on all cold chain equipment.

Additional laboratory measurements

Additional assays will comprise HIV serology, pregnancy testing and full blood counts. HIV testing and pregnancy testing will be accompanied by appropriate counselling by trained staff.

- HIV serology will be done on blood samples using rapid tests and according to prevailing national algorithms.[28] This will be done at baseline.
- Pregnancy testing will be done using urine samples and standard operating procedures for assessment of urine β-human chorionic gonadotropin (βhCG). This will be done at baseline and before immunisation on each immunisation day.
- Full blood counts will be conducted using a haematology analyser. Mild, moderate and severe anaemia will be defined according to WHO guidelines, by age.[29] This will be done at baseline to test for anaemia as part of the eligibility assessment, and pre-immunisation as part of the assessment of immunological profile.
- Information on *S. mansoni* and malaria diagnosis before treatment and throughout the trials is detailed in the focused papers for these trials (bmjopen-2020-040426, bmjopen-2020-040427 and bmjopen-2020-040430). Briefly, current *S. mansoni* infection status and intensity will be determined by serum/plasma levels of circulating anodic antigen (CAA). In Trial A, CAA will be assessed retrospectively on stored samples collected at baseline, on immunisation days, and on primary and secondary endpoint days. In Trial B, CAA will be assessed retrospectively on stored samples collected at baseline and at weeks 28 and 52. In

For peer review only

For peer review only - http://bmjopen.bmj.com/site/about/guidelines.xhtml

Trial C, CAA will be assessed retrospectively on stored samples collected at baseline. In all three trials, current malaria infection status and intensity will be assessed retrospectively by PCR on stored blood samples collected on immunisation days and at week 52.

Individuals found to be HIV positive or pregnant will be referred to appropriate providers for further care. Individuals with severe anaemia (haemoglobin <82g/L) will be excluded from the randomised intervention (since the intervention might be beneficial in management of anaemia). They will be treated for anaemia and for any underlying cause identified.

Sample handling and archive

Blood and other samples will be processed according to local laboratory standard operating procedures (SOPs). All samples will reach the laboratory in anonymised form.

A sample archive will be developed. Although our current programme of work plans to address specific hypotheses regarding pathways of effects of parasites and interventions, the sample archive will provide a major asset for exploration of new leads arising from this work, or for an alternative, "systems biology" approach employing (for example) proteomic, genomic, epigenetic and transcriptomic analyses, and investigating the microbiome and virome. Information provided to participants, and consent forms, will include considerations of sample storage, and the possibility of sample analysis in laboratories within and outside Uganda. Participants will be able to decide if they will permit such future use of any leftover samples. We plan to store the samples for up to 20 years. If further storage is needed after that time, permission will be requested from the Uganda Virus Research Institute and London School of Hygiene and Tropical Medicine review committees. If they elect not to permit this, all of those leftover samples will be discarded after the completion of the work included in the current protocol.

For peer review only

Operational considerations

Programme governance

A Programme Steering Committee has been set up to guide progress across all projects. This comprises the following:

- An independent chair
- Representatives from the Ministry of Health programmes for immunisation and for vector borne disease control
- Representatives of district authorities (Mukono and Jinja districts)
- Community representatives
- Principal investigator and co-investigators

- Project leader and post-doctoral immunologist
- Trial statistician
- Laboratory manager
- Medical Research Council observer

Informed consent

Both written informed assent from the participants and written informed consent from a parent or guardian will be required for participation, although these may not necessarily be obtained at the same time. Information will be provided in both English and the appropriate local language. For individuals who cannot speak the languages used, or who cannot read or write, a witness who can read the information sheet and translate the information to the participant or parent/guardian will be used. For trials A and B, two different types of age specific assent forms will be used for the group of participants aged 9 – 12 years and for the group aged 13 – 17 years. Informed consent by emancipated or mature minors will be obtained using a designated consent form for these categories of participants

The aims of the study, all tests, treatments and immunisations to be carried out and potential risks will be explained. The participant will be given the opportunity to ask about details of the trial, and will then have time to consider whether or not to participate. If they do decide to participate, they and their parent/guardian will sign and date two copies of the assent and consent forms, one for them to take away and keep, and one to be stored securely by the research team. Separate information and consent forms will be provided (i) for consent for storage of samples for future studies and for anonymous sharing of data from this study and (ii) for possible genetic studies (for the urban cohort genetic data are already available based on previous approval); the information sheet will explain that these data may be used in analyses related to this protocol.

Screening and Eligibility Assessment

Once the informed consent process has been completed, and consent (and assent) given, a baseline medical history (including concomitant medication) will be collected. Vital signs will be checked and a physical examination will be performed. Inclusion and exclusion criteria will be checked.

Participants will undergo pre- and post-test counselling for HIV and (for girls) pregnancy testing by a trained and experienced nurse- or clinician-counsellor. Blood, urine and stool samples will be obtained, for tests as specified in the schedule of procedures. These tests are to exclude the major, immunomodulating co-infection, HIV, and conditions that might impact safety (anaemia, pregnancy).

For peer review only

Enrolment

Participants who consent/assent, complete the screening processes, satisfy all the inclusion criteria and meet none of the exclusion criteria will be enrolled.

Discontinuation / withdrawal criteria

In accordance with the principles of the current revision of the Declaration of Helsinki and any other applicable regulations, a participant has the right to withdraw from the study at any time and for any reason, and is not obliged to give his or her reasons for doing so. The Investigator may withdraw the participant at any time in the interests of the participant's health and well-being. In addition, the participant may withdraw/be withdrawn for any of the following reasons:

- Ineligibility (either arising during the study or retrospectively, having been overlooked at screening)
- Administrative decision by the Investigator
- Significant protocol deviation
- Participant non-compliance with study requirements
- An adverse event which requires discontinuation of the study involvement or results in inability to continue to comply with study procedures.

Any participant who becomes pregnant during the trial will be followed up until the end of the pregnancy but no further immunisations will be given unless indicated during pregnancy (as is the case for tetanus toxoid). The trial allocation for this participant will be unblinded and the participant will only be given further treatment if clinically indicated. The babies will also be followed up and examined for any adverse effects. We will not routinely perform venipuncture in a pregnant participant.

The reason for withdrawal will be recorded in the case report form (CRF). If withdrawal is due to an AE, appropriate follow-up visits or medical care will be arranged, with the agreement of the participant, until the AE has resolved, stabilised or a non-trial related causality has been assigned.

If a participant withdraws from the study samples collected before their withdrawal from the trial will be used/ stored unless the participant specifically requests otherwise.

Trial discontinuation

The trial will be discontinued in the event of new scientific information that renders continuation futile or unethical, or for any other reason, at the discretion of the Programme Steering Committee.

For peer review only

End of study definition

The trial will be completed when the last participant enrolled into the trial has completed their final follow up visit.

Safety assessments and oversight

No new investigational drug or product will be used in the proposed trial. However, standard approaches for monitoring safety and reporting of serious adverse events will be followed.

Monitoring

The trial will be monitored by both internal and external monitors according to a pre-defined monitoring plan which will include a site initiation visit, monitoring visits at least annually, and a close-out visit. The monitors will assess patient safety, data integrity, and adherence to the protocol and to Good Clinical Research Practice procedures.

Considerations regarding standard of care for parasitic infections

Malaria and *S. mansoni* infection status will be determined retrospectively through assays conducted in bulk on stored samples. These results will not, therefore, be useful to determine management of individual participants.

Participants in the standard anthelminthic treatment (trial A) and the malaria placebo (trial B) arms will receive lower levels of treatment. However, all trial arms will receive a minimum of well-implemented national standard of care.

In trial A, standard of care will comprise annual praziquantel treatment. Our own results from the Lake Victoria Island Intervention Study on Worms and Allergy-related diseases (LaVIISWA),[30] which compared annual versus quarterly intervention for schistosomiasis at community level over three years, showed no advantage of quarterly treatment for morbidity outcomes attributed to schistosomiasis. Schistosomiasis can cause anaemia. To manage the expected differential benefits of the interventions for anaemia, a full blood count will be performed at baseline, as discussed above; anaemic children will be managed appropriately and severely anaemic children excluded. Albendazole will be provided twice a year to manage nematode infections (after collection of primary and secondary endpoint samples).

Dihydroartemisinin/piperaquine is considered an attractive option for preventive treatment and preventive chemotherapy for malaria because of the long half-life of piperaquine (approximately 23 days).[31] Monthly treatment with DP has been shown to reduce the prevalence of anaemia and reduce episodes of clinical malaria in Ugandan schools[32] but has not been adopted as standard of

For peer review only - http://bmjopen.bmj.com/site/about/guidelines.xhtml

care. Trial B is expected to add further evidence regarding the potential benefits of monthly DP for school children by determining the effect on vaccine responses, thereby further contributing to policy debate in this field. To manage the expected differential benefits of the interventions for anaemia, a full blood count will be performed at baseline, as discussed above; anaemic children will be managed appropriately and severely anaemic children excluded. Malaria standard of care will comprise provision of bed nets to minimise malaria exposure for all participants. Rapid diagnostic tests and treatment will be made readily available for participants who develop symptomatic malaria. Albendazole will be provided twice a year to manage nematode infections (after collection of primary and secondary endpoint samples).

Procedures to be followed in the event of abnormal findings

Abnormal clinical findings from medical history, examination or blood tests will be assessed as to their clinical significance throughout the trials. If an abnormal test result is deemed clinically significant, it may be repeated. If a test remains clinically significant, the participant will be informed and appropriate medical care arranged as appropriate and with the permission of the participant. Specific details regarding findings, discussion with participants and resulting actions will be recorded in the clinical records. Decisions to exclude the participant from enrolling in the trial or to withdraw a participant from the trial will be at the discretion of the Investigator.

Data and Safety Monitoring Board (DSMB)

The DSMB will be notified within 7 days of the Investigators' being aware of the occurrence of SAEs. The DSMB may recommend the Investigators to place the trial on hold if deemed necessary following an intervention-related SAE. The DSMB will be chaired by a clinician experienced in clinical trials. There will be a minimum of two other appropriately qualified committee members. In the case of events related to a blinded intervention, the DSMB can request unblinding. Membership will include a statistician, and at least one Ugandan member. All correspondence between Investigators and the DSMB will be conveyed by the Principal Investigator to the trial Sponsor. The Chair of the DSMB will be contacted for advice and independent review by the Investigator or trial Sponsor in the following situations:

- The occurrence of any SAE
- Any other situation where the Investigator or trial Sponsor feels independent advice or review is important

Ethical and regulatory considerations

Ethical approvals

For peer review only - http://bmjopen.bmj.com/site/about/guidelines.xhtml

294 *Trial A*

295 Ethical approval has been granted from the Research Ethics Committee of the Uganda Virus Research Institute (UVRI REC, reference: GC/127/19/05/664) and the London School of Hygiene and Tropical Medicine (LSHTM, reference: 16032), and from the Uganda National Council for Science and Technology (UNCST, reference: HS2486) and the Uganda National Drug Authority (NDA, reference: CTA0093).

300 *Trial B*

301 Ethical approval has been granted from the Research Ethics Committees of the Uganda Virus Research Institute (UVRI REC, reference: GC/127/19/05/681) and the London School of Hygiene and Tropical Medicine (LSHTM, reference: 16033), and from the Uganda National Council for Science and Technology (UNCST, reference: HS 2487) and the Uganda National Drug Authority (NDA, reference: CTC0117/2020).

306 *Trial C*

307 Ethical approval has been granted from the Research Ethics Committees of the Uganda Virus Research Institute (reference: GC/127/19/05/682), the London School of Hygiene and Tropical Medicine (reference: 16034), the Uganda National Council for Science and Technology (reference: HS 2491) and from the Uganda National Drug Authority (certificate number: CTA0094).

311 *Further information regarding risks*

312 The immunisations to be given have recognised side effects which are usually mild and resolve spontaneously in a few days to one week. Parenteral vaccines are likely to result in pain and swelling at the site of injection and mild fever; very occasionally pain and swelling can be severe and associated with difficulty in moving the shoulder. Sometimes headache and tiredness occur. Rarely a vaccine may cause a severe allergic reaction. For most vaccines this is estimated at less than one in a million doses (but 1 in 55,000 for Yellow Fever vaccine).[33] Individuals with a history of a possible allergic reaction to drugs or vaccines, or to vaccine components including eggs or chicken proteins, will be excluded from the studies. The research team will be trained and prepared to manage severe allergic reactions.

321 Adverse reactions to Yellow Fever vaccine include severe nervous system reaction (about 1 person in 125,000) and severe, life-threatening illness with organ failure (about 1 person in 250,000). The mortality for this severe, life-threatening adverse effect is reported as about 50%.[33]

324 BCG immunisation is likely to induce a scar in many cases. This may develop over several weeks, starting as a small papule at the injection site which may become ulcerated and then heal over a

For peer review only - http://bmjopen.bmj.com/site/about/guidelines.xhtml

period of 2 to 5 months; and lymphadenopathy may develop. Occasionally a more severe local reaction occurs (estimated at 1 per 1,000-10,000 doses): for example, an abscess develops and scars may develop into keloids. Rarely BCG can cause disseminated disease (1 per 230,000 to 640,000 doses), or disease in sites remote from the immunisation site. Disseminated BCG disease usually occurs in immunocompromised people: HIV positive people will be excluded from these studies.[34] BCG "pre-immunisation" may interfere with the response to the subsequent live vaccines; indeed our hypothesis, and published results, suggest that it may suppress replication of YF 17D vaccine.[35] However, this reduced replication has not been shown to correlate with, or result in, reduced levels of neutralising antibody titres (which are the desired protective outcome).[11 35]

Oral typhoid vaccine (Ty21a) may occasionally be associated with stomach pain, nausea, vomiting and (rarely) rash.[33]

Praziquantel has been in use for about 30 years. It has a well-recognised profile of side effects including dizziness, nausea, vomiting, abdominal pain, diarrhoea (sometimes with blood) and urticarial rash. The symptoms are considered to arise largely from the effects of killing worms and to be more severe in people with heavy infections. Symptoms are better tolerated when the drugs are given after food and we will provide treatment after a meal or snack. Simple medications, such as paracetamol and cetirizine, can alleviate symptoms and these will be available on treatment days.

More information on causal mediation analysis

Causal mediation analysis is a statistical approach that aims to assess the relative importance of intermediate variables (mediators) through which an exposure may affect an outcome. It uses a counterfactual framework. In essence, this investigates how, for each individual, values of the mediator and outcome might change if their main exposure status changed. For example, in Objective iv, it will be used in exploratory analysis to assess whether, and to what degree, any differences in vaccine response (the outcome) by setting (the exposure) may be explained by differences in current or previous *S. mansoni* and *P. falciparum* infection experience (the potential mediators). An alternative approach would be to use a path analytic approach such as structural equation modelling, but estimation of pathway-specific effects using this technique requires strong assumptions regarding linearity and normality and lack of pairwise confounding for all variables in the model. Causal mediation analysis is itself subject to key assumptions regarding confounding between exposure, mediator and outcomes, and we will assess the potential impact of violations in these assumptions using sensitivity analysis. Specifically, we will assess the degree of uncontrolled confounding that would substantially change conclusions regarding pathway-specific effects using techniques such as those described by Hafemen (2011).[36] Regarding the mediators for Objective iv,

For peer review only - http://bmjopen.bmj.com/site/about/guidelines.xhtml

359 we will consider *S. mansoni* and *P. falciparum* separately, under the assumption that there is no
360 direct causal link between these infections. For each infection, we will include both prior exposure
361 and current infection as mediators in the same set of models (**Figure S1**), allowing the possibility that
362 current infection depends on prior exposure, using the approach outlined in Steen *et al*.[37]

363

Figure S1. Causal diagram for assessment of whether exposure to parasites mediates differences in vaccine response between urban and rural settings

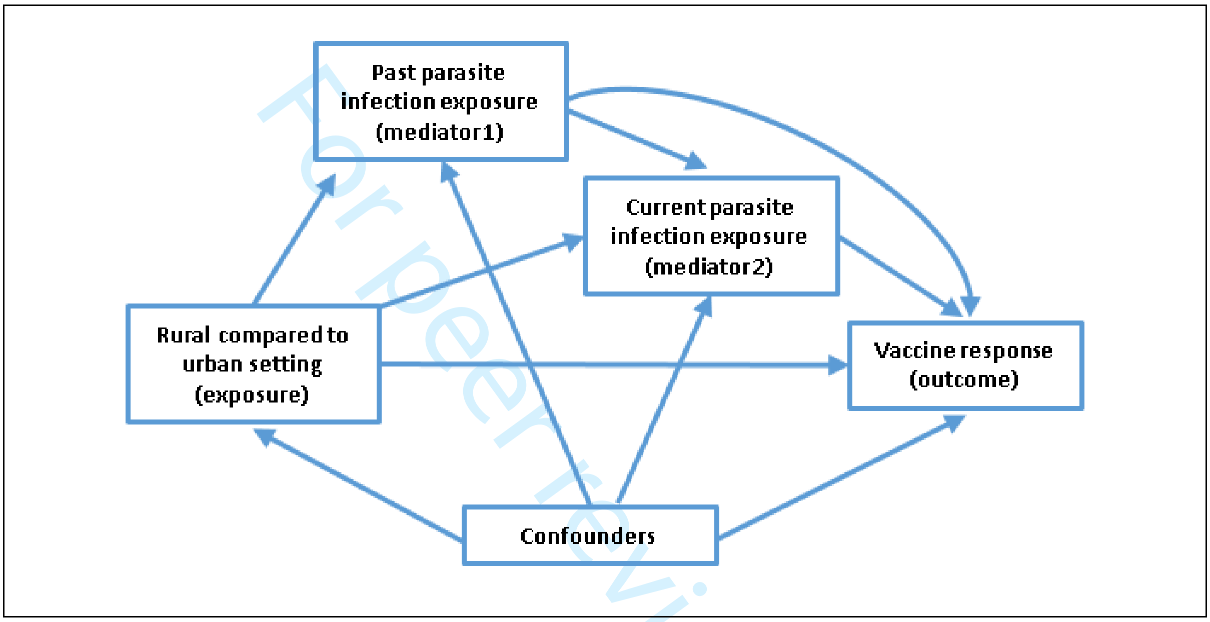

364

For peer review only

References

1. WHO. Global tuberculosis report 2016 http://www.who.int/tb/publications/global_report/en/ (accessed 17 June 2017), 2016.

2. Alcais A, Fieschi C, Abel L, et al. Tuberculosis in children and adults: two distinct genetic diseases. *J Exp Med* 2005;202(12):1617-21. doi: 10.1084/jem.20052302

3. Weiner J, 3rd, Kaufmann SH. Recent advances towards tuberculosis control: vaccines and biomarkers. *J Intern Med* 2014;275(5):467-80. doi: 10.1111/joim.12212

4. Fine PE. Variation in protection by BCG: implications of and for heterologous immunity. *Lancet* 1995;346(8986):1339-45.

5. Barreto ML, Pilger D, Pereira SM, et al. Causes of variation in BCG vaccine efficacy: examining evidence from the BCG REVAC cluster randomized trial to explore the masking and the blocking hypotheses. *Vaccine* 2014;32(30):3759-64. doi: 10.1016/j.vaccine.2014.05.042

6. Black GF, Weir RE, Floyd S, et al. BCG-induced increase in interferon-gamma response to mycobacterial antigens and efficacy of BCG vaccination in Malawi and the UK: two randomised controlled studies. *Lancet* 2002;359(9315):1393-401. doi: 10.1016/S0140-6736(02)08353-8

7. Tanner R, Kakalacheva K, Miller E, et al. Serum indoleamine 2,3-dioxygenase activity is associated with reduced immunogenicity following vaccination with MVA85A. *BMC infectious diseases* 2014;14:660. doi: 10.1186/s12879-014-0660-7 [published Online First: 2014/12/04]

8. WHO. SAGE Evidence to recommendations framework. 2017. http://www.who.int/immunization/sage/meetings/2017/october/2_EvidencetoRecommendationFramework_BCG.pdf (accessed 16th March 2018).

9. Dye C. Making wider use of the world's most widely used vaccine: Bacille Calmette-Guerin revaccination reconsidered. *Journal of the Royal Society, Interface* 2013;10(87):20130365. doi: 10.1098/rsif.2013.0365 [published Online First: 2013/08/02]

10. Kraemer MU, Faria NR, Reiner RC, Jr., et al. Spread of yellow fever virus outbreak in Angola and the Democratic Republic of the Congo 2015-16: a modelling study. *The Lancet Infectious diseases* 2016 doi: 10.1016/s1473-3099(16)30513-8 [published Online First: 2016/12/27]

11. Muyanja E, Ssemaganda A, Ngauv P, et al. Immune activation alters cellular and humoral responses to yellow fever 17D vaccine. *The Journal of clinical investigation* 2014;124(7):3147-58. doi: 10.1172/jci75429 [published Online First: 2014/06/10]

12. Aguiar M, Stollenwerk N, Halstead SB. The Impact of the Newly Licensed Dengue Vaccine in Endemic Countries. *PLoS neglected tropical diseases* 2016;10(12):e0005179. doi: 10.1371/journal.pntd.0005179 [published Online First: 2016/12/22]

13. Dharmasena MN, Osorio M, Filipova S, et al. Stable expression of Shigella dysenteriae serotype 1 O-antigen genes integrated into the chromosome of live Salmonella oral vaccine vector Ty21a. *Pathogens and disease* 2016 doi: 10.1093/femspd/ftw098 [published Online First: 2016/09/23]

14. Carias C, Walters MS, Wefula E, et al. Economic evaluation of typhoid vaccination in a prolonged typhoid outbreak setting: the case of Kasese district in Uganda. *Vaccine* 2015;33(17):2079-85. doi: 10.1016/j.vaccine.2015.02.027 [published Online First: 2015/02/26]

15. Melhem RF, LoVerde PT. Mechanism of interaction of Salmonella and Schistosoma species. *Infection and immunity* 1984;44(2):274-81. [published Online First: 1984/05/01]

For peer review only

For peer review only - http://bmjopen.bmj.com/site/about/guidelines.xhtml

16. Muniz-Junqueira MI, Tavares-Neto J, Prata A, et al. Antibody response to Salmonella typhi in human schistosomiasis mansoni. *Revista da Sociedade Brasileira de Medicina Tropical* 1996;29(5):441-5. [published Online First: 1996/09/01]

17. WHO. Position Paper on Typhoid vaccines: WHO position paper – March 2018 2018

18. Brown J, Baisley K, Kavishe B, et al. Impact of malaria and helminth infections on immunogenicity of the human papillomavirus-16/18 AS04-adjuvanted vaccine in Tanzania. *Vaccine* 2014;32(5):611-7. doi: 10.1016/j.vaccine.2013.11.061

19. Riner DK, Ndombi EM, Carter JM, et al. Schistosoma mansoni Infection Can Jeopardize the Duration of Protective Levels of Antibody Responses to Immunizations against Hepatitis B and Tetanus Toxoid. *PLoS neglected tropical diseases* 2016;10(12):e0005180. doi: 10.1371/journal.pntd.0005180

20. Centre HI. HPV and related diseases report: Uganda. 2016. http://www.hpvcentre.net/statistics/reports/UGA.pdf (accessed 20.01.2017).

21. WHO. Human papillomavirus vaccines: WHO position paper, May 2017. *Releve epidemiologique hebdomadaire* 2017;92(19):241-68. [published Online First: 2017/05/23]

22. Sabin EA, Araujo MI, Carvalho EM, et al. Impairment of tetanus toxoid-specific Th1-like immune responses in humans infected with Schistosoma mansoni. *The Journal of infectious diseases* 1996;173(1):269-72. [published Online First: 1996/01/01]

23. Nanteza B, Galukande M, Aceng J, et al. The burden of tetanus in Uganda. *SpringerPlus* 2016;5(1):705. doi: 10.1186/s40064-016-2309-z [published Online First: 2016/06/29]

24. Petralli JK, Merigan TC, Wilbur JR. ACTION OF ENDOGENOUS INTERFERON AGAINST VACCINIA INFECTION IN CHILDREN. *Lancet* 1965;2(7409):401-5. [published Online First: 1965/08/28]

25. C-A S. Vaccine Immunology. In: Plotkin SA, Orenstein W, Offit P, et al., eds. Vaccines: Elsevier 2017.

26. PHE. Public Health England: revised recommendations for the administration of more than one live vaccine 2015

27. PHE. Immunization procedures. The Green Book. Public Health England. 2012

28. Ministry of Health–Uganda. Uganda Clinical Guidelines 2016. http://apps.who.int/medicinedocs/documents/s23532en/s23532en.pdf

29. WHO. Haemoglobin concentrations for the diagnosis of anaemia and assessment of severity. Vitamin and Mineral Nutrition Information System. Geneva, World Health Organization, 2011 (WHO/NMH/NHD/MNM/11.1).

30. Sanya RE, Nkurunungi G, Hoek Spaans R, et al. The Impact of Intensive Versus Standard Anthelminthic Treatment on Allergy-related Outcomes, Helminth Infection Intensity, and Helminth-related Morbidity in Lake Victoria Fishing Communities, Uganda: Results From the LaVIISWA Cluster-randomized Trial. *Clinical Infectious Diseases* 2018:ciy761-ciy61. doi: 10.1093/cid/ciy761

31. Gutman J, Kovacs S, Dorsey G, et al. Safety, tolerability, and efficacy of repeated doses of dihydroartemisinin-piperaquine for prevention and treatment of malaria: a systematic review and meta-analysis. *The Lancet Infectious diseases* 2017;17(2):184-93. doi: 10.1016/s1473-3099(16)30378-4 [published Online First: 2016/11/21]

32. Nankabirwa JI, Wandera B, Amuge P, et al. Impact of intermittent preventive treatment with dihydroartemisinin-piperaquine on malaria in Ugandan schoolchildren: a randomized, placebo-controlled

For peer review only - http://bmjopen.bmj.com/site/about/guidelines.xhtml

trial. *Clinical infectious diseases : an official publication of the Infectious Diseases Society of America* 2014;58(10):1404-12. doi: 10.1093/cid/ciu150 [published Online First: 2014/03/14]

33. CDC. Centers for Disease Control and Prevention, vaccines and immunizations.

34. WHO. Information sheet observed rate of vaccine reactions Bacille Calmette-Guérin (BCG) vaccine. 2012

35. Arts RJW, Moorlag S, Novakovic B, et al. BCG Vaccination Protects against Experimental Viral Infection in Humans through the Induction of Cytokines Associated with Trained Immunity. *Cell host & microbe* 2018;23(1):89-100.e5. doi: 10.1016/j.chom.2017.12.010 [published Online First: 2018/01/13]

36. Hafeman DM. Confounding of indirect effects: a sensitivity analysis exploring the range of bias due to a cause common to both the mediator and the outcome. *American journal of epidemiology* 2011;174(6):710-7. doi: 10.1093/aje/kwr173 [published Online First: 2011/06/10]

37. Steen J, Loeys T, Moerkerke B, et al. Flexible Mediation Analysis With Multiple Mediators. *American journal of epidemiology* 2017;186(2):184-93. doi: 10.1093/aje/kwx051 [published Online First: 2017/05/05]

For peer review only

Reporting checklist for protocol of a clinical trial.

Based on the SPIRIT guidelines.

Instructions to authors

Complete this checklist by entering the page numbers from your manuscript where readers will find each of the items listed below.

Your article may not currently address all the items on the checklist. Please modify your text to include the missing information. If you are certain that an item does not apply, please write "n/a" and provide a short explanation.

Upload your completed checklist as an extra file when you submit to a journal.

In your methods section, say that you used the SPIRITreporting guidelines, and cite them as:

Chan A-W, Tetzlaff JM, Altman DG, Laupacis A, Gøtzsche PC, Krleža-Jerić K, Hróbjartsson A, Mann H, Dickersin K, Berlin J, Doré C, Parulekar W, Summerskill W, Groves T, Schulz K, Sox H, Rockhold FW, Rennie D, Moher D. SPIRIT 2013 Statement: Defining standard protocol items for clinical trials. Ann Intern Med. 2013;158(3):200-207

		Reporting Item	Page Number
**Administrative information**			
Title	#1	Descriptive title identifying the study design, population, interventions, and, if applicable, trial acronym	1
Trial registration	#2a	Trial identifier and registry name. If not yet registered, name of intended registry	2
Trial registration: data set	#2b	All items from the World Health Organization Trial Registration Data Set	n/a
Protocol version	#3	Date and version identifier	Information available at ISRCTN60517191, ISRCTN62041885, ISRCTN10482904.
Funding	#4	Sources and types of financial, material, and other support	16

For peer review only

Roles and responsibilities: contributorship	#5a	Names, affiliations, and roles of protocol contributors	16
Roles and responsibilities: sponsor contact information	#5b	Name and contact information for the trial sponsor	Information available at ISRCTN60517191, ISRCTN62041885, ISRCTN10482904.
Roles and responsibilities: sponsor and funder	#5c	Role of study sponsor and funders, if any, in study design; collection, management, analysis, and interpretation of data; writing of the report; and the decision to submit the report for publication, including whether they will have ultimate authority over any of these activities	16
Roles and responsibilities: committees	#5d	Composition, roles, and responsibilities of the coordinating centre, steering committee, endpoint adjudication committee, data management team, and other individuals or groups overseeing the trial, if applicable (see Item 21a for data monitoring committee)	Details in supplementary information
**Introduction**			
Background and rationale	#6a	Description of research question and justification for undertaking the trial, including summary of relevant studies (published and unpublished) examining benefits and harms for each intervention	4
Background and rationale: choice of comparators	#6b	Explanation for choice of comparators	7
Objectives	#7	Specific objectives or hypotheses	6
Trial design	#8	Description of trial design including type of trial (eg, parallel group, crossover, factorial, single group), allocation ratio, and framework (eg, superiority, equivalence, non-inferiority, exploratory)	6,7

For peer review only

**Methods: Participants, interventions, and outcomes**			
Study setting	#9	Description of study settings (eg, community clinic, academic hospital) and list of countries where data will be collected. Reference to where list of study sites can be obtained	6,7
Eligibility criteria	#10	Inclusion and exclusion criteria for participants. If applicable, eligibility criteria for study centres and individuals who will perform the interventions (eg, surgeons, psychotherapists)	7
Interventions: description	#11a	Interventions for each group with sufficient detail to allow replication, including how and when they will be administered	7
Interventions: modifications	#11b	Criteria for discontinuing or modifying allocated interventions for a given trial participant (eg, drug dose change in response to harms, participant request, or improving / worsening disease)	Detailed in supplementary information files
Interventions: adherance	#11c	Strategies to improve adherence to intervention protocols, and any procedures for monitoring adherence (eg, drug tablet return; laboratory tests)	Detailed in supplementary information files
Interventions: concomitant care	#11d	Relevant concomitant care and interventions that are permitted or prohibited during the trial	Detailed in supplementary information files
Outcomes	#12	Primary, secondary, and other outcomes, including the specific measurement variable (eg, systolic blood pressure), analysis metric (eg, change from baseline, final value, time to event), method of aggregation (eg, median, proportion), and time point for each outcome. Explanation of the clinical relevance of chosen efficacy and harm outcomes is strongly recommended	9

For peer review only

Participant timeline	#13	Time schedule of enrolment, interventions (including any run-ins and washouts), assessments, and visits for participants. A schematic diagram is highly recommended (see Figure)	Detailed in supplementary information files for protocols A, B and C
Sample size	#14	Estimated number of participants needed to achieve study objectives and how it was determined, including clinical and statistical assumptions supporting any sample size calculations	9
Recruitment	#15	Strategies for achieving adequate participant enrolment to reach target sample size	Detailed in supplementary information files for protocols A, B and C
**Methods: Assignment of interventions (for controlled trials)**			
Allocation: sequence generation	#16a	Method of generating the allocation sequence (eg, computer-generated random numbers), and list of any factors for stratification. To reduce predictability of a random sequence, details of any planned restriction (eg, blocking) should be provided in a separate document that is unavailable to those who enrol participants or assign interventions	Detailed in protocols A, B and C
Allocation concealment mechanism	#16b	Mechanism of implementing the allocation sequence (eg, central telephone; sequentially numbered, opaque, sealed envelopes), describing any steps to conceal the sequence until interventions are assigned	Detailed in protocols A, B and C
Allocation: implementation	#16c	Who will generate the allocation sequence, who will enrol participants, and who will assign participants to interventions	Detailed in protocols A, B and C
Blinding (masking)	#17a	Who will be blinded after assignment to interventions (eg, trial participants, care	Detailed in protocols A, B and C

For peer review only

		providers, outcome assessors, data analysts), and how	
Blinding (masking): emergency unblinding	#17b	If blinded, circumstances under which unblinding is permissible, and procedure for revealing a participant's allocated intervention during the trial	Detailed in protocols A, B and C
**Methods: Data collection, management, and analysis**			
Data collection plan	#18a	Plans for assessment and collection of outcome, baseline, and other trial data, including any related processes to promote data quality (eg, duplicate measurements, training of assessors) and a description of study instruments (eg, questionnaires, laboratory tests) along with their reliability and validity, if known. Reference to where data collection forms can be found, if not in the protocol	Detailed in protocols A, B and C
Data collection plan: retention	#18b	Plans to promote participant retention and complete follow-up, including list of any outcome data to be collected for participants who discontinue or deviate from intervention protocols	Detailed in protocols A, B and C
Data management	#19	Plans for data entry, coding, security, and storage, including any related processes to promote data quality (eg, double data entry; range checks for data values). Reference to where details of data management procedures can be found, if not in the protocol	Detailed in protocols A, B and C
Statistics: outcomes	#20a	Statistical methods for analysing primary and secondary outcomes. Reference to where other details of the statistical analysis plan can be found, if not in the protocol	11, 12, and in protocols A, B and C. These will also be detailed in a statistical analysis plans that will be uploaded to the online trial registrations.

For peer review only

Statistics: additional analyses	#20b	Methods for any additional analyses (eg, subgroup and adjusted analyses)	11, 12, and in protocols A, B and C. These will also be detailed in a statistical analysis plans that will be uploaded to the online trial registrations.
Statistics: analysis population and missing data	#20c	Definition of analysis population relating to protocol non-adherence (eg, as randomised analysis), and any statistical methods to handle missing data (eg, multiple imputation)	11, 12, and in protocols A, B and C. These will also be detailed in a statistical analysis plans that will be uploaded to the online trial registrations.
**Methods: Monitoring**			
Data monitoring: formal committee	#21a	Composition of data monitoring committee (DMC); summary of its role and reporting structure; statement of whether it is independent from the sponsor and competing interests; and reference to where further details about its charter can be found, if not in the protocol. Alternatively, an explanation of why a DMC is not needed	Detailed in supplementary information files
Data monitoring: interim analysis	#21b	Description of any interim analyses and stopping guidelines, including who will have access to these interim results and make the final decision to terminate the trial	Detailed in supplementary information files
Harms	#22	Plans for collecting, assessing, reporting, and managing solicited and spontaneously reported adverse events and other unintended effects of trial interventions or trial conduct	Detailed in supplementary information files
Auditing	#23	Frequency and procedures for auditing trial conduct, if any, and whether the process will be independent from investigators and the sponsor	Detailed in supplementary information files
**Ethics and dissemination**			

For peer review only

Research ethics approval	#24	Plans for seeking research ethics committee / institutional review board (REC / IRB) approval	14
Protocol amendments	#25	Plans for communicating important protocol modifications (eg, changes to eligibility criteria, outcomes, analyses) to relevant parties (eg, investigators, REC / IRBs, trial participants, trial registries, journals, regulators)	14
Consent or assent	#26a	Who will obtain informed consent or assent from potential trial participants or authorised surrogates, and how (see Item 32)	14, and in protocols A, B and C
Consent or assent: ancillary studies	#26b	Additional consent provisions for collection and use of participant data and biological specimens in ancillary studies, if applicable	14, and in protocols A, B and C
Confidentiality	#27	How personal information about potential and enrolled participants will be collected, shared, and maintained in order to protect confidentiality before, during, and after the trial	Details in protocols A, B and C
Declaration of interests	#28	Financial and other competing interests for principal investigators for the overall trial and each study site	16
Data access	#29	Statement of who will have access to the final trial dataset, and disclosure of contractual agreements that limit such access for investigators	n/a
Ancillary and post trial care	#30	Provisions, if any, for ancillary and post-trial care, and for compensation to those who suffer harm from trial participation	Details in supplementary files for protocols A, B and C
Dissemination policy: trial results	#31a	Plans for investigators and sponsor to communicate trial results to participants, healthcare professionals, the public, and other relevant groups (eg, via publication, reporting in results databases, or other data sharing arrangements), including any publication restrictions	2, 14

For peer review only

Dissemination policy: authorship	#31b	Authorship eligibility guidelines and any intended use of professional writers	n/a
Dissemination policy: reproducible research	#31c	Plans, if any, for granting public access to the full protocol, participant-level dataset, and statistical code	14

Appendices

Informed consent materials	#32	Model consent form and other related documentation given to participants and authorised surrogates	Supplementary files in focused papers for protocol A, B and C (bmjopen-2020-040426, bmjopen-2020-040427, bmjopen-2020-040430)
Biological specimens	#33	Plans for collection, laboratory evaluation, and storage of biological specimens for genetic or molecular analysis in the current trial and for future use in ancillary studies, if applicable	n/a

None The SPIRIT checklist is distributed under the terms of the Creative Commons Attribution License CC-BY-ND 3.0. This checklist can be completed online using https://www.goodreports.org/, a tool made by the EQUATOR Network in collaboration with Penelope.ai

For peer review only
